# Transcriptional perturbation of LINE-1 elements reveals their *cis*-regulatory potential

**DOI:** 10.1038/s44319-026-00854-w

**Published:** 2026-07-08

**Authors:** Yuvia A Pérez-Rico, Anna Le Breton, Aurélie Bousard, Lenka Henao Misikova, Eskeatnaf Mulugeta, Sérgio F de Almeida, Alysson R Muotri, Edith Heard, Anne-Valerie Gendrel

**Affiliations:** 1 Directors’ Research, European Molecular Biology Laboratory, Heidelberg, Germany; 2 GIMM - Gulbenkian Institute for Molecular Medicine, Lisbon, Portugal; 3 Faculdade de Medicina da Universidade de Lisboa, Lisbon, Portugal; 4 Genetics and Developmental Biology Unit, Institut Curie, PSL Research University, CNRS UMR3215, INSERM U934, Paris, France; 5 Departments of Pediatrics and Cellular & Molecular Medicine, School of Medicine, University of California, San Diego, La Jolla, CA USA; 6 Collège de France, Paris, France; 7 Present Address: Department of Internal Medicine, Endocrine Tumor Laboratory, Erasmus University Medical Center Rotterdam, Rotterdam, The Netherlands; 8 Present Address: The Francis Crick Institute, London, UK

**Keywords:** Chromatin, Transcription & Genomics

## Abstract

Long interspersed element-1 (LINE-1 or L1) retrotransposons constitute the largest transposable element family in mammalian genomes and contribute prominently to inter- and intra-individual genetic variation. Although most L1 elements are inactive, some evolutionary younger elements remain intact and genetically competent for transcription and occasionally retrotransposition. Despite being generally more abundant in gene-poor regions, intact or full-length L1s (FL-L1) are also enriched around specific classes of genes and on the eutherian X chromosome. How proximal FL-L1 may affect nearby gene expression remains unclear. Here, we examine this systematically using engineered mouse embryonic stem cells (ESCs) in which expression of one active L1 subfamily is perturbed. We find that FL-L1 activation leads to the misregulation of ~1024 genes, whereas FL-L1 repression affects ~81 genes. In most cases (68%), misexpressed genes contain an intronic FL-L1 or lie near a FL-L1 (< 260 kb). Gene ontology analysis shows that upon L1 activation, upregulated genes are enriched for neuronal function-related terms, suggesting that some L1 elements may have evolved to control neuronal gene networks. These results illustrate the *cis*-regulatory potential of FL-L1 elements and suggest a broader role for L1s than originally anticipated.

## Synopsis

Genome-wide transcriptional repression and activation of full-length LINE-1 retrotransposons in mouse ES cells unmask their cis-regulatory potential on neighboring genes, notably within neuronal gene networks.

- Hundreds of protein-coding genes are misregulated following LINE-1 transcriptional perturbation, mainly L1MdTf activation.
- L1MdTf repression and activation predominantly impact genes within the same regulatory neighborhood.
- Neuronal genes are more vulnerable to L1MdTf cis-regulatory effects.

## Abstract (web summary)

Genome-wide transcriptional repression and activation of full-length LINE-1 retrotransposons in mouse ES cells unmask their cis-regulatory potential on neighboring genes, notably within neuronal gene networks.

## Graphical abstract

**Figure:**
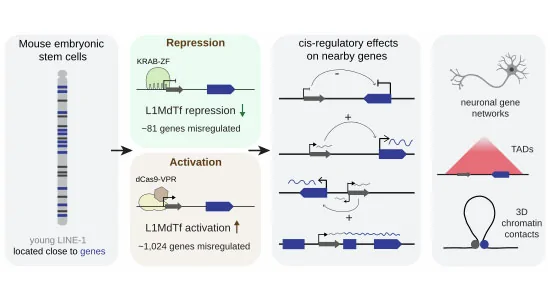

**Figure:**
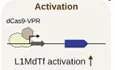

## Introduction

Transposable elements (TEs) constitute nearly half of mammalian genomes. These sequences have long been considered purely parasitic elements self-propagating in the host genome. However, it is now widely accepted that TEs are major drivers of genome evolution and genetic diversity, capable of expanding gene regulatory networks (Chuong et al, 2017), as originally proposed by Barbara McClintock (McClintock, 1956). TEs can be broadly classified into two classes: DNA transposons, which are mostly extinct in mammals, and retrotransposons, which transpose using a copy-paste mechanism and have massively contributed to the expansion of mammalian genome size. Among retrotransposons, long interspersed nuclear element-1 (LINE-1 or L1) constitute the most abundant TE family, representing ~20% of the human and mouse genomes and the only known autonomous class of TE mobile in humans (Hermant and Torres-Padilla, 2021).

The majority of L1 elements in mammalian genomes have been inactivated by the accumulation of mutations or truncations at their 5’ end. However, a small fraction of non-truncated L1 elements, known as full-length L1s (FL-L1s), still possess intact ORF1 and ORF2 sequences and retain the ability to retrotranspose. Additionally, a larger proportion of non-intact FL-L1s characterised by disrupted ORF sequences, therefore losing the ability to retrotranspose autonomously, populate mammalian genomes (Penzkofer et al, 2017). Many of these non-intact FL-L1s have retained a functional 5’UTR region and still have the potential to be transcriptionally active. As such, they may represent a hidden source of regulatory sequences capable of contributing massively to gene regulation and transcriptome diversity (Fueyo et al, 2022). Transcriptionally competent FL-L1s are stringently controlled both at the chromatin level, by epigenetic mechanisms and transcription factors, and at the post-transcriptional level. These mechanisms appear to act in a sequence-specific manner and depend on L1 evolutionary age or the cell type in which they operate (Bulut-Karslioglu et al, 2014; Castro-Diaz et al, 2014; Zoch et al, 2020). In the human genome, only ~150 L1 copies are still capable of retrotransposition, all of them belonging to the young, human-specific L1Hs subfamily, while thousands of L1 copies remain transcriptionally competent (Brouha et al, 2003; Penzkofer et al, 2017). In the mouse genome, three evolutionary young L1 subfamilies, called L1MdA, L1MdTf, and L1MdGf, remain active. They differ mostly in their 5’UTR sequences containing the promoter region. Collectively, these subfamilies comprise nearly 10,000 full-length elements that are potentially transcriptionally competent (Jachowicz and Torres-Padilla, 2016; Penzkofer et al, 2017; Sookdeo et al, 2013).

Uncontrolled activity of FL-L1s can have harmful consequences including DNA damage, genomic instability, gene disruptions and hindrance of the transcriptional coordination of gene networks. For that reason, FL-L1s are strongly silenced in somatic cells. Nevertheless, it has been observed that the activity of FL-L1s is enhanced upon aging and during malignancies (Payer and Burns, 2019). Moreover, specific contexts have revealed distinct transcriptional dynamics of FL-L1s. For instance, significant transcriptional activity of FL-L1s has been observed in embryonic stem cells (ESCs) and during preimplantation development (Jachowicz et al, 2017; Richardson et al, 2017) or in neuronal progenitor cells (NPCs) and neurons in the hippocampus (Richardson et al, 2014; Upton et al, 2015). These observations raised the possibility of a functional role conferred by FL-L1 activity, for example, during reprogramming following fertilization (Jachowicz et al, 2017) or in neuronal somatic mosaicism and plasticity, either coordinating regulatory gene networks or creating genomic diversity (Richardson et al, 2014). Additionally, a subset of FL-L1s are transiently expressed from the X chromosome during X-chromosome inactivation, rather than being silenced, which could facilitate gene silencing in specific regions of the chromosome (Chow et al, 2010; Loda et al, 2017). Building on these context-specific roles of FL-L1s, their integration into host gene regulation further underscores their potential functionality. Given their abundance in mammalian genomes, TEs and their remnants are frequently found near or within genes. In certain situations, they have been co-opted as functional *cis*-regulatory elements. This phenomenon has been well documented on a genome-wide scale, for certain ERV families and SVAs elements in mouse or human cells (Barnada et al, 2022; Fueyo et al, 2022; Hermant and Torres-Padilla, 2021). A previous study revealed that a significant portion of L1 repeats in mouse and human genomes are located within a short distance from genes (Lu et al, 2020). In addition, young L1 subfamilies are associated with local increase in chromatin accessibility in mouse ESCs (mESCs) (Ferraj et al, 2023). However, it remains unclear what fraction of FL-L1 elements directly contributes to gene regulation.

The recent expansion of genome editing technologies and computational tools have accelerated our access to TEs and the possibility to devise approaches for direct and systematic interrogation of their potential functions (Fueyo et al, 2022; Goerner-Potvin and Bourque, 2018). In recent years, various strategies have been developed to manipulate the expression of different classes of TEs, usually during early embryonic stages or in embryonic cells, contexts that are more permissive for their activity (Fueyo et al, 2022). For instance, a nuclease dead-Cas9 (dCas9) fused to effector domains was used to perturb the expression of LTR5Hs, a class of human endogenous retrovirus (ERVs), to show that these elements can act as early embryonic enhancers (Fuentes et al, 2018). TALEs (transcription activator-like effector) were used for transcriptional manipulation of endogenous L1 elements during mouse preimplantation development to demonstrate that L1 expression is critical for chromatin accessibility and developmental progression (Jachowicz et al, 2017). Finally, antisense oligonucleotides (ASOs) were used to target L1 RNA for degradation in mouse ES cells, which resulted in decreased self-renewal and proliferation, and demonstrated that L1 RNA appears to act as a nuclear RNA scaffold, regulating the expression of two-cell stage embryo genes (Percharde et al, 2018). In this study, we sought to investigate the extent to which FL-L1 retrotransposons may act as *cis*-regulators and shape gene regulatory networks. To answer these questions and considering the elevated transcriptional activity of L1 elements in mESCs (He et al, 2019), we used this model to engineer cell lines to perturb the expression of one representative young L1 subfamily in the mouse genome, L1MdTf. We report here the genome-wide specific repression and activation of targeted FL-L1s using zinc fingers-KRAB and dCas9-VPR systems. Hundreds of protein-coding genes were found to be misregulated following L1 perturbation, and these genes frequently contain an intronic L1 or lie near an L1 element targeted by the engineered effectors. Interestingly, we found that upregulated genes following L1 activation are enriched for neuronal function-related terms, in line with the observation that the activity of L1 elements is unleashed in neural progenitor cells and neurons (reviewed in (Bodea et al, 2018; Richardson et al, 2014)).

## Results

### Engineered repressor and activator to manipulate L1MdTf expression

**Figure 1:**
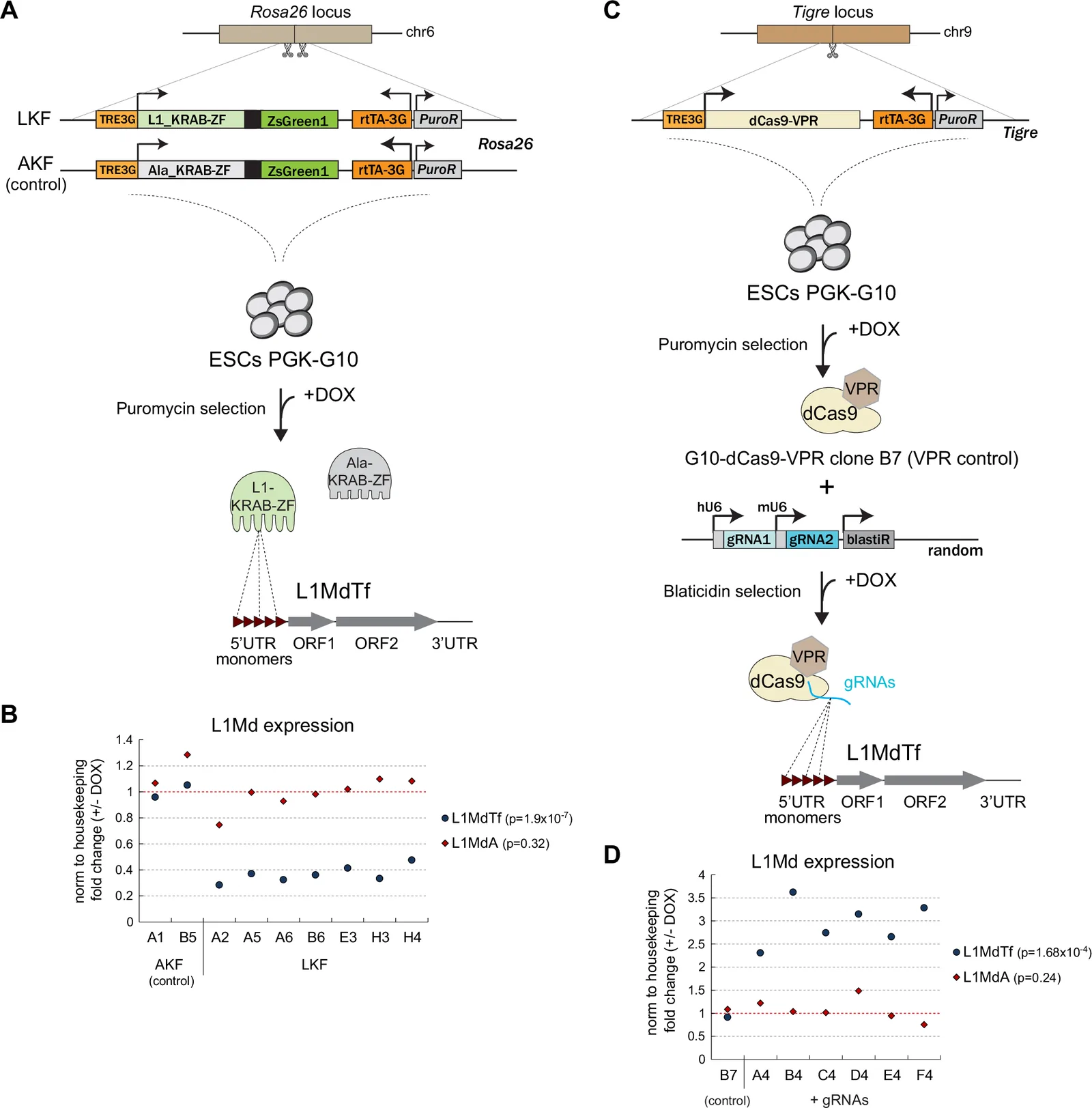
Targeting strategy for L1 expression perturbation in mouse ES cells. (**A**) Structure of the DOX-inducible LKF/AKF transgenes transfected in PGK-G10 cells for knock-in at *Rosa26*. (**B**) Following puromycin selection, colony picking and genotyping, 9 clones were analysed by qPCR for expression of the L1MdTf/L1MdA families using primers specific for the 5’UTR region, upon 48 h of DOX treatment. Significance was assessed using a one-sample *t* test comparing fold changes of all LKF clones analysed to 1 (null hypothesis: the observed mean equals the expected value of 1). (**C**) Structure of the DOX-inducible dCas9-VPR transgene transfected in PGK-G10 cells for knock-in at *Tigre*. (**D**) Following puromycin and blasticidin selections, colony picking and genotyping, 7 clones, including the control VPR-B7, were analysed for expression of L1MdTf/L1MdA upon DOX treatment (48 h) by qPCR. Significance was assessed using a one-sample *t* test comparing fold changes of all VPR+gRNAs clones analysed to 1 (null hypothesis: the observed mean equals the expected value of 1). Scissors indicate the approximate positions of gRNAs. TRE3G promoter Tet-responsive element 3rd generation, rtTA-3G DOX-inducible transactivator 3rd generation, PuroR puromycin resistance gene, BlastiR blasticidin resistance gene. hU6 human U6 promoter, mU6 mouse U6 promoter, ZF zinc fingers. Source data are available online for this figure.

**Figure EV1:**
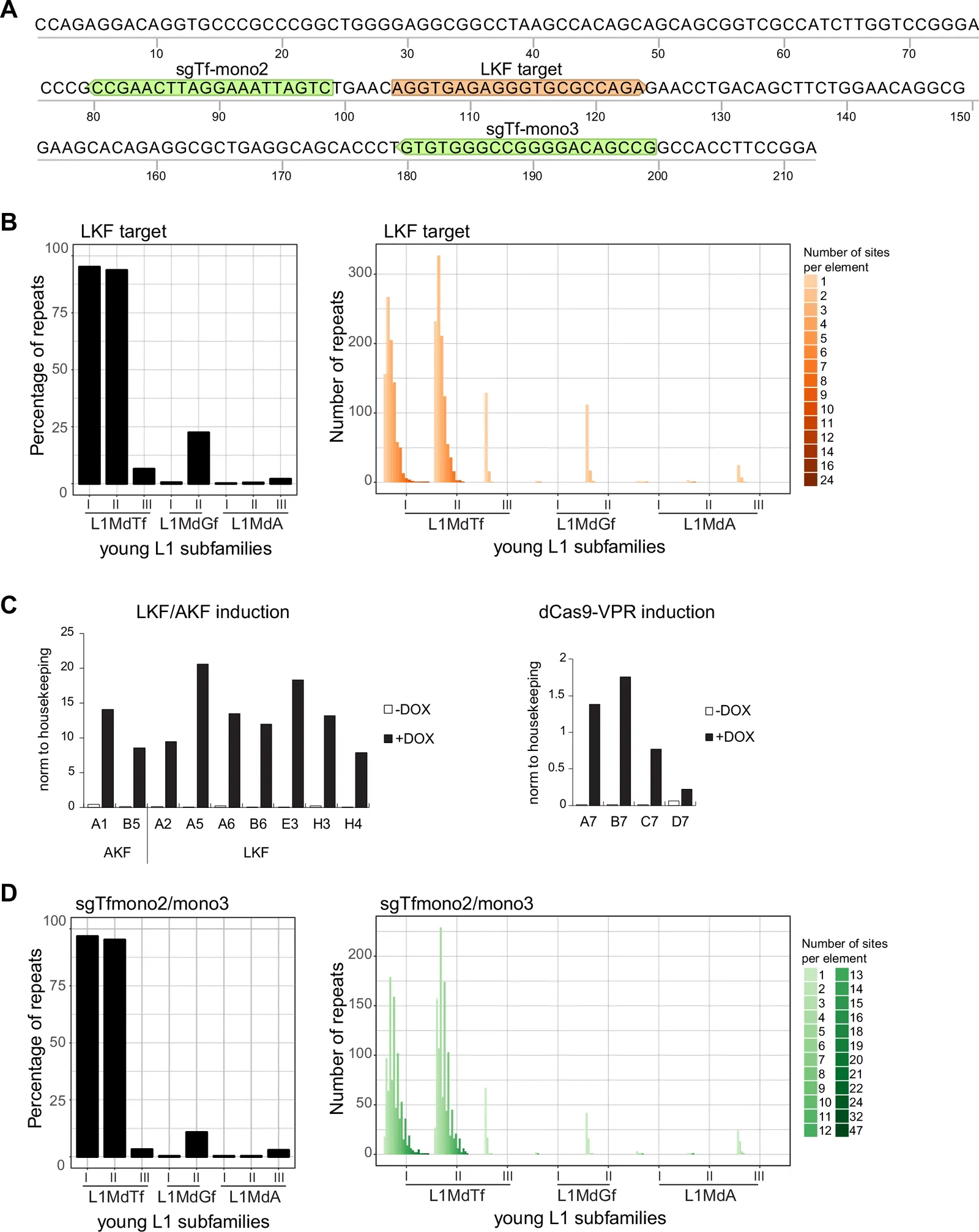
Identity and genomic representation of L1MdTf-repressor and activator predicted target sites. (**A**) Positions of LKF and sgRNAs (Tf-mono2 and Tf-mono3) target sequences within the 212 bp L1MdTf monomer consensus sequence. (**B**) Estimation of the percentage of young L1Md subfamilies bound by LKF repressor (left). Predicted number of elements from young L1Md subfamilies bound by LKF repressor according to the number of sites per element (right). (**C**) Following puromycin selection, colony picking and genotyping, 9 clones were analysed upon DOX treatment (48 h) by qPCR for expression of the LKF/AKF transgenes (left) and 4 clones for the expression of the dCas9-VPR transgene (right). (**D**) Estimation of the percentage of young L1Md subfamilies targeted by Tfmono2/3 guide RNAs (left). Number of elements from young L1Md subfamilies targeted by Tfmono2/3 according to the number of sites per element (right).

To investigate the impact of *cis*-regulation mediated by FL-L1 elements, we focused on the representative mouse young L1MdTf subfamily, which comprise an estimated number of 1722 non-exonic non-intact FL elements (commonly defined as >= 6 kb) in RepeatMasker annotations (2021-04-08 version). To achieve genome-wide repression of L1MdTf elements, we used an available fusion repressor (LKF) consisting of 6 zinc-fingers (ZF) engineered to bind a 20 bp region in the L1MdTf 5’UTR monomeric consensus sequence (Naas et al, 1998), fused to the KRAB repressor domain (Figs. 1A and EV1A). Preliminary analyses demonstrated that this ZF-KRAB construct was more effective at downregulating L1MdTf expression than dCas9 fused to KRAB, justifying its selection for these experiments. As a control, we used an analogous recombinant protein, carrying 4 amino-acid substitutions to alanine within each ZF, rendering them unable to bind DNA (called AKF). In silico analysis of the occurrence of the 20 bp region bound by each ZF predicted targeting of 1088 out of 1722 FL-L1MdTf, which comprise 93–95% of L1MdTf_I and L1MdTf_II and 6.5% of L1MdTf_III. In addition, 1000 L1MdTf shorter elements were predicted as targets, as well as 22.5% of the L1MdGf_II FL elements (69 elements) (Fig. EV1B). The majority (74.5%) of L1MdTf elements were predicted to contain more than one binding site for the LKF repressor (Fig. EV1B). Predicted off-target sequences outside L1 repeats were found only at 6 genomic locations, including 1 located 3 bp away from an annotated L1MdTf element (Dataset EV1). LKF and AKF repressor sequences were cloned upstream of an IRES-ZsGreen1 reporter, enabling a readout of expressing cells, under the control of doxycycline (DOX)-responsive promoter and knocked-in at the *Rosa26* locus in a PGK12.1 mESC line (PGK-G10) (Fig. 1A). Several independent clones were analysed by RT-qPCR and showed robust induction of the engineered repressor transgene (Fig. EV1C). In addition, using primers specific for the 5’UTR region, we observed a significant 2-fold downregulation of L1MdTf expression following 48 h of DOX treatment in LKF-expressing clones only, and no changes in expression of L1MdA (Fig. 1B).

To achieve genome-wide activation of L1MdTf elements, we relied on dCas9 fused to the VPR activation domain system, associated with two sgRNAs targeting two 20 bp sequences from the L1MdTf monomeric consensus sequence (Figs. 1C,D and EV1A). Similarly to the repression system, we checked the prevalence of the gRNAs targets in silico and found a total of 1080 FL-L1MdTf elements and 993 L1MdTf shorter copies predicted to be targeted by one or both gRNAs, accounting for more than 95% of L1MdTf_I and L1MdTf_II FL elements, the majority (93.9%) of which contain more than one gRNA-binding site (Fig. EV1D). Predicted off-target sites outside L1s were found at 17 locations, all within TEs (Dataset EV1). The dCas9-VPR transgene placed under the control of a DOX-inducible promoter was knocked-in at the *Tigre* locus into the PGK-G10 ESC line. Robust induction of the transgene upon 48 h of DOX treatment was assessed by RT-qPCR in independent clones, of which one was selected (VPR-B7, used as VPR-control in all subsequent analyses) for further random integration of the dual sgRNA-encoding transgene (Fig. EV1C). Analysis of independent clones showed significant activation of 2.5 to 3-fold specific for L1MdTf elements upon DOX induction of the dCas9-VPR. L1MdA expression, on the other hand, was not affected (Fig. 1D). These results indicate that the engineered repressor and activator targeting specific young L1 subfamilies lead to their general and robust repression or activation.

### Characterization of ESC lines encoding engineered L1MdTf-repressor and activator

**Figure 2:**
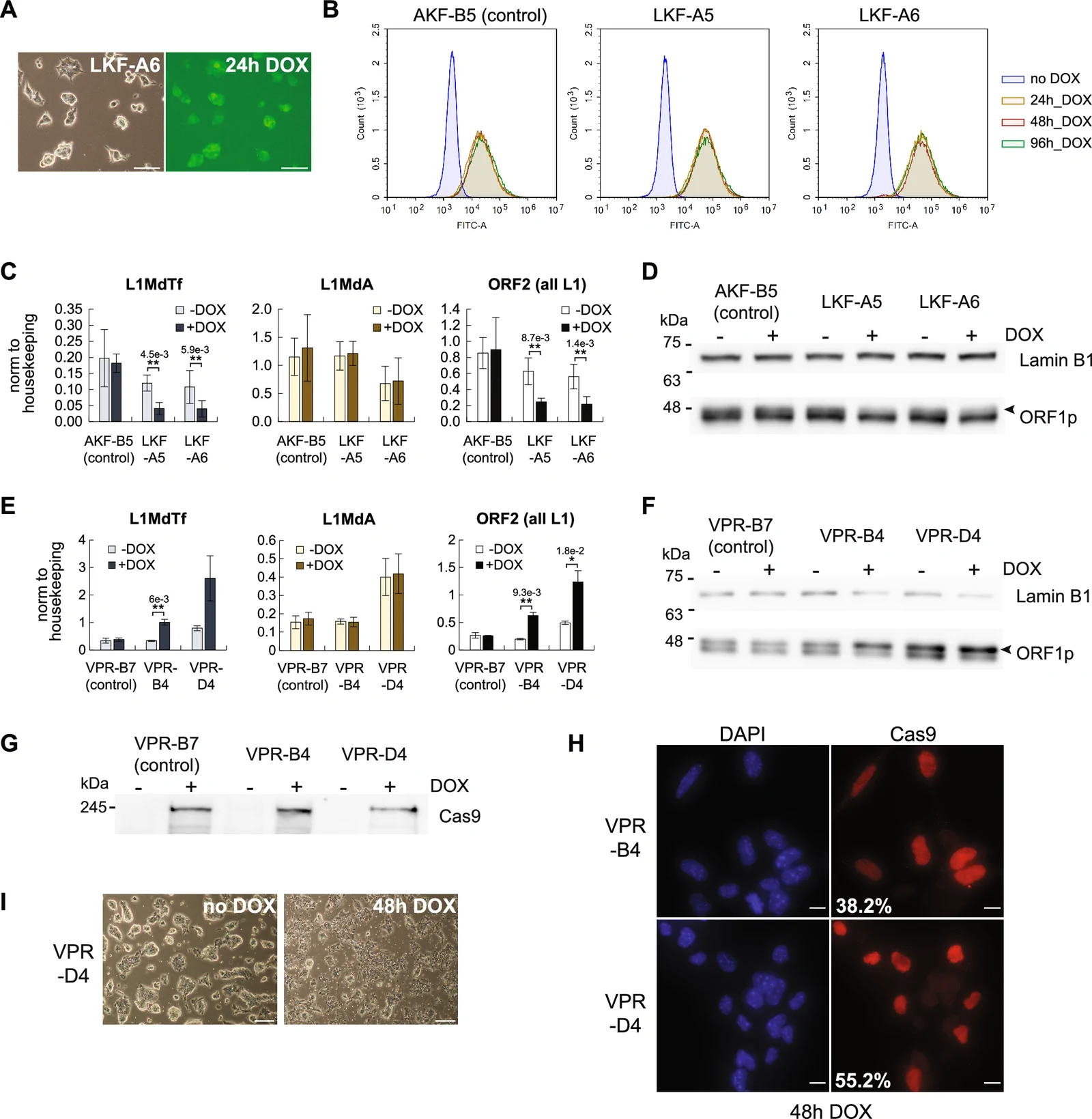
Characterization of ESCs showing L1MdTf expression perturbation. (**A**) Brightfield and fluorescence microscopy images of ESC colonies from repressor clone LKF-A6 showing ZsGreen1 signal after 24 h of DOX treatment (scale bars, 100 μm). (**B**) Flow cytometry analysis of repressor clones LKF-A5, LKF-A6 and control AKF-B5, showing similar fluorescence levels after DOX treatment for 24, 48 and 96 h. (**C**) qPCR analysis of L1 expression using primers for the 5’UTR of L1MdTf/L1MdA and the ORF2 region of all L1s, showing specific repression of L1MdTf in repressor clones LKF-A5, LKF-A6 and not in control AKF-B5, upon DOX treatment for 48 h. Levels are normalized relative to housekeeping gene expression. Error bars represent the calculated standard deviation considering five biological replicates and are centered at the mean normalized expression. (**D**) Western blot showing decreased ORF1p protein levels (top isoform, indicated with an arrow) in repressor clones LKF-A5 and LKF-A6, after DOX treatment for 48 h. LaminB1 is used as loading control. This experiment was repeated twice with similar results. (**E**) qPCR analysis of L1 expression using primers specific for the ORF2 region and the 5’UTR of L1MdTf/L1MdA, showing specific activation of L1MdTf in activator clones VPR-B4, VPR-D4 and not in control VPR-B7 upon DOX treatment for 48 h. Levels are normalized relative to housekeeping gene expression. Error bars represent the calculated standard deviation considering three biological replicates and are centered at the mean normalized expression. (**F**) Western blot showing increased ORF1p protein levels (top isoform, indicated with an arrow) in activator clones VPR-B4 and VPR-D4, after DOX treatment for 48 h. LaminB1 is used as loading control. This experiment was repeated twice with similar results. (**G**) Western blot showing induction of dCas9 after 48 h of DOX treatment in control VPR-B7 and activator clones VPR-B4 and VPR-D4. (**H**) Representative images of immunofluorescence showing induction of dCas9 after 48 h of DOX treatment in activator clones VPR-B4 and VPR-D4 (scale bars, 10 μm). Percentages indicate the number of cells with both high and low dCas9 signals. (**I**) Brightfield images of ESC colonies from activator clone VPR-D4 showing increased cell death after DOX treatment for 48 h (scale bars, 100 μm). Statistics: two-tailed paired *t* test (**C**, **E**): *P < 0.05, **P < 0.01; null hypothesis: there is no difference in expression between −DOX and +DOX conditions. Source data are available online for this figure.

Based on our initial RT-qPCR results (Fig. 1), we selected two clones showing efficient repression (LKF-A5 and -A6) and activation (VPR-B4 and -D4) of L1MdTf elements for further characterization, as well as one control clone for each system (AKF-B5, expressing the control repressor and VPR-B7, expressing no sgRNAs). First, we tested the DOX treatment duration to achieve maximal expression of the AKF/LKF repressors by analysing the ZsGreen1 reporter using microscopy and flow cytometry (Fig. 2A,B). The results showed that the expression was constant in the selected clones after 24, 48, and 96 h of treatment. Therefore, for all subsequent analyses, we decided to consider a DOX treatment of 48 h. We then analysed the repression of L1MdTf and L1MdA families by RT-qPCR. We confirmed that L1MdTf expression was significantly reduced in the LKF-expressing clones with DOX, whereas L1MdA expression was unaffected. Similar levels of repression were observed when using primers specific for the ORF2 region, able to recognize all L1 families (Fig. 2C), indicating that more than half of transcriptionally active L1 elements in mESCs are represented by L1MdTf elements. We also examined the repression at the protein level by measuring ORF1 protein, which is encoded by intact full-length L1s and present as isoforms. By Western blot, it was found that the levels of the top band corresponding to its p43 isoform are reduced in the LKF-expressing clones upon DOX treatment (Fig. 2D; Appendix Fig. S1A). This isoform mainly corresponds to the ORF1 protein of L1MdTf elements, while the bottom band represents the isoform mainly encoded by the coexisting L1MdA subfamily (Kolosha and Martin, 1995). This demonstrates specific L1MdTf repression at both RNA and protein levels, affecting both intact and non-intact elements.

**Figure EV2:**
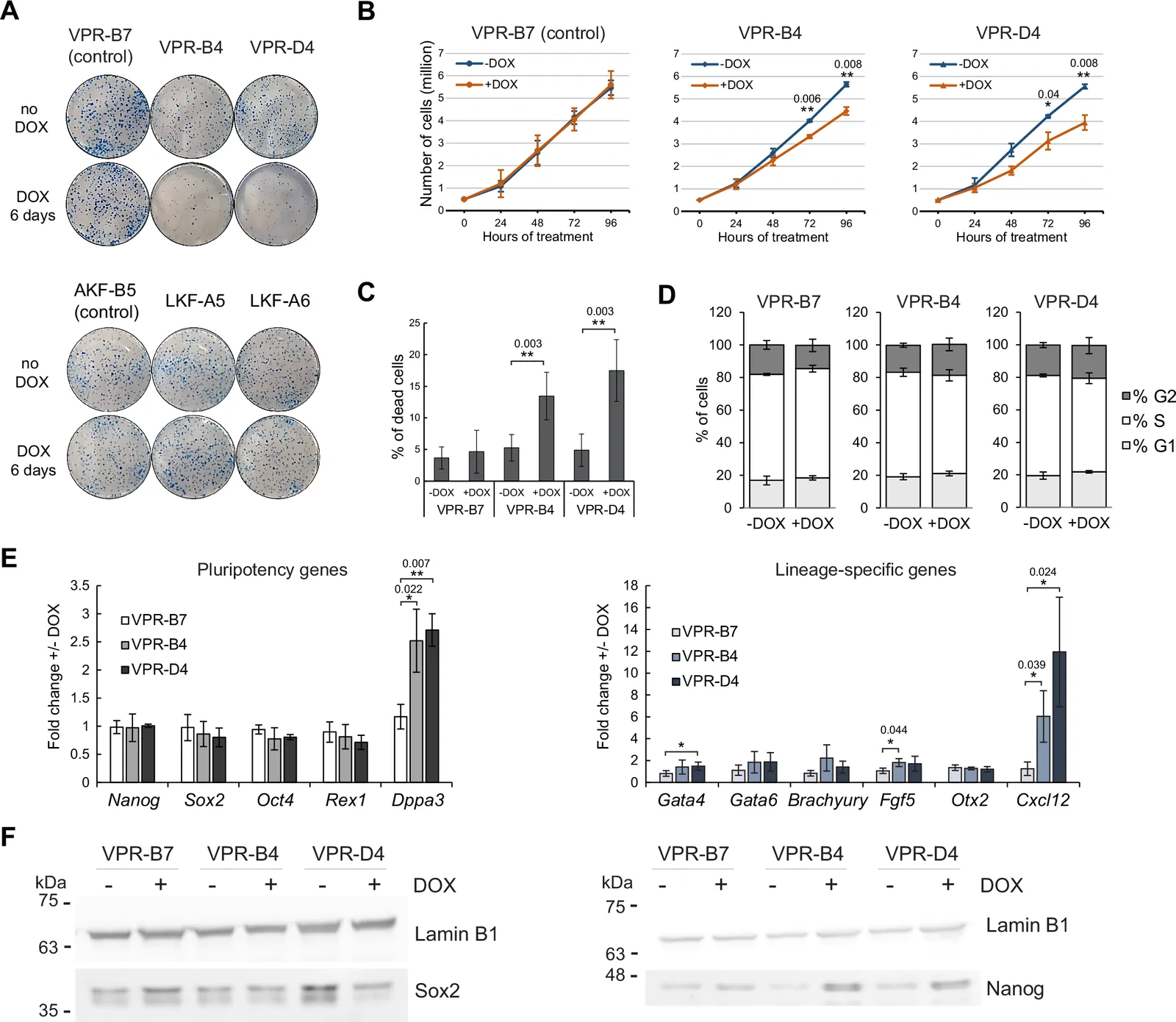
Characterization of ESCs with perturbed L1MdTf expression, including analyses of proliferation, cell death, cell cycle progression, and marker expression. (**A**) Representative images of colony formation assays in ESCs treated with or without DOX for 6 days, shown for both repressor and activator clones, as well as their respective controls. Colonies are marked by methylene blue stain. (**B**) Proliferation assay of VPR-B4, VPR-D4 and control clone, over 4 days with or without DOX treatment. Cells were counted every day. Data are shown as mean ± s.e.m. (*n* = 3 biological replicates). (**C**) Percentage of cell death calculated by counting Trypan blue-positive cells in VPR clones treated with or without DOX, every 24 h for 4 days. Data are presented as mean ± s.e.m. (*n* = 7). (**D**) Cell cycle analysis by flow cytometry using Propidium iodide staining, following 48 h of DOX treatment or no treatment in activator clones. Data are shown as mean ± s.e.m. (*n* = 3 biological replicates). (**E**) qPCR analysis of pluripotency and differentiation markers in untreated and DOX-treated (48 h) VPR-B7, VPR-B4 and VPR-D4 clones. Data are shown as mean ±  s.e.m. (*n* = 3 or 4 biological replicates). (**F**) Western blot analysis showing no change in Sox2 and Nanog levels in activator clones VPR-B4 and VPR-D4, after DOX treatment for 48 h. LaminB1 is used as loading control. This experiment was repeated twice with similar results. Statistics: two-tailed paired (**B**, **C**, **E**) *t* test: *P < 0.05, **P < 0.01; null hypothesis: there is no difference in cell proliferation, cell death and expression levels between −DOX and +DOX conditions.

We also performed RT-qPCR and Western blot analyses to confirm the specific activation of L1MdTf elements using dCas9-VPR. RT-qPCR using L1MdTf, L1MdA and ORF2-specific primers showed increased L1MdTf and ORF2 expression in the two VPR clones expressing sgRNAs, VPR-B4 and -D4, upon DOX treatment (Fig. 2E). Using Western blot analysis, we observed a mirror image to the one for L1MdTf repression, with the p43 isoform levels being more elevated in the VPR-B4 and -D4 clones upon DOX induction (Fig. 2F; Appendix Fig. S1A). As expected, after induction, dCas9 protein could be detected in all clones by Western blot and by immunofluorescence (IF) (Fig. 2G,H). Surprisingly, IF analysis showed that dCas9 expression was very heterogeneous in the three VPR clones analysed, with ~20% of cells showing high expression, some showing low expression, and others showing none (Fig. 2H). This heterogeneity prompted us to look at L1 nascent RNA and ORF1 expression at the single-cell level using RNA FISH and IF. RNA FISH was performed using a probe corresponding to the FL-L1spa element (Chow et al, 2010; Naas et al, 1998). L1spa RNA FISH signal appears as discrete dots within the nucleus and strong foci located mainly in the cytoplasm. The percentage of cells with these foci, their abundance, and size increased upon DOX induction in the VPR-B4 and -D4 clones (Appendix Fig. S1B). ORF1 protein was detected as bright foci in the cytoplasm of a fraction of cells prior to DOX treatment. Consistently, these foci were more abundant and observed in more cells after DOX induction (Appendix Fig. S1C). Quantification of both L1 RNA and ORF1 protein signal intensity indicated a higher value in VPR-B4 and -D4 clones upon DOX treatment, confirming the augmented transcriptional activity of L1 elements (Appendix Fig. S1B,C). Increase in ORF1 protein levels was not accompanied by higher levels of phosphorylated histone H2AX (γH2AX), a marker of DNA damage, suggesting no increase in L1MdTf retrotransposition events (Appendix Fig. S1D). Interestingly, dCas9-VPR-B4 and -D4 clones exhibited reduced cell proliferation compared to the control VPR clone and the LKF repressor-expressing clones (Fig. EV2A,B). In addition, both clones showed increased cell death after 24 h of DOX treatment, and this effect became more pronounced and statistically significant at 48, 72 and 96 h post-treatment (Figs. 2I and EV2C). However, we did not observe any changes in cell cycle progression in these two clones (Fig. EV2D). Finally, RT-qPCR and Western blot analyses of pluripotency and lineage-specific markers revealed only mild changes (Fig. EV2E,F), suggesting that ESC self-renewal remains largely unaffected, unlike what was previously reported upon L1 RNA degradation (Percharde et al, 2018). Interestingly, we observed upregulation of the maternal gene *Dppa3* upon L1MdTf upregulation, which was shown to promote the 2-cell embryo expression program (Zhang et al, 2022).

### Engineered effectors affect expression of a large number of L1MdTf elements

**Figure 3:**
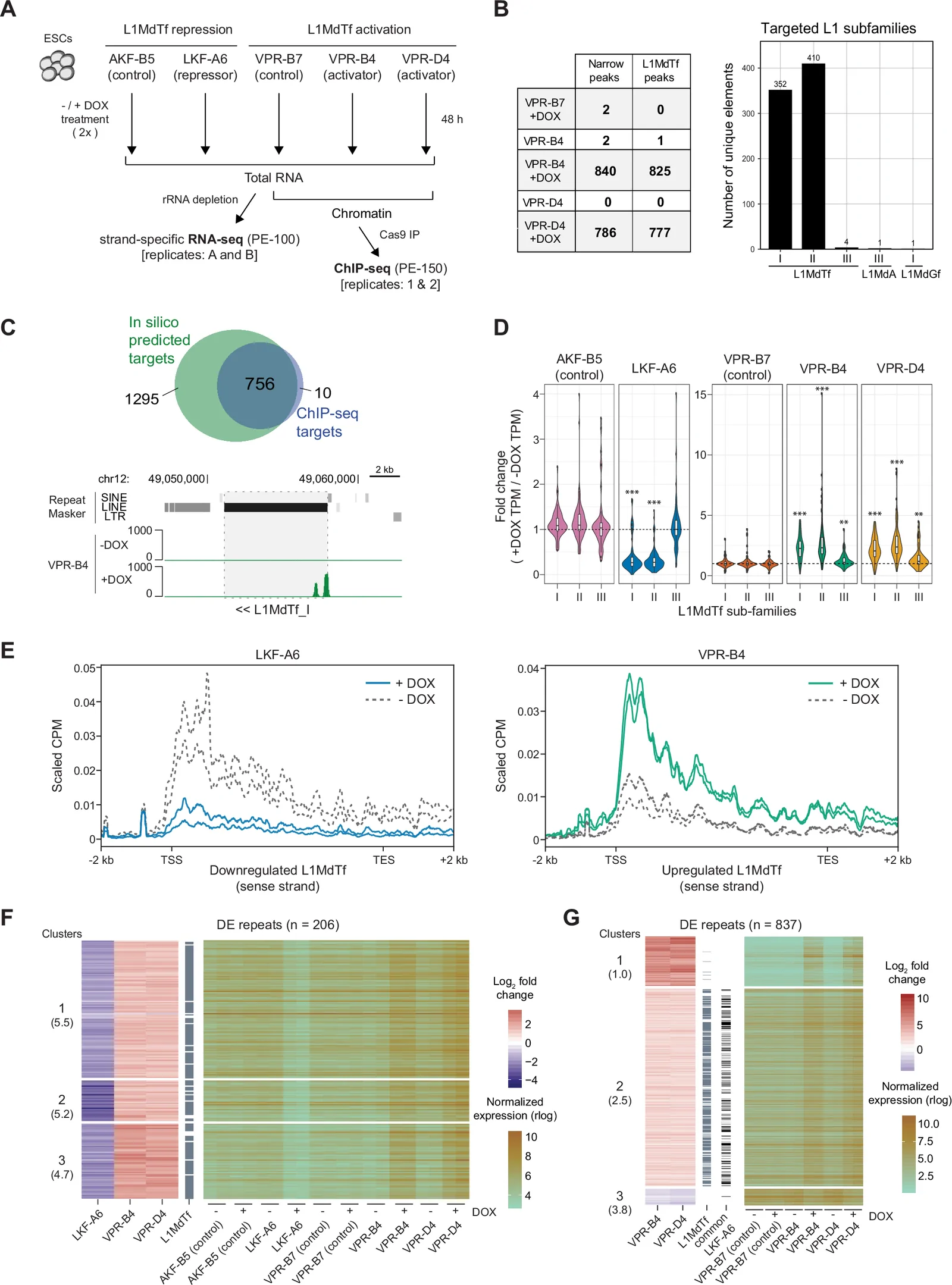
Differential expression of L1MdTf and repeats upon L1MdTf repression and activation. (**A**) Scheme of RNA-seq and ChIP-seq data generation from ESC selected clones treated with or without DOX. (**B**) Results of ChIP-seq analyses using uniquely mapped reads only. Left: number of total narrow peaks and those overlapping L1MdTfs in VPR clones. Right: estimation of the number of young L1Md unique elements bound by dCas9. (**C**) Top: Venn diagram showing overlap between in silico L1MdTf predicted targets and observed targets using uniquely mapped ChIP-seq reads. Bottom: example of a DOX-induced dCas9 peak within the 5’UTR of an L1MdTf located on chromosome 12. (**D**) Violin plots representing the distribution of expression fold changes for individual L1MdTf repeats classified by subfamily in all clones. Dashed lines indicate values equal to 1. Box plots show the 25th, 50th and 75th percentiles of fold changes. The inter-quantile range (IQR) is defined as the difference between the 75th and 25th percentiles. The upper whisker extends to the largest value within 1.5× IQR above the third quartile, and the lower whisker extends to the smallest value within 1.5× IQR below the first quartile. Values beyond the whiskers are shown as outlier points. The number of L1MdTf_I, II, and III elements shown in violin and box plots are 77, 113 and 57, respectively. Significance was assessed using one-sided Wilcoxon rank-sum test; null hypothesis: control distributions for each subfamily have lower/greater mean than repressor/activator clones; ***P < 1 × 10^−10^, **P < 1 × 10^−3^. Exact *P* values of significant differences shown from left to right: 3.02e-18, 6.56e-34, 2.99e-15, 1.27e-27, 2.43e-4, 3.58e-16, 1.43e-26, 7.91e-4. (**E**) Metagene plot of normalized RNA-seq signal (shown for LKF-A6 and VPR-B4, replicates A and B) on L1MdTf repeats showing downregulation in the LKF-A6 clone (left) or upregulation in the activator clones (right) upon DOX treatment. The *x* axis is scaled to represent full annotations and the 2 kb upstream and downstream regions. (**F**) Heat maps showing expression fold change (left) and normalized expression (right) of common DERs identified in repressor and activator clones. DERs are organized according to their cluster classification. Average normalized expression of genes in the −DOX condition of all displayed clones is indicated below each cluster number. Grey bars indicate if the repeat belongs to an L1MdTf subfamily. (**G**) Heat maps showing expression fold change (left), normalized expression (right) of DERs identified in both activator clones and L1MdTf annotation, as described in (**F**). Black bars indicate if the repeat is also identified as a DER in the LKF-A6 clone. CPM counts per million reads mapped, TSS transcription start site, TES transcription end site. Source data are available online for this figure.

To assess the specificity of the effectors and evaluate possible off-target binding, we first analysed the genome-wide binding of the dCas9-VPR effector by performing chromatin immunoprecipitation followed by sequencing (ChIP-seq), using an antibody against Cas9; ChIP-seq was not performed for the LKF repressor clones due to the lack of a suitable antibody. This experiment was performed in the VPR clones -B4 and -D4 before and after DOX treatment, as well as in the VPR-B7 control (with DOX treatment only) (Fig. 3A). The 150 bp paired-end sequencing allowed mapping the signal to individual L1 repeats using uniquely mapped reads. The results indicate highly specific targeting of dCas9 to L1MdTf elements (~800 narrow peaks per clone; MACS2 signal value > 18), the majority of which being L1MdTf_I and L1MdTf_II FL elements, consistent with our in silico prediction (Fig. 3B). The number of off-target binding sites is minimal, occurring within other L1 annotations and five genes (three in common between the two clones) (Dataset EV2). Although ChIP-seq quality varied between biological replicates of the DOX-treated VPR clones -B4 and -D4, we identified multiple peaks within individual elements, corresponding to targeting of several monomers (Fig. 3C). In addition, comprehensive analysis of the ChIP-seq data, including multimapping reads assigned to one of their top alignments, confirmed a limited number of off-target sites. Most of the peaks from the multimapping analysis overlap predicted binding sites and only two additional peaks were exclusively detected by ChIP-seq (Appendix Fig. S2A).

Next, to analyse genome-wide L1MdTf repression or activation and its impact, we performed 100 bp paired-end total RNA-seq (stranded, with rRNA depletion) on five of the previously characterised ESC clones: one control (AKF-B5) and one repressor clone (LKF-A6), plus one control (VPR-B7) and two activator clones (VPR-B4 and VPR-D4). For all cell lines, two biological replicates were sequenced, before and after DOX induction (Fig. 3A). RNA-seq analysis was performed following a standard pipeline, using two mapping strategies to report only uniquely mapped reads or multimapped reads randomly assigned to one genomic location. The first strategy was used to examine transcriptional changes of genes and specific repeat elements, while the second strategy was used as a proxy to analyse the expression of different repeat families from the Repeat Masker database. Approximately 12.5% of reads in each library were mapped to multiple locations, of which an average of 20% corresponds to non-exonic repetitive sequences. No global significant changes were observed for the L1 family in the repression samples (Appendix Fig. S2B). However, consistent changes after DOX treatment were observed for L1s in the VPR activation system, as normalized counts for L1 sequences were significantly increased for the VPR-B4 and -D4 libraries with DOX (Appendix Fig. S2C). LTR/ERV and SINE/B2 families showed fluctuating counts, but this was uncorrelated with the repressor/activator expression or DOX treatment (Appendix Fig. S2B,C; Dataset EV3). We then analysed the fold change of L1 repeats counts by subfamily (Appendix Fig. S2D; Dataset EV4). In the absence of DOX treatment, we detected a basal expression of 800 to 1500 L1MdTf elements across L1MdTf_I, _II, and _III subfamilies (Dataset EV5). Upon DOX-induced L1 repression and activation, the most affected subfamilies were the evolutionarily youngest ones: L1MdTf_I and L1MdTf_II. The L1MdTf_III, L1MdGf_II and L1MdA_III subfamilies were also impacted but to a lesser extent (Appendix Fig. S2D). Importantly, this was consistent with our initial in silico prediction (Fig. EV1B,C). We then analysed the average fold-change over individual elements from each L1MdTf subfamily, for which uniquely mapped reads could be identified with confidence in both RNA-seq libraries. In line with our prior qPCR analysis (Fig. 2C,E), repression of L1MdTf_I and L1MdTf_II elements is about four-fold in the LKF-A6 clone, and their activation is around three-fold in the VPR-B4 and -D4 libraries relative to AKF-B5 and VPR-B7 controls, respectively (Fig. 3D). L1MdTf_III elements were significantly affected to a smaller degree in VPR clones, but not in LKF-A6 (Fig. 3D).

To identify changes in the RNA-seq signal distribution along L1MdTf elements genome-wide, we determined their average sense and antisense profiles in all libraries using uniquely mapped reads. As some of the L1 annotations may not be perfectly delimited, we analyzed their expression using the annotated coordinates and a + 2 kb window from the start and end of the element. Signal in sense direction was detected across the whole element, with higher coverage just downstream of the TSS, as expected because of the greater sequence diversity of the 5’UTR/promoter region among L1Md families. This trend was strongly enhanced by VPR induction and reduced upon expression of the repressor (Fig. 3E; Appendix Fig. S3A). Interestingly, we also noticed read-through transcription after the 3’UTR towards downstream sequences (Fig. 3E; Appendix Fig. S3A) and antisense transcription originating from the L1-promoter region towards upstream sequences in all samples (Appendix Fig. S3B). Both antisense and read-through transcription were affected by the engineered effectors upon DOX treatment, indicating that this is a direct consequence of L1 expression (Fig. 3E; Appendix Fig. S3A,B). This confirms prior observations of an antisense promoter in mouse L1 elements and weak polyadenylation signals (Goodier et al, 2001; Li et al, 2014).

Differential expression analysis considering specific L1MdTf elements showed a significant correlation between the number of LKF or gRNA target sites in L1MdTfs and their expression fold change after DOX treatment, indicating that the simultaneous binding of dCas9 or the LKF repressor in the same region enhances activation or repression (Appendix Fig. S3C). Overall, using unique mapping, we found 513 individual repeat annotations differentially expressed (adjusted *P* value (*P*adj < 0.01) in the LKF-expressing clone, with a substantial fraction (*n* = 377 out of 1023 elements tested) corresponding to repressed L1MdTf annotations (Dataset EV5). In the VPR-B4 and -D4 clones, we found 1400 and 1421 repeat annotations with significant changes in expression (*P*adj < 0.01), respectively, of which a large part corresponds to upregulated L1MdTf elements (*n* = 709 out of 2315 for VPR-B4; *n* = 659 out of 1815 for VPR-D4; Dataset EV5). Importantly, there is an extensive overlap (*n* = 837) between differentially expressed repeats (DERs) in the two VPR clones (Appendix Fig. S3D), indicating that the observed effects are reproducible. By comparing the DERs in all clones, we identified 206 repeat annotations showing reciprocal expression changes mediated by the engineered effectors and corresponding mostly to L1MdTf elements (Fig. 3F). Clustering these repeats by their fold change showed that the L1MdTf repeats with stronger repression in the LKF-A6 clone (cluster 2) do not correspond to the L1MdTf repeats with stronger activation in the VPR clones (cluster 3), possibly due to the different effector systems that were used or the initial expression levels in ESCs. Indeed, the more strongly activated repeats (cluster 3) tend to have lower normalized expression prior to DOX treatment (Fig. 3F). We also observed this trend when clustering the common DERs in the VPR clones. However, in this case, the cluster mainly included members of other L1 subfamilies (cluster 1) (Fig. 3G). As only a few predicted gRNA binding sites and observed dCas9 binding sites do not overlap L1MdTfs, this result suggests that increased expression of non-L1MdTf L1 repeats may be a secondary effect of L1MdTf upregulation rather than direct activation mediated by the dCas9 effector. The largest cluster of DERs in the VPR clones includes mainly L1MdTf_I and L1MdTf_II annotations that show higher basal expression levels and are repressed in the LKF-A6 clone (cluster 2). Importantly, we also identified a small fraction of downregulated repeats characterized by their high basal expression and corresponding to ERVK/ERVL annotations (cluster 3) (Fig. 3G). Altogether, these results indicate that the effector systems allow the specific modulation of L1MdTf expression genome-wide and suggest L1MdTf roles in the regulation of other repeat subfamilies.

### L1MdTf misregulation influences nearby host gene expression

**Figure 4:**
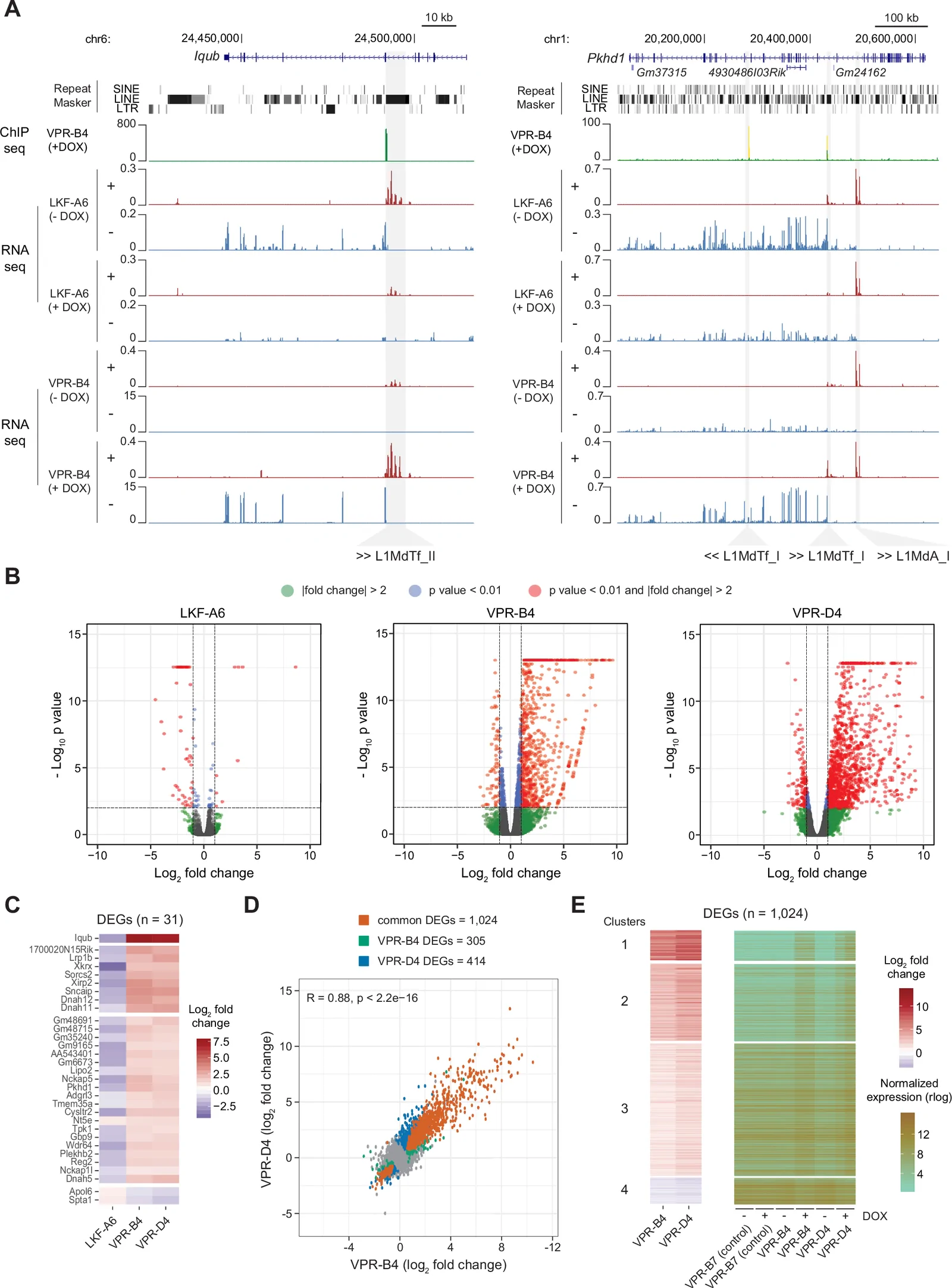
Differential expression of genes upon L1MdTf repression and activation. (**A**) Examples of downregulated and upregulated genes following the repression and activation of L1MdTf repeats, respectively. ChIP-seq for dCas9 (VPR-B4 clone, +DOX) profile is shown in green (uniquely mapped reads) and yellow (multimapped reads, not shown to scale). RNA-seq profiles by strand are shown in red (+ strand) and blue (− strand) for the DOX-treated and untreated samples. Intronic L1MdTf repeats with reciprocal expression changes and an L1MdA repeat with no expression changes, and their orientation, are highlighted to exemplify the specific targeting. (**B**) Volcano plots showing gene expression fold changes (*x* axis) and their significance (*y* axis) in the repressor (*n* = 18,904) and activator clones (for VPR-B4, *n* = 21,941; for VPR-D4, *n* = 19,466). *P* values were derived from DESeq2 Wald test statistics (Z-scores) and corrected for multiple testing using the Benjamini–Hochberg method. (**C**) Heat map depicting the fold changes of common DEGs identified in the repressor and activator clones. Genes are clustered based on their fold changes. (**D**) Comparison of expression fold changes of genes in the VPR-B4 and VPR-D4 clones after DOX treatment. Grey dots represent genes with no significant changes in expression and colored dots represent genes identified as DEGs in both clones or in one of the clones only. Pearson correlation coefficient and significance from *t* test are indicated; null hypothesis: the correlation is due to chance. (**E**) Heat maps showing expression fold change (left) and normalized expression (right) of common DEGs identified in both activator clones. DEGs are organized according to their cluster classification. Source data are available online for this figure.

By displaying the RNA-seq signal profiles, we observed that expression changes of L1MdTf DERs coincide with changes in the expression of genes in their proximity, as exemplified by the *Iqub* and *Pkhd1* genes which both contain intronic L1MdTf elements (Fig. 4A). Therefore, we evaluated the impact of the genome-wide perturbation of L1MdTf elements on host gene expression using our RNA-seq datasets. For the repression system, we observed a mild impact on gene expression, with 91 differentially expressed genes (DEGs) (*P*adj <0.01) between the DOX and no DOX conditions for the LKF-expressing clone, of which 10 are also DEGs in the control repressor clone, thus likely sensitive to the DOX treatment alone (Appendix Fig. S4A; Dataset EV5). Among the 81 remaining genes, the majority (n = 65) are downregulated (Fig. 4B). For the activation system, the impact on gene expression was much stronger, with 1384 and 1457 DEGs (*P*adj <0.01) between DOX and no DOX conditions for the VPR-B4 and -D4 clones, respectively (Fig. 4B). A minimal number of DEGs were also detected in the control activator clone (<=14) and were excluded from all further analyses (Appendix Fig. S4A; Dataset EV5). Of note, 31 DEGs were common between the repression and activation systems, with almost half of the downregulated genes in the LKF-A6 clone (29/65) showing upregulation in the VPR clones, as illustrated by the *Iqub* and *Pkhd1* genes (Fig. 4A,C). In the VPR clones, similar to what we observed previously for the DERs, the majority of DEGs upon DOX treatment are found in both clones (*n* = 1024) and are upregulated (*n* = 923) (Fig. 4B,D,E). We found that, overall, affected genes do not show apparent clustering and are located across all chromosomes. There were, however, some notable exceptions, including the X chromosome that showed a twofold enrichment for upregulated genes (105 DEGs) compared to autosomes (43 DEGs on average), correlating with the twofold enrichment of L1 repeats on the X chromosome (number of L1MdTf > 6 kb in chrX=162; average number of elements in other chromosomes = 79). Additionally, we observed the upregulation of small clusters of family-related genes (e.g., GABA receptor subunit, Keratin, Mage) and a large cluster of homeobox genes located on the X chromosome, the *Rhox* cluster (Appendix Fig. S4B). Similarly to DERs, genes with higher fold change upon VPR expression displayed lower expression levels in the -DOX condition (cluster 1 and 2) (Fig. 4E). We selected eight genes among the top differentially expressed, including DEGs showing reciprocal repression or activation as well as X-linked genes, and performed RT-qPCR analysis. We confirmed the observed expression changes in both directions upon DOX treatment using independent biological replicates (Appendix Fig. S4C,D).

**Figure 5:**
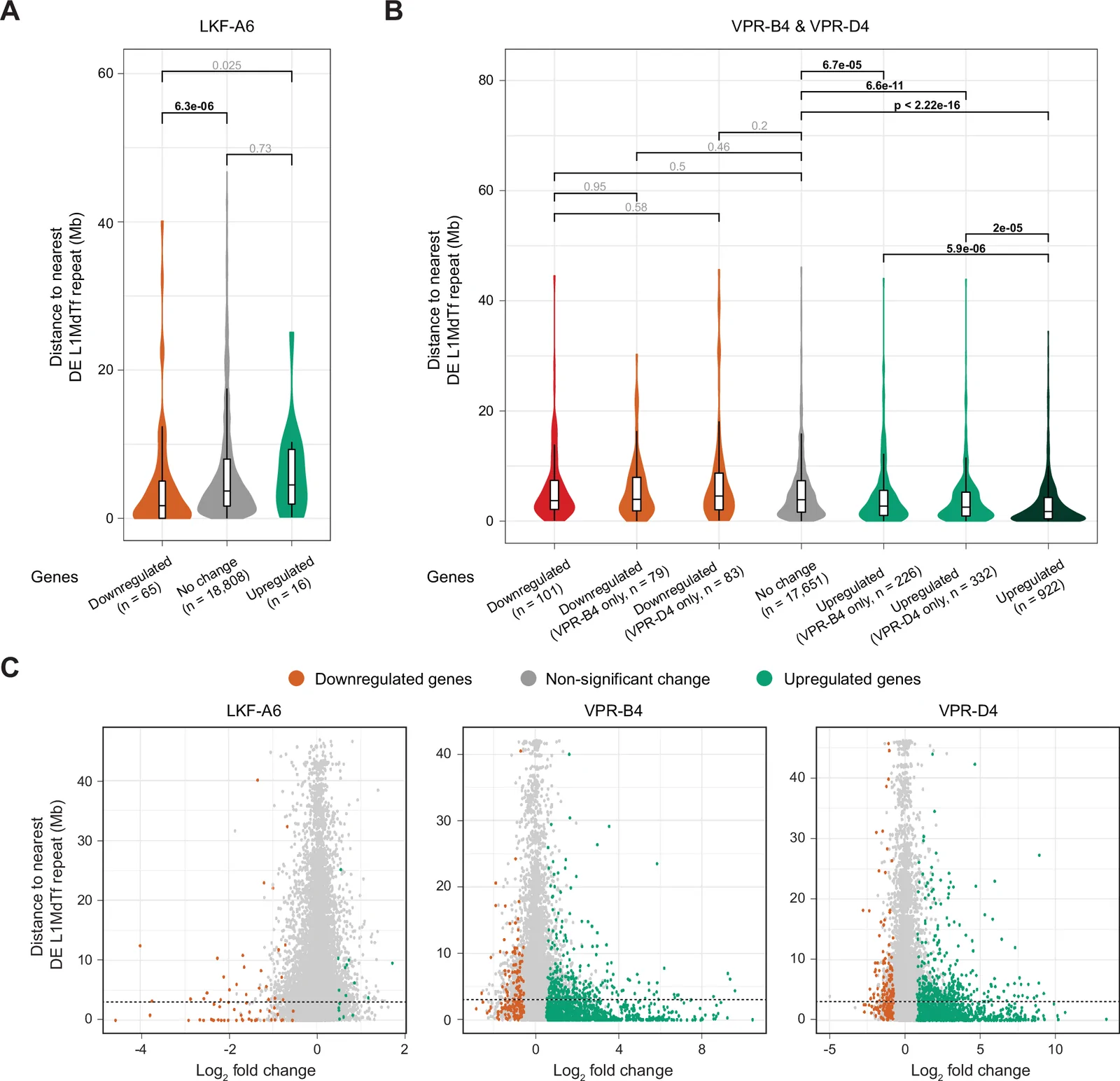
Distance analysis between DEGs and DE L1MdTf repeats. (**A**) Distribution of distances between DE L1MdTf repeats and genes classified according with DE analysis results of the LKF-A6 clone. The number of genes (*n*) included in each violin and box plot varies and is indicated in the *x* axis labels. Box plots show the 25th, 50th and 75th percentiles of fold changes. The inter-quantile range (IQR) is defined as the difference between the 75th and 25th percentiles. The upper whisker extends to the largest value within 1.5× IQR above the third quartile, and the lower whisker extends to the smallest value within 1.5× IQR below the first quartile. Significance values of the differences between distributions are displayed above violin plots and were calculated using two-sided Wilcoxon rank-sum tests; null hypothesis: the compared distributions differ by a location shift of zero (statistically significant values are in bold). (**B**) Distribution of distances between DE L1MdTf repeats and genes classified according with DE analysis results of the activator clones. The number of genes (*n*) included in each violin and box plot varies and is indicated in the *x* axis labels. Box plot representations and significance values as described for (**A**). (**C**) Comparison between expression fold change upon L1MdTf repression (LKF-A6) or activation (VPR-B4, VPR-D4) and distance to nearest DE L1MdTf repeat for all genes tested for DE. Dashed lines indicate a distance of 3 Mb. Source data are available online for this figure.

**Figure EV3:**
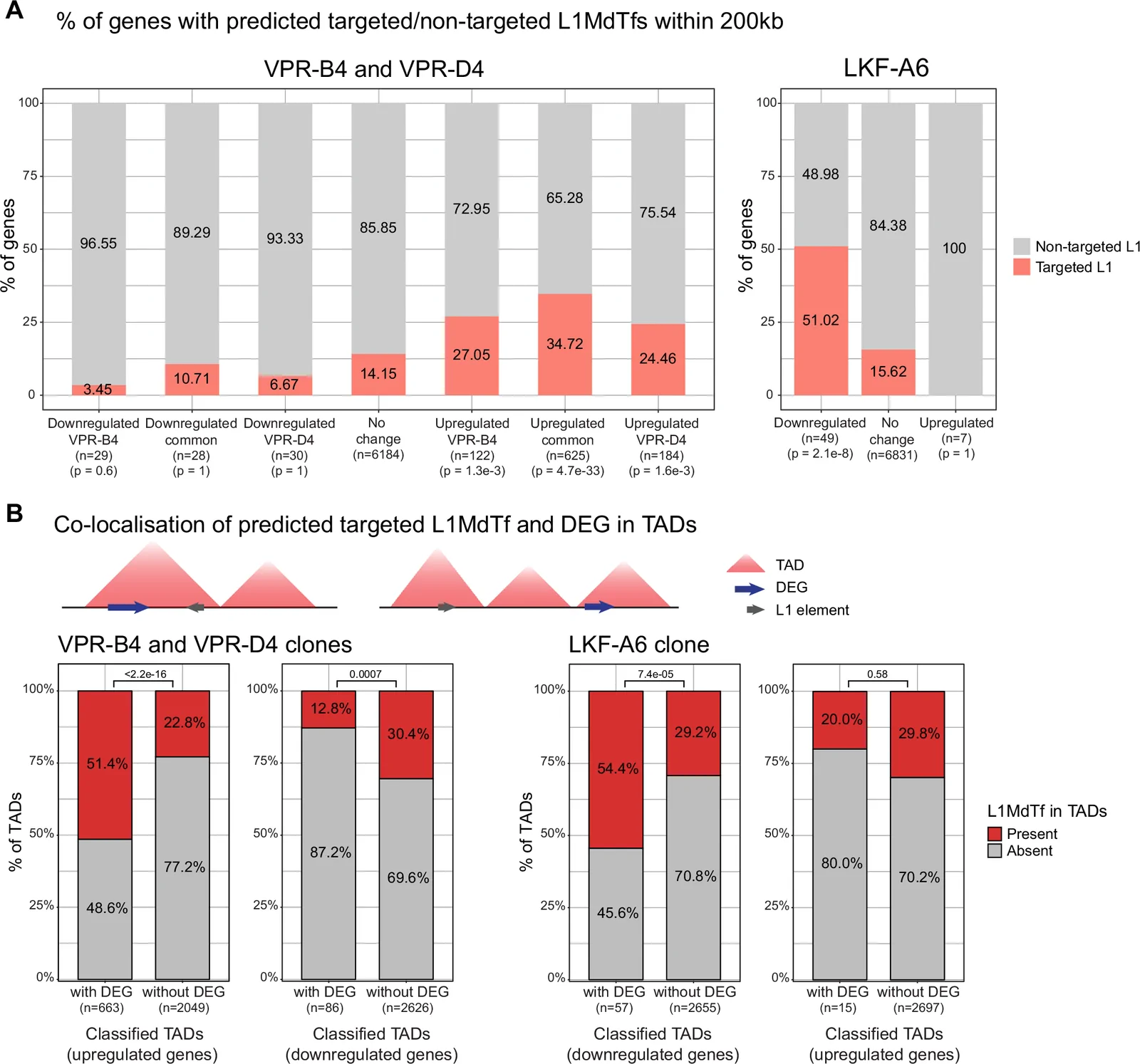
Impact of predicted targeted L1MdTf on gene expression and TAD co-localisation. (**A**) Percentages of genes (up-, downregulated, or with no change) with predicted targeted (red) versus non-targeted (grey) L1MdTfs within 200 kb of their vicinity in VPR-B4, VPR-D4 and LKF-A6 clones. The number of genes considered for each category and clone are indicated. *P* values were calculated using Fisher’s exact test, with the “No change” category as reference, followed by Bonferroni correction for multiple comparisons; null hypothesis: transcriptional status is independent of proximity to predicted targeted/non-targeted L1MdTfs. (**B**) Proportion of predicted targeted L1MdTf elements located in the same TAD as upregulated or downregulated genes in VPR-B4/VPR-D4 (left) and in LKF-A6 (right) clones. The number of TADs considered for each category and clone are indicated. *P* values above the histograms were calculated using Chi-squared tests of independence (with Yates’ continuity correction) to assess associations between L1MdTf presence/absence and DEG presence/absence in TADs; null hypothesis: no association between TAD annotations based on DEGs and L1MdTfs with predicted binding sites. The top diagram illustrates TAD annotation.

Given our observation that activation and repression of L1MdTf elements appear correlated with activation/repression of nearby genes (Fig. 4A), we next examined the genomic distance between DEGs or DERs and misregulated L1MdTf elements. We first plotted the distance distribution between DE L1MdTfs and DEGs or DERs. Overall, we found that the average distance values of LKF-downregulated (~3.9 Mb) and VPR-upregulated (~3.9 Mb) genes to DE L1MdTf elements are lower than those of LKF-upregulated (~5.6 Mb), VPR-downregulated (~6.3 Mb), or genes whose expression remains unaffected (Fig. 5A,B; Dataset EV6). Moreover, this tendency was clearer for commonly upregulated genes in both VPR clones (~3.1 Mb) than for genes upregulated only in one of the 2 clones (~4.2 Mb) (Fig. 5B). Similar results were observed for non-L1 DERs down- or upregulated in LKF and VPR clones, respectively (Appendix Fig. S5A,B; Dataset EV6). Additionally, when comparing the fold change and the genomic distance between affected L1s and misregulated genes (for both LKF and VPR clones) or non-L1 DERs (for the VPR clones only), we observed that the genes or repeats showing the highest fold change upon DOX (the 90th percentile) tend to be located within an average distance <3 Mb from an L1MdTf DER (Fig. 5C; Appendix Fig. S5C). This rather large average distance may be due to the fact that we can only analyse a limited number of specific L1MdTf using uniquely mapped reads due to the repetitive nature of these sequences, and probably, the proportion of DEGs and non-L1 DERs located close to affected L1MdTf repeats is underestimated. In fact, when comparing the fold changes of DEGs and the distance to all annotated L1MdTf elements, we observed that most of the strongly LKF-downregulated and VPR-upregulated genes are located within an average 260 kb distance from an L1MdTf element (Appendix Fig. S5D). For this reason, we tested if, at shorter distances (<=200 kb), the presence of a DE L1MdTf correlates with expression changes of genes and non-L1 repeats. This analysis showed significant differences from expected in the distribution of genes and repeats classified according to their expression changes and distance to DE L1MdTf elements (*P* value < 1 × 10^−3^, chi-squared test of independence) (Appendix Fig. S5E,F). Indeed, most of these differences were driven by the downregulated (30.9% and 30.3% (values relative to DE only)) and the commonly upregulated (35.9% and 9.2%) genes and repeats in the LKF and VPR clones, respectively. To leverage the larger set of in silico predicted binding sites at L1MdTfs (despite fewer DE calls due to repetitiveness), we next calculated the percentage of DEGs whose closest L1MdTf (within a 200 kb window of their gene body) is either a predicted targeted or a non-targeted element. We found that out of the 625 common VPR-upregulated genes with an L1MdTf within 200 kb, 34.72% (217 genes) have a predicted targeted L1MdTf, versus 65.28% with a predicted non-targeted L1MdTf, which is a higher proportion than for upregulated or unchanged genes. This enrichment for predicted targeted L1MdTfs was mirrored in the LKF clone among downregulated genes (Fig. EV3A). To account for 3D genome organization beyond linear distance, we examined co-localisation of DEGs and predicted targeted L1MdTfs within topologically associating domains (TADs) (Bonev et al, 2017). Notably, 51.4% of TADs containing VPR-upregulated genes also harbored predicted targeted L1MdTfs, in contrast to 12.8% for downregulated genes. In the LKF clone, the opposite pattern was observed: TADs with downregulated genes were significantly enriched for predicted targeted L1MdTfs (Fig. EV3B). Altogether, our analyses show that misregulation of L1MdTf elements tends to impact nearby transcription and gene expression.

### Evidence for short-range *cis*-regulation by L1MdTf elements

**Figure EV4:**
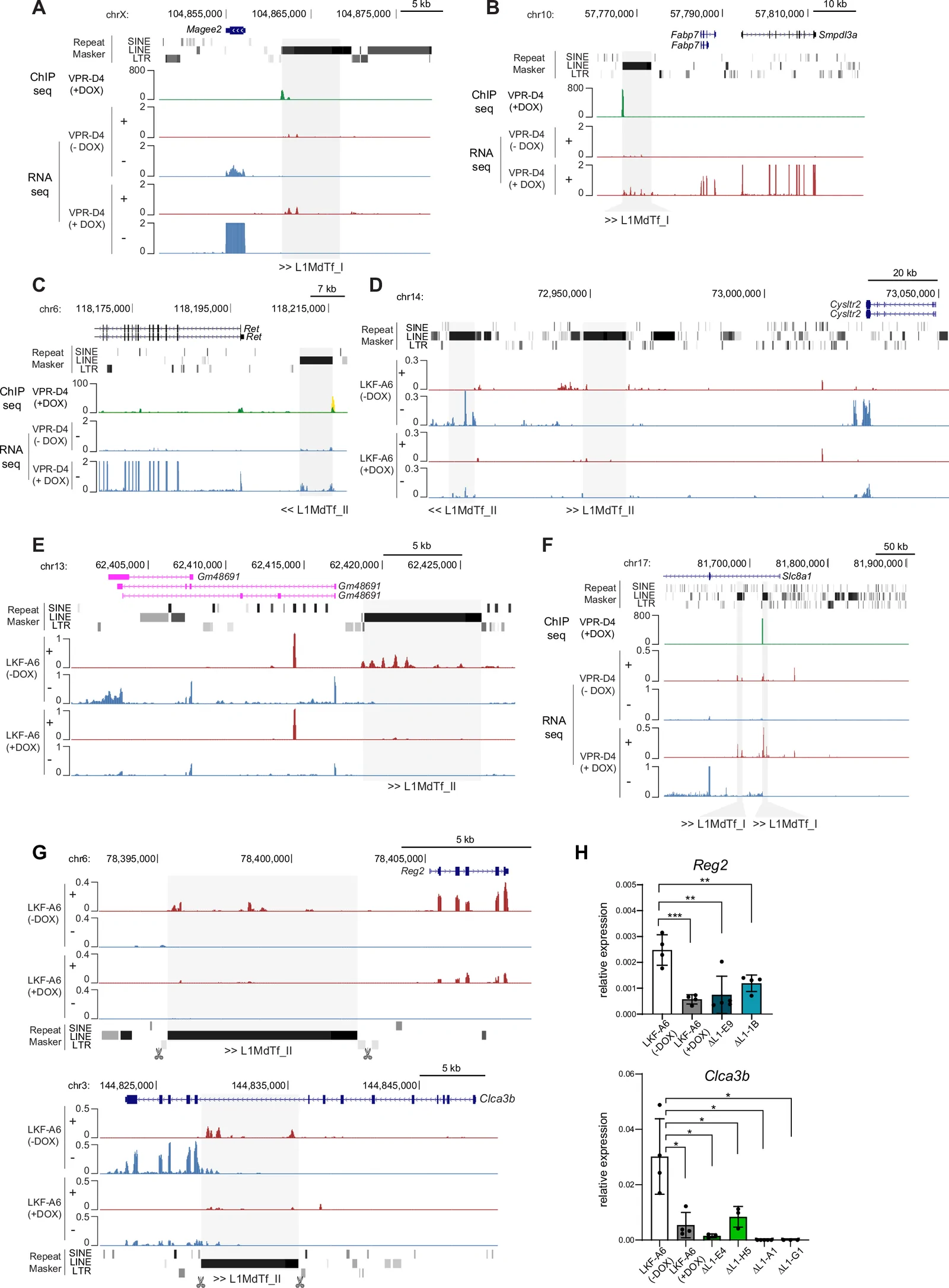
Evidence of *cis*-regulation of genes by proximal L1MdTf repeats. (**A**) *Magee2*, (**B**) *Fabp7/Smpdl3a* and (**C**) *Ret* gene vicinities showing increased expression upon L1MdTf activation. ChIP-seq for dCas9 (VPR-D4 clone, +DOX) profile is shown in green (uniquely mapped reads) and yellow (multimapped reads, not shown to scale). RNA-seq profiles by strand are shown in red (+ strand) and blue (− strand) for DOX-treated and untreated activator samples. Adjacent L1MdTf repeats with reciprocal expression change, and their orientation, are highlighted. (**D**) *Cysltr2* gene vicinity showing decreased expression upon L1MdTf LKF-mediated repression. Two adjacent L1MdTf elements, and their orientation, are highlighted. (**E**) *Gm48691* vicinity showing decreased expression upon adjacent L1MdTf repression as in (**D**). (**F**) *Slc8a1* vicinity showing increased expression upon L1MdTf activation. Intronic L1MdTfs with reciprocal changes and located in inverse orientation with respect to *Slc8a1* are highlighted. (**G**) *Reg2* (top) and *Clca3b* gene vicinities (bottom). RNA-seq profiles by strand (red (+ strand) and blue (− strand)) show downregulation of *Reg2* and *Clca3b* in LKF-A6 clone following DOX treatment. For both loci, L1MdTf elements are highlighted and the position of the sgRNAs used for CRISPR-mediated deletion are indicated with scissors. (**H**) qPCR analysis of *Reg2* (top) and *Clac3b* (bottom) in parental repressor clones LKF-A6 with or without 48 h of DOX treatment, compared to CRISPR-deleted (ΔL1) clones (2 independent clones for *Reg2*; 4 clones for *Clca3b*). Data are shown as mean ± s.e.m. (*n* = 3 to 5 biological replicates). Statistics: two-tailed unpaired *t* test (**H**): *P < 0.05, **P < 0.01, ***P < 0.001. Exact *P* values of significant differences shown from left to right: 0.0008, 0.0061, 0.0086 for *Reg2*; 0.0137, 0.0244, 0.0462, 0.0214, 0.0214 for *Clca3b*; null hypothesis: there is no difference in expression levels between −DOX and +DOX conditions, and between −DOX condition and ΔL1 clones.

Further exploration of the genome tracks for specific genes adjacent to misregulated L1s suggests that these elements may act as short-range *cis*-regulatory elements (e.g., *Magee2*, *Fabp7/Smpdl3a* and *Ret* genes in the VPR clones; *Cysltr2* and *Gm48691* in the repression system (Fig. EV4A–E)) or intronic alternative promoters (e.g., *Iqub*, *Pkhd1* and *Slc8a1* genes (Figs. 4A and EV4F)). Except for *Iqub* and *Gm48691* (lacking TAD annotation), DEGs and nearby L1MdTfs are consistently located in the same TAD. However, visualisation of chromatin loops from mESCs did not reveal any loop anchor overlapping these L1MdTfs, indicating that the regulation may be below the resolution threshold for detection or loop-independent for these genes.

An important question arising from these observations is whether nearby gene expression changes result from physical proximity of effector binding or from direct transcriptional perturbation of the loci. To test for L1MdTf-mediated *cis*-regulation, we used CRISPR-Cas9 to delete two L1MdTfs in the LKF-A6 repressor clone: an intergenic L1MdTf near *Reg2*, and an intronic L1MdTf within *Clca3b*, guided by sgRNAs flanking each element (Fig. EV4G). Deletion of either element recapitulated the effects of DOX-induced repression in the LKF-A6 clone, namely, reduced *Reg2* and *Clca3b* expression, supporting a direct *cis*-regulatory role for these elements (Fig. EV4H). Comparable deletion experiments were not feasible in the VPR clones, as this would remove the dCas9-targeted sequence. However, inspection of ChIP-seq and RNA-seq tracks revealed instances where dCas-VPR binding and L1 activation did not alter nearby gene expression, regardless of whether the element was intronic or intergenic. This indicates that proximity/binding alone is insufficient to drive changes in silent or expressed genes. Representative examples include upregulated genes (*Flrt2* and *Brinp2*) alongside unchanged ones (*Tyrp1*, *Lurap1l* and *Tmprss11d*) (Appendix Fig. S6A). Consistent with the distance analyses, these results show that L1 perturbation promotes, but does not generally dictate nearby gene misregulation.

Considering that the L1MdTf promoter can drive transcription in both sense and antisense direction (Fig. 3E; Appendix Fig. S3A,B), L1MdTfs could affect gene expression, regardless of their orientation. For instance, we identified a transcript that starts within the L1MdTf_II element located upstream of *Iqub* exon 7 by RT-PCR (Appendix Fig. S6B), leading to the production of a chimeric transcript and indicating that the antisense promoter activity of the L1 element can drive expression of an *Iqub* transcript from exon 7 onwards. Interestingly, L1-mediated gene expression of *Iqub* could also occur in cells in the absence of DOX since the first exons of the gene appear expressed at lower levels than exons located after the L1 element (Fig. 4A; Appendix Fig. S6B). As the L1MdTf promoter drives transcription in both directions (Fig. 3E; Appendix Fig. S3A,B), we also investigated the orientation of intronic DE L1MdTf elements with respect to the orientation of genes in which they are located, to determine the most prominent scenario. Out of the 53 upregulated and intronic L1MdTfs identified in the VPR clones, 31 were in the antisense orientation with respect to the upregulated genes. Similarly, 9 out of the 14 downregulated L1MdTfs overlapping downregulated genes in the LKF clone were also in antisense orientation. These proportions are similar to previous estimates of the orientation bias for intronic L1s (Nellaker et al, 2012). There is thus a higher proportion of antisense L1MdTf located within intronic sequences of genes (e.g., *Iqub* and *Pkhd1* in Fig. 4A; *Slc8a1* and *Clca3b* in Fig. EV4F,G; *Brinp2* in Appendix Fig. S6A), as expected given that repeats in the opposite orientation are less detrimental than sense-oriented ones (Zhang et al, 2011).

### Identification of repeats and genes with skewed allelic expression

Taking advantage of the fact that the PGK-G10 cell line used to generate the L1MdTf repressor and activator lines is a hybrid F1 line with PGK and 129 genotypes (Marks et al, 2009; Penny et al, 1996), we sought to determine if the transcriptional changes observed for L1MdTfs occur specifically for one of the alleles. This could suggest that the associated repeats are polymorphic and present on only one allele. To assess this, we first identified repeats with skewed allelic expression. Considering all repeat families, more than 35 elements showed skewed expression towards each of the alleles in the two effector cell lines (Appendix Fig. S6C). The specific numbers are lower for the activator as only the repeats with significant differences from biallelic expression common between VPR-B4 and VPR-D4 are considered. No L1MdTfs were found among the repeats with skewed allelic expression, which is consistent with the fact that young L1s have low levels of sequence diversity (Teissandier et al, 2019). Actually, very few L1MdTfs (<10 elements in each of the comparisons) have uniquely mapped reads that could be assigned to one of the alleles. Nevertheless, as an alternative strategy to identify L1MdTfs affecting gene expression in *cis* on one allele only, we next identified genes with skewed expression and checked if they corresponded to DEGs upon perturbation of L1MdTfs. Overall, more genes (268 for the repressor and 210 for the activator) than repeats (135 for the repressor and 82 for the activator) showing skewed allelic expression were identified for the two alleles (Appendix Fig. S6D). However, only 14 and 1 were DEGs in the L1MdTf activator and repressor clone analyses, respectively. Two examples of such genes are *4930579G24Rik* and *Cysltr2*, which are located proximally to L1MdTfs, 1336 bp and 88,908 bp away, and show skewed expression for the PGK or 129 allele, respectively (Appendix Fig. S6E).

In this analysis, we assigned RNA-seq reads to alleles using only those SNPs that can be annotated with high confidence as PGK or 129 based on public annotations (see methods). Therefore, future work to complement the assignment of all heterozygous SNPs detected, usage of hybrid lines with different genetic background or long-read RNA-seq will improve the detection of L1MdTfs with allele-specific functions.

### Misregulated genes by L1MdTf effectors are enriched at repressed regions and are part of the neuronal network

**Figure 6:**
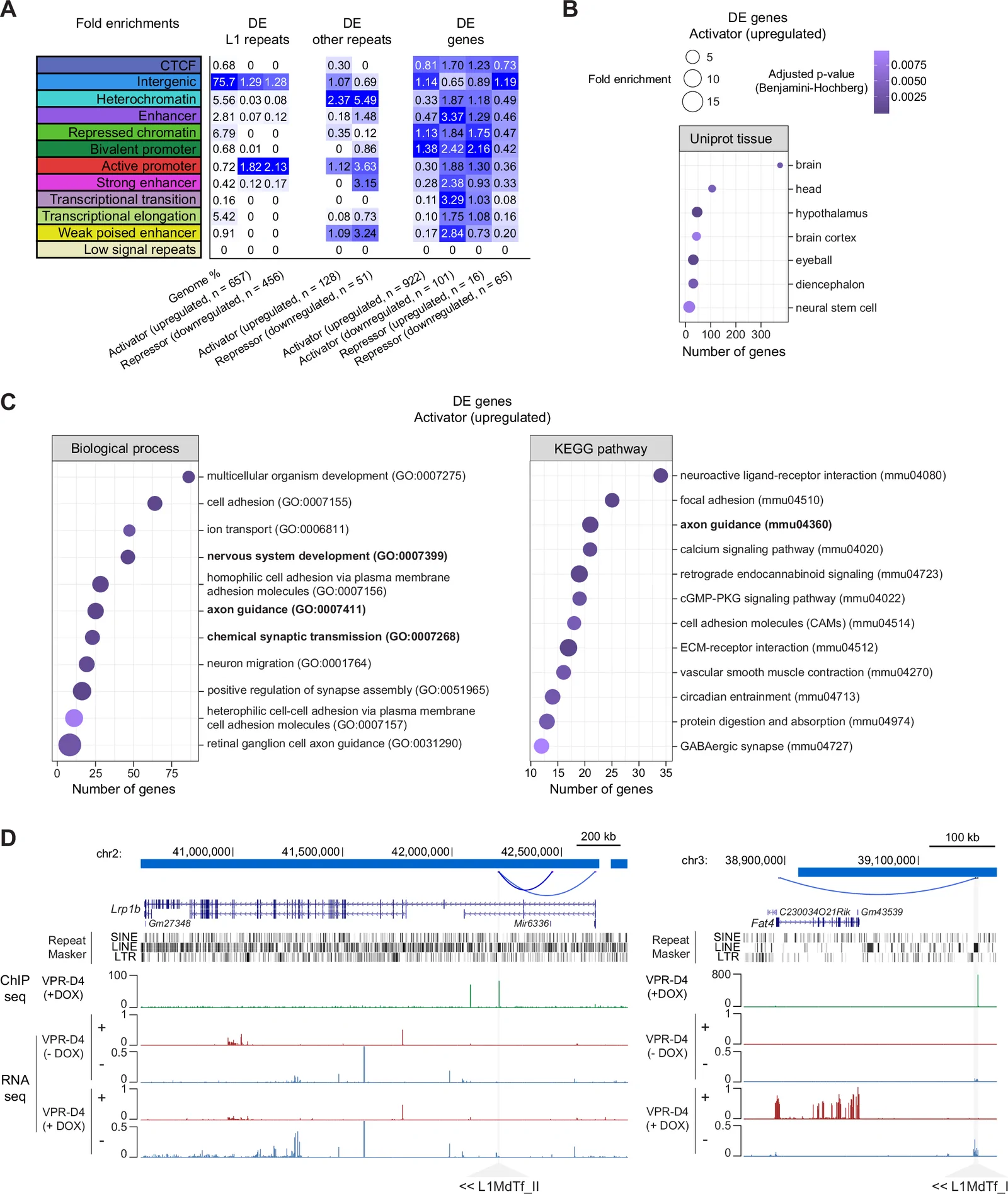
Chromatin state and GO enrichment analyses for DEGs and DERs. (**A**) Heat map showing fold enrichment for each chromatin state of DE repeats and genes classified based on expression fold change direction. Increased color darkness represents increased enrichment. (**B**) Expression enrichment of common upregulated genes in activator clones relative to Uniprot tissue terms. The number of genes by term is shown on the *x* axis, circle size indicates fold enrichment and darker colors indicate higher significance based on Fisher’s Exact Test; null hypothesis: the proportion of observed genes in the analyzed list is not different from the background. (**C**) GO enrichment analysis of biological process terms (left) and KEGG pathways (right) for the common upregulated genes in activator clones. Bold labels correspond to terms found in GO analysis of L1MdTf-containing upregulated genes. Plot annotation as described for (**B**). (**D**) Examples of chromatin interactions between the promoters of upregulated genes following L1MdTf activation and observed dCas9-bound L1MdTfs. Blue rectangles and arcs represent mESC TADs and loops, respectively. ChIP-seq for dCas9 (VPR-D4 clone, +DOX) profile is shown in green (uniquely mapped reads). RNA-seq profiles by strand are shown in red (+ strand) and blue (− strand) for the DOX-treated and untreated samples. Observed targeted L1MdTf repeats and their orientation are highlighted in grey. Source data are available online for this figure.

To analyse further the role that young L1s may play in gene regulation, we investigated whether DERs and DEGs found in the LKF and VPR clones (Dataset EV5) are located in specific genomic or chromatin environments using published ESC ChromHMM state annotations. These annotations classify mouse genomic regions in 12 states based on 7 chromatin marks (H3K27me3, H3K36me3, H3K27ac, H3K4me1, H3K4me3, H3K9ac, H3K9me3) and binding profiles of 3 transcription factors (CTCF, Nanog, Oct4) (Pintacuda et al, 2017). First, we looked at L1s and found that VPR-upregulated and LKF-downregulated L1 elements tend to be associated with chromatin features of active promoter and intergenic regions (Fig. 6A; Dataset EV7). Enrichment at active promoters was consistent with our observation that misregulated L1s could act as promoters, controlling nearby gene expression. Of note, VPR-upregulated L1s showed general enrichment in such regions independently of their expression fold change, as reflected by the common results obtained for the repeat clusters 1 and 2 (Fig. 3G; Appendix Fig. S7A). We next analysed the chromatin features associated with non-L1 DERs. We found that VPR-upregulated and LKF-downregulated non-L1 repeats colocalize mainly with heterochromatin, but also with promoter or poised enhancer regions (Fig. 6A; Dataset EV7). VPR-downregulated repeats on the other hand, corresponding mainly to repeat cluster 3 and ERVs elements (Fig. 3G), are enriched with chromatin marks found at strong enhancers (Appendix Fig. S7A; Dataset EV7). In the case of DEGs, we observed that weakly and strongly VPR-upregulated DEGs are enriched in bivalent promoter chromatin domains, as well as repressed chromatin and intergenic regions (Fig. 6A; Appendix Fig. S7A; Dataset EV7). Genes associated with bivalent promoters or located in repressed/intergenic chromatin environments have generally low expression levels or are silenced in ESCs (Bernstein et al, 2006). This is therefore in agreement with our observations that a fraction of VPR-upregulated genes, found in cluster 2 and 3 DEGs, tend to be weakly expressed in ESCs (Fig. 4E). Some of these genes may be silenced or poised for subsequent activation later during ESC differentiation. On the other hand, VPR-downregulated genes (cluster 4 (Fig. 4E)) colocalize with enhancer chromatin states, as observed for downregulated non-L1 repeats (Fig. 6A; Appendix Fig. S7A; Dataset EV7). Finally, we mainly observed LKF downregulated genes at intergenic regions, possibly due to the lower number of regions considered (*n *= 65). Overall, our observations highlight that most genes misregulated following L1 perturbation are found within repressive or silenced domains in ESCs.

We next performed a gene ontology (GO) and KEGG pathway enrichment analysis to identify specific terms or pathways enriched in our differentially expressed gene sets. Given that the number of DEGs was too low in the repression system (*n* = 81), this analysis was only performed for upregulated (*n* = 923) and downregulated (*n* = 101) genes in the activation system (Fig. 6B,C; Appendix Fig. S7B–D). Interestingly, we found that metabolism-related terms are enriched in the downregulated gene set (Appendix Fig. S7B; Dataset EV8). This could be related to the increased cell death observed following DOX induction and L1MdTf activation. For the set of upregulated genes, we first observed enrichment for genes specifically expressed in various brain regions and in neural stem cells (Fig. 6B). Biological process and KEGG pathway analysis revealed a predominance of neuronal function and development-related terms (Fig. 6C; Dataset EV8). Interestingly, analysis of cluster 2 and cluster 3 DEGs (Fig. 4E; Dataset EV8) revealed an over-representation of genes expressed at the cell surface and related to synaptic function (Appendix Fig. S7C,D). Most of these genes are not expressed or have low expression levels in ESCs compared to other upregulated genes that are not part of these processes and pathways (Appendix Fig. S7E). However, length-adjusted GO analyses eliminated the initially observed GO enrichments, likely due to neuronal-related genes being more strongly penalized given their larger average length (Zylka et al, 2015). To address whether the original enrichment of neuronal terms could be related to the presence of intronic L1MdTfs, we calculated the percentage of genes containing FL-L1MdTf elements (>6 kb) for each GO term and compared the average percentage for neuronal-related terms to that of all other GO terms. This analysis revealed that the percentage of genes containing FL-L1MdTf elements in neuronal GO terms is significantly higher than in other GO categories (Appendix Fig. S7F). These findings indicate that longer neuronal genes are more likely to harbor L1 elements and may therefore be more vulnerable to L1 misregulation, as evidenced by their overrepresentation among VPR-upregulated genes and their proximity to predicted targeted elements. This L1-specific vulnerability is supported by L1-focused GO analysis of VPR-upregulated genes containing FL-L1MdTfs, which recovered five significant neuronal terms (bold labels in Fig. 6B,C). Interestingly, all chromatin loops detected between predicted targeted L1MdTfs and DEGs involve genes associated with neuronal functions (Fig. 6D; Dataset EV9). Given that L1 activity is reportedly higher in neural stem cells and neurons, our data suggest that the expression of these neuronal genes may be correlated with higher L1 expression, consistent with emerging roles of young L1s in neural development.

## Discussion

In this study, we used engineered effectors to perturb the expression of full-length L1 elements and test their regulatory potential in mESCs, focusing on one of the three most active L1Md subfamilies in the mouse genome, independently of their coding nature and retrotransposition competence. Our data show that perturbing FL-L1MdTf expression in ESCs has an impact on the expression of genes or repeats located in *cis* in the neighboring environment of these elements. Remarkably, L1MdTf activation is associated with a much higher number of misregulated genes than L1MdTf repression. Moreover, our analysis revealed that most of the affected genes, upon L1MdTf activation, are silenced or lowly expressed genes in ESCs, and a significant fraction of these have neuronal-related functions. Short read RNA-seq was used in this study to quantify the expression of both genes and TEs. Although this approach inevitably misses information, particularly for evolutionary young TEs with low levels of sequence diversity, the expression of individual TE loci could still be confidently detected, consistent with prior studies using similar methods (Adami et al, 2025; He et al, 2019; Pal et al, 2023). Similarly, identification of dCas9 binding sites relied on short-read data processed either to retain only uniquely mapped reads, for a high confidence set of observed binding sites, or to retain multimapping reads, to broaden the search for off-target sites. Because L1MdTf copies are highly similar, ChIP-seq signal at its promoters is difficult to resolve unambiguously. Therefore, the multimapping strategy improves sensitivity for detecting binding at repetitive loci, but may assign a read to one of several equivalent loci rather than to the true locus from which it originated.

Previous analyses addressing the effects of interfering with mouse L1 transcriptional activity using TALE technology or ASOs for L1 RNA knockdown reported an impact on chromatin accessibility. However, in both studies, the authors found no evidence of misregulation of genes with an L1 element located within or in close proximity (Jachowicz et al, 2017; Percharde et al, 2018). The differences with our study may result from the technology used to interfere with L1 expression, the identity of targeted L1 elements, or the model used. TALE-mediated activation or repression of FL-L1 elements, from various subfamilies, was performed in mouse preimplantation embryos. The authors found that perturbing L1 transcriptional activity affects global chromatin condensation, with minor consequences on gene expression, including genes located near L1 elements (Jachowicz et al, 2017). ASOs targeting the coding region of L1s were used in mESCs and preimplantation embryos to induce the depletion of L1 transcripts produced from any L1 subfamily (Percharde et al, 2018). Unlike CRISPR or TALE-based methods that interfere directly with transcription, this approach enables the analysis of exclusively *trans*-regulation effects mediated by L1 RNA or eventually L1-encoded proteins. One example of *trans*-regulation is the finding that L1 RNA functions as a nuclear RNA scaffold in mESCs repressing essentially the 2-cell embryo transcription program. Nevertheless, expression of genes located proximally to L1s was not affected (Percharde et al, 2018).

The engineered effectors used in our study affect transcription and hence lead to a reduction or increase of global L1 RNA and protein levels, as shown by qPCR, RNA FISH, and Western blot analysis. This strategy allows us, therefore, to test both the *cis*- and *trans*-effects mediated by L1-encoded products. We did not observe a loss of ESC self-renewal nor activation of the 2-cell gene expression program following L1 repression, as previously reported following ASO-mediated L1 RNA depletion (Lu et al, 2020; Percharde et al, 2018; Wei et al, 2022). The extent of total L1 RNA levels depletion might explain the differences observed, given that in this study we are targeting only one active L1 subfamily. We noticed, however, that one of the two analyzed VPR clones (VPR-D4) showed significant upregulation of some genes belonging to the 2-cell program (e.g., the cluster of *Zscan4* genes on chr7). This was not observed in the other clone (VPR-B4) or to a much lesser extent (Dataset EV5), which may reflect cell-to-cell differences existing in the population prior to subcloning. It should be noted that these genes do not lie near misregulated FL-L1MdTfs. These observations suggest a *trans* effect, whereby perturbation of L1 RNA levels, whether increased (our study) or decreased (Lu et al, 2020; Percharde et al, 2018; Wei et al, 2022), has an impact on chromatin organization with consequences on gene expression, as previously suggested (Lu et al, 2020; Percharde et al, 2018; Wei et al, 2022). Genes from the 2-cell stage expression program appear to be particularly sensitive to changes in chromatin structure. We hypothesize that other gene clusters found to be affected in both VPR clones, such as GABA receptor subunit, Keratin, Mage, *Rhox* genes, may also be particularly sensitive to changes in chromatin organization following perturbation of L1 RNA levels.

In our study, we found that a significant proportion of genes or repeats showing differential expression following L1MdTf expression perturbation, are located within a < 3 Mb distance from misregulated FL-L1MdTfs. This finding suggests that these elements may act as enhancers when located in intergenic regions (Fig. EV4A–E,G) or alternative promoters when located in introns (Figs. 4A and EV4F,G). This is supported by our ChromHMM state analysis, showing that a proportion of misregulated L1MdTfs tends to colocalize with chromatin signatures of active promoter regions. Interestingly, the 5’UTR regions of some active young L1 elements are enriched with H4K16ac and H3K122ac, chromatin features associated with active enhancers in human ESCs (Pal et al, 2023; Pradeepa et al, 2016; Taylor et al, 2013). Downregulation or deletion of acetylation positive-L1s, but not of acetylation negative-L1s, is associated with downregulation of genes in *cis*. This indicates that these elements, which can be found within introns or in intergenic regions, can function as *cis*-regulatory elements, a scenario that echoes our observations. It remains to be investigated whether specific chromatin signatures distinguish those active mouse L1s elements that were found to influence nearby gene expression in mESCs from those that do not. Moreover, CRISPR-mediated L1 perturbation studies in human cells reported that transcription of evolutionary young L1 elements is associated with long range gene activation (Li et al, 2024), as well as facilitating expression of neighboring genes, in agreement with our observations (Adami et al, 2025; Tong and Sun, 2023). Together, our study and these recent findings collectively highlight a conserved role for a subset of young and active L1s, in both mouse and human cells, as *cis*-acting regulatory elements.

Importantly, we observed that both sense or antisense transcription activity of L1MdTfs could modify nearby gene transcription or lead to the production of chimeric transcripts. This was previously reported for L1 elements in human cells, where the antisense promoter of FL-L1s has been well characterized (Speek, 2001). The latter was shown to act as an alternative promoter for more than 40 protein-coding genes (Matlik et al, 2006; Speek, 2001). Interestingly, both the canonical sense and antisense L1 promoters appear expressed in a tissue-restricted fashion in human tissues, suggesting that L1 activity might broaden the transcriptional potential for a given gene (Faulkner et al, 2009; Matlik et al, 2006). Our study uncovers that this could extend to mouse cells, especially considering the transcriptional dynamics of the mouse L1 promoter during early development, in neuronal lineages and certain disease contexts. Furthermore, our allelic expression analysis illustrates how polymorphic TE insertions could contribute to strain-specific variation in gene expression, as highlighted in a genomic analysis of 20 laboratory mouse strains (Ferraj et al, 2023). Our results suggest that some L1MdTfs insertions analysed in this study are potentially polymorphic in the ESC line used and can impact gene expression in an allele-specific manner. Of note, the reported number of genes showing skewed allelic expression is likely underestimated due to the limited number of high confidence SNPs used.

In terms of previously identified putative targets of TE-mediated gene regulation, the case of the *Fabp7* gene is a noteworthy example*. FABP7* ectopic expression was shown to be controlled by an LTR2 promoter located 30 kb upstream in a subset of human diffuse large B cell lymphomas, resulting in the expression of a TE-gene chimeric transcript (Lock et al, 2014). In our study, we observed that *Fabp7* and the neighbouring gene *Smpdl3a*, which are not expressed in ESCs, are upregulated following VPR induction. This is correlated with the upregulation of an L1MdTf_I element located 10 kb upstream and transcribed in the same direction (Fig. EV4B). We speculate that the L1MdTf element may act as an enhancer controlling the expression of both genes located downstream rather than the production of chimeric transcripts. This could be yet another example where different species have independently co-opted specific TEs with regulatory potential to regulate the same gene (Chuong et al, 2017).

Our findings indicate that the transcriptional potential of young L1MdTf elements can be repurposed by the host genome in specific contexts or regions to regulate nearby genes in *cis*. This may be particularly relevant in developmental or tissue-specific contexts, where L1 activity is higher. For instance, our GO analysis revealed that many VPR-activated genes have brain or neuronal-related functions. These genes are largely silenced or lowly expressed in ESCs, only becoming active later during differentiation. Considering that L1 activity is reported to be higher in neuronal cells, there may be a coordinated expression between L1s and VPR-activated genes in neurons. This raises the possibility that some L1 elements have evolved to control neuronal gene networks. Such effects could be mediated not only through local *cis*-acting mechanisms as we suggest here, but also via *trans*-regulatory functions by L1 RNAs, previously shown to impact chromatin organisation in mESCs and during neuronal differentiation (Mangoni et al, 2023; Percharde et al, 2018; Toda et al, 2024). L1 transcription could particularly impact genomic regions where young L1 elements are more abundant, such as the X chromosome. Importantly, because L1s can be reactivated in diseases, including cancer or neurodegenerative disorders (Burns, 2017; Ravel-Godreuil et al, 2021), their misregulation may represent an important route by which TEs drive pathological states. Future work using similar strategies could determine when and how TE expression participates in disease onset or progression.

## Methods

**Table:** Reagents and tools table

Reagent/resource	Reference or source	Identifier or catalog number
**Experimental models**		
PGK12.1 (ESC line)	Norris et al, 1994	N/A
PGK-G10 clone	This study	“Methods” section
**Recombinant DNA**		
pEN111 (*Rosa26*-donor)	This study	Gift from ENora
pEN111-TRE3G-LKF-IRES-ZsGreen1-rtta3G_*Rosa26*-donor	This study	“Methods”/Dataset EV10
pEN111-TRE3G-AKF-IRES-ZsGreen1-rtta3G_*Rosa26*-donor	This study	“Methods” section
pEN366 (*Tigre*-donor)	Addgene	#156432
pEN366-TRE3G-dCas9-VPR-rtta3G_*Tigre*-donor	This study	“Methods” section
pX330-EN1201 (*Tigre* gRNA)	Addgene	#92144
pX335-EN479 (*Rosa26* gRNA)	This study	Gift from E Nora/Dataset EV10
pX335-EN481 (*Rosa26* gRNA)	This study	Gift from E Nora/Dataset EV10
pLKO1-blast-U6-sgRNA-BfuA1-stuffer	Holoch et al, 2021	N/A
pLKO1-blast-U6_dual_sgTf-mono2-3	This study	Methods/Dataset EV10
pX458	Addgene	#48138
pX458-mCherry	Casanova et al, 2019	N/A
pX458-L1-Clca3b-up	This study	“Methods”/Dataset EV10
pX458-mCherry-L1-Clca3b-dn	This study	“Methods”/Dataset EV10
pX458-L1-Reg2-up	This study	“Methods”/Dataset EV10
pX458-mCherry-L1-Reg2-dn	This study	“Methods”/Dataset EV10
**Antibodies**		
Rabbit anti-ORF1p	Abcam	ab216324
Rabbit anti-LaminB1	Abcam	ab16048
Mouse anti-phospho-Histone H2A.X (Ser139)	Sigma	05-636
Rabbit anti-Cas9	Recombinant Antibodies Platform - Institut Curie	A-P-R#56
Mouse anti-Cas9	Active Motif	AB_2793760
Normal Mouse IgG	Merck Millipore	12-371
Goat anti-rabbit IgG (H + L) -HRP conjugate	Bio-Rad	170-6515
Goat anti-mouse IgG (H + L) -HRP conjugate	Bio-Rad	170-6516
Goat anti-rabbit IgG (H + L) cross-adsorbed Cy3	Bethyl Laboratories	A120-201C3
Goat anti-mouse IgG (H + L) DyLight 488	Bethyl Laboratories	A90-116D2
**Oligonucleotides and other sequence-based reagents**		
Guide RNAs	This study	Dataset EV10
Genotyping primers	This study	Dataset EV10
RT-qPCR primers	This study	Dataset EV11
**Chemicals, enzymes and other reagents**		
Leukemia inhibitory factor (LIF)	Millipore	ESG1107
Trizol reagent	Invitrogen	15596018
RNase-free DNase set	Qiagen	79254
SuperScript III reverse transcriptase	Invitrogen	18080093
PowerUP SybrGreen PCR master mix	Applied Biosystems	A25742
GoTaq DNA polymerase	Promega	M3005
Puromycin	InvivoGen	ant-pr-1
Blasticidin	InvivoGen	ant-bl-05
Doxycycline	Sigma	D9891-1G
ECL Western blotting detection reagents	Amersham	RPN2106
Trypan blue	Sigma	T6146
Propidium iodide	Merck Millipore	P4864-10mL
RNase A	Roche	10109142001
Proteinase K	NZYtech	MB01902
Dynabeads Protein G	Invitrogen	10004D
Protease inhibitor mini tablets, EDTA-free	Thermo Scientific	A32955
**Software**		
ImageJ	https://imagej.net/	N/A
GraphPad Prism v8.4.3	https://www.graphpad.com/	N/A
Bowtie v1.2.3	Langmead et al, 2009	N/A
RIsearch2 v2.1	Alkan et al, 2017	N/A
CRISPRoff v1.1.2	Alkan et al, 2018	N/A
bedtools v2.29.0 & v2.31.1	Quinlan and Hall, 2010	N/A
Trim_galore v0.6.10	https://github.com/FelixKrueger/TrimGalore	N/A
Bowtie2 v2.5.4	Langmead and Salzberg, 2012	N/A
samtools view v1.9 & v1.20	Li et al, 2009	N/A
picard tools v2.20.8 & v3.3.0	https://broadinstitute.github.io/picard/	N/A
MACS v2.2.9.1	Zhang et al, 2008	N/A
BWA mem v0.7.17-r1188	Li et al, 2009	N/A
bcftools v1.9	Li et al, 2014	N/A
freebayes v1.3.2	Garrison and Marth, 2012	N/A
vcffilter v1.0.0	Garrison et al, 2022	N/A
STAR v2.7.2b	Dobin et al, 2013	N/A
SNPsplit v0.3.4	Krueger and Andrews, 2016	N/A
featureCounts v2.0.1	Liao et al, 2014	N/A
bamCoverage v3.1.3	Ramirez et al, 2016	N/A
DESeq2 v1.26.0	Love et al, 2014	N/A
EnhancedVolcano	https://github.com/kevinblighe/EnhancedVolcano	N/A
deepTools v3.4.3	Ramirez et al, 2016	N/A
R	R Core Team, 2023	N/A
corrplot v0.88	https://github.com/taiyun/corrplot	N/A
Mustache	Roayaei Ardakany et al, 2020	N/A
ChromHMM OverlapEnrichment v1.23	Ernst and Kellis, 2017	N/A
DAVID v6.8	Sherman et al, 2022	N/A
GOseq	Young et al, 2010	N/A
GOglm	https://github.com/gu-mi/GOglm/tree/master	N/A
**Other**		
P3 Primary Cell 4D-Nucleofector X Kit	Lonza	V4XP-3024
QIAprep Spin Miniprep Kit	Qiagen	27104
RNeasy mini kit	Qiagen	74104
MinElute PCR Purification Kit	Qiagen	28004
ViiA 7 Real-Time PCR System	Applied Biosystems	N/A
NovoCyte 2000R benchtop flow cytometer	Agilent Technologies	N/A
BD Accuri C6 Plus flow cytometer instrument	BD Biosciences	N/A
NovaSeq6000	Illumina	N/A
NovaSeq X plus	Illumina	N/A
HiSeq 2500	Illumina	N/A

### Genomic engineering of ESCs

#### ES cell lines

All cell lines used in this study are derived from feeder-independent PGK12.1 mouse female embryonic stem cells (ESCs) (Norris et al, 1994; Penny et al, 1996) and were maintained on gelatin-coated flasks in serum-containing ES cell medium (DMEM supplemented with 15% ESC-grade foetal bovine serum (FBS), 1000 U/ml leukemia inhibitory factor (LIF, Millipore) and 0.1 mM B-mercaptoethanol (Sigma)) in a humidified atmosphere at 37 °C with 8% CO_2_. PGK12.1 is a hybrid cell line, which originates from mouse crossbreeds of two inbred strains (129 and C3H/He; both Mus musculus domesticus) with the distant strain PGK-1a/Ws (Mus musculus musculus) (Marks et al, 2009; Penny et al, 1996).

The PGK-G10 line employed in this study for genome engineering (derived from PGK12.1) exhibits a heterozygous deletion spanning a 5 kb segment encompassing the Xist promoter on chromosome X, ranging from the Xist TSS to 5 kb upstream (unpublished).

#### Plasmids

Knock-ins of the PGK-G10 ESC line were generated via CRISPR/Cas9 mediated homologous recombination. Donor plasmids used for knock-ins at the *Rosa26* (pEN111) and *Tigre* locus (pEN366, Addgene #156432), as well as the *Rosa26*- and *Tigre*-specific sgRNA-encoding plasmids (pX335-EN479, pX335-EN481 and pX330-EN1201 (Addgene #92144)) were all provided by E Nora (UCSF).

The L1MdTf binding-zinc fingers were designed to bind a 20 bp sequence found within the consensus monomer sequence from the 5’UTR region of L1MdTf elements (Fig. EV1A). A DNA fragment containing an array of 6 L1MdTf zinc-fingers, a KRAB repressor, and a FLAG tag sequences (LKF), as well as a DNA fragment comprising the mutated version (AKF), were then synthesised by GenScript and initially cloned into pLVX-IRES-ZsGreen1 lentiviral vector (Takara Bio) in frame with an IRES-ZsGreen1 sequence. The LKF/AKF-IRES-ZsGreen1 fragments (2138 bp) were amplified by PCR and cloned at the ClaI site in the *Rosa26*-targeting vector pEN111 by Gibson assembly (New England Biolabs). The resulting vectors obtained (pEN111-TRE3G-LKF/AKF-IRES-ZsGreen1-rtta3G_Rosa26-donor) were amplified and sequenced to ensure correct sequence and cloning.

The TRE3G-dCas9-VPR fragment was obtained by digestion of the PB-TRE-dCas9-VPR vector (Addgene #63800) with PmeI and PspXI. The fragment was subsequently cloned by DNA ligation in the donor vector pEN366 (Addgene #156432), after removal of the TRE3G-CTCF-mRuby2 fragment by digestion using the same restriction enzymes, PmeI and PspXI. The resulting vector obtained (pEN366-TRE3G-dCas9-VPR-rtta3G_Tigre-donor) was amplified and sequenced to ensure correct cloning.

Guide RNAs for L1MdTf 5’UTR were designed using the 212 bp consensus sequence of L1MdTf-monomers obtained from Goodier et al (Goodier et al, 2001) and the CRISPOR (Concordet and Haeussler, 2018) online tool. Two sgRNAs (sgTf-mono2 and sgTf-mono3) binding the monomer at two independent locations (Fig. EV1A) and highly represented across L1MdTf elements in the genome were selected. The pLKO1-blast-U6-sgRNA-BfuA1-stuffer was used for the dual cloning of these two sgRNAs in tandem following the strategy described by Holoch et al (Holoch et al, 2021). Guide RNAs overlapping unique sequences flanking selected L1MdTf for deletion were designed using CRISPOR and respectively cloned under a U6 promoter into pX458 (Addgene #48138) and pX458-mCherry (Casanova et al, 2019).

The resulting constructs, pLKO1-blast-U6_dual_sgTf-mono2-3, pX458-L1-Clca3b-up, pX458-mCherry-L1-Clca3b-dn, pX458-L1-Reg2-up, pX458-mCherry-L1-Reg2-dn were amplified and sequenced to ensure correct cloning.

#### Transfections of ESCs and clone isolation

Transfections of DNA constructs in ESCs were performed using the P3 Primary Cell 4D-Nucleofector X Kit (V4XP-3024) and the Amaxa 4D Nucleofector system (Lonza). For each nucleofection, 5 million cells were resuspended in the nucleofection mix (prepared according to manufacturer’s instructions) and electroporated with 2.5 μg each of non-linearized vectors. Nucleofected cells were then serially diluted and plated on 10-cm dishes for colony picking and selection.

For knock-ins of the LKF or AKF transgenes at *Rosa26*, the PGK-G10 cell line was nucleofected with pEN111-TRE3G-LKF-IRES-ZsGreen1-rtta3G_Rosa26-donor (or AKF), pX335-EN479 and pX335-EN481. Puromycin selection (1 μg/ml) was started 2 days after transfection and kept for 8–10 days. Single colonies were then picked into 96-well plates. Genomic DNA was isolated directly in 96-well plates for PCR-based screening of knock-ins using primers overlapping the inserted construct and the surrounding *Rosa26* region. Twelve successfully knocked-in clones were selected, amplified, and analysed for induction of expression of the LKF/AKF transgene and the impact on repression of L1MdTf elements upon doxycycline (DOX) (1 μg/ml) treatment for 48 h.

For knock-in of the dCas9-VPR transgene at *Tigre*, the PGK-G10 cell line was nucleofected with pEN366-TRE3G-dCas9-VPR-rtta3G_Tigre-donor and pX330-EN1201. Puromycin selection (1 μg/ml) was started 2 days after transfection and kept for 8–10 days. Single colonies were then picked into 96-well plates. Genomic DNA was isolated directly in 96-well plates for PCR-based screening of knock-ins using primers overlapping the inserted construct and the surrounding *Tigre* region. Four successfully knocked-in clones were selected, amplified and analysed for induction of expression of dCas9-VPR upon DOX treatment (1 μg/ml) for 48 h. One dCas9-VPR-expressing clone (VPR-B7) was selected for transfection with pLKO1-blast-U6_dual_sgTf-mono2-3. Transfected cells were selected with blasticidin (10 μg/ml) 24 h after transfection for 10–12 days. Cells were first analysed in bulk to test sgRNAs efficiency with or without DOX (1 μg/ml) by RT-qPCR. Cells were then seeded in 10-cm dishes to ensure optimal density for colony-picking in 96-well plates. Twelve clones were expanded and screened in six-well plates in the presence or absence of DOX by RT-qPCR to assess L1MdTf upregulation efficiency.

For single L1MdTf deletion, ES cells (LKF-A6 clone) were co-transfected with 1 μg of each construct containing upstream and downstream sgRNAs. Double-positive cells (GFP + /mCherry + ) were sorted by fluorescence-activated cell sorting (FACSAria III, BD BioSciences) 48 h after transfection and plated onto 0.1% gelatin in ES medium. Individual colonies were manually picked into 96 wells ~10 days after transfection. Deletion events were screened by endpoint PCR. Sequences of all sgRNAs and genotyping primers used for each engineered locus can be found in Dataset EV10.

### RNA extraction, reverse transcription, qPCR and PCR

For RNA extraction, cells grown in six-well plates in the presence or absence of DOX (1 μg/ml) for 48 h were briefly washed in 1× PBS and harvested directly in Trizol (Invitrogen). Total RNA was extracted from Trizol with chloroform by phase separation. The aqueous phase was then mixed with an equal volume of 70% ethanol and transferred to a silica column from the RNeasy Mini kit (Qiagen). Total RNA was then purified according to the instructions of the manufacturer, including on-column DNAse I digestion (Qiagen).

Total RNA (500 ng to 1 µg) was reverse-transcribed into cDNA using random primers and SuperScript III reverse transcriptase (Invitrogen) for 1 h at 50 °C. Quantitative PCR (qPCR) was performed using Power SybrGreen PCR master mix on a ViiA 7 real-time PCR system using standard settings (Applied Biosystems). Each RNA sample was analysed in three technical replicates. Expression of genes was normalised to two housekeeping genes (*Bactin*, *Rrm2*). Statistical significance across biological replicates was assessed using a two-tailed paired *t* test. Endpoint PCR was performed on cDNA using the GoTaq DNA polymerase (Promega). All primer sequences can be found in Dataset EV11.

### Western blot analysis

Whole-cell extracts were prepared using RIPA buffer supplemented with protease inhibitors, and proteins were separated by SDS-PAGE (8%) and blotted onto a nitrocellulose membrane using the dry transfer iBlot System (Thermo Fisher Scientific). Western blotting was performed using PBS-Tween 0.05%. Primary antibodies to ORF1p and LaminB1 were from Abcam (ab216324 (dilution 1:1000) and ab16048 (1:2000)), and to γH2AX from Sigma (05-636 (dilution 1:1000)). Primary antibody for Cas9 was from the Recombinant Antibodies Platform at Institut Curie (A-P-R#56, 1:2500). Secondary antibody anti-rabbit-HRP (Bio-Rad, 170-6515) and anti-mouse HRP (Bio-Rad, 170-6516) were used for ECL detection in accordance with the manufacturer’s recommendations using an Amersham imager 680.

### Flow cytometry

Cells grown in 6-well plates in the presence or absence of DOX (1 μg/ml) for 48 h were trypsinized, harvested in medium and then washed in 1× PBS before fluorescence analysis. ZsGreen1 fluorescence was analysed using the NovoCyte 2000R benchtop flow cytometer (Agilent). Fluorescence was acquired and analysed using the NovoExpress software following the manufacturer’s instructions. Briefly, the percentage of ZsGreen1-positive cells was determined upon definition of three gates: (i) FSC-H vs SSC-H, to isolate cells from debris, (ii) FSC-H versus FSC-A, to isolate single cells and (iii) FSC-H versus FITC-A for detection of ZsGreen1-positive population.

### Immunofluorescence

ES cells were grown on coverslips for 48 h in the presence or absence of DOX (1 μg/ml). Cells were rinsed in 1× PBS, fixed for 10 min in 3.7% paraformaldehyde, rinsed in 1× PBS and permeabilised for 5 min in 1× PBS/0.5% Triton on ice. Cells were then blocked for 30 min at room temperature in 1× PBS/0.2% fish skin gelatin, and successively incubated with primary and secondary antibodies in 1× PBS/0.05% Tween supplemented with 0.2% fish skin gelatin and 0.1% sodium azide for 1 h at room temperature, and washed after each incubation 3 times in 1× PBS/0.05% Tween (5 min each). Cells were then counterstained with DAPI. Primary antibodies were as follows: rabbit anti-LINE-1 ORF1 (1:1000; Abcam, ab216324) and rabbit anti-Cas9 (1:100; Recombinant Antibodies Platform at Institut Curie, A-P-R#56). Secondary antibodies were goat anti-rabbit coupled with Cy3 (1:200; Bethyl, A120-201C3) and goat anti-mouse coupled with DyLight 488 (1:200; Bethyl, A90-116D2).

Images were acquired using Zeiss LSM 710 microscope system (ZEISS Microscopy) equipped with a Plan-Apochromat DIC 63× NA 1.40 oil objective and ZEISS ZEN software. The images were processed in FIJI using a Z-projection (sum slices). For Cas9, the percentage of positive cells (with high or low signal) was manually counted in the population of cells treated with DOX. To quantify the L1-ORF1 signal, identical intensity threshold values were set in all images of each clone or condition, and the mean grey value was measured in 50–90 cells per clone/condition. The comparison of different conditions was based on the average of the mean grey values and their standard deviation. Statistical significance was assessed using a two-tailed unpaired *t* test.

### RNA FISH

ES cells were grown on coverslips for 48 h in the presence or absence of DOX (1 μg/ml). Cells were rinsed in 1× PBS, fixed for 10 min in 3.7% paraformaldehyde, rinsed in 1× PBS, and permeabilised for 5 min in 1× PBS/0.5% Triton on ice, in the presence of 1% VRC (Vanadyl Ribonucleoside Complex; NEB, S1402S). Coverslips were then rinsed three times in 70% EtOH and stored at −20 °C. RNA FISH was performed essentially as previously described (Chaumeil et al, 2008). Briefly, coverslips stored in 70% EtOH, were dehydrated in 80%, 95%, and 100% EtOH (5 min each) and then allowed to air-dry. For L1 RNA detection, a full-length L1MdTf element (L1spa) cloned into pBluescript (TNC7; Naas et al, 1998) was used. Probes were labelled by nick translation (Vysis) with Spectrum GreendUTP following the manufacturer’s instructions and precipitated in the presence of salmon sperm. Labelled probes were denatured for 5 min at 74 °C. Cells were then directly hybridised with probes overnight at 37 °C. After hybridization, coverslips were washed twice in 50% formamide/2× SSC and twice in 2× SSC at 42 °C. Cells were counterstained with DAPI.

Images were acquired using Zeiss LSM 710 microscope system (ZEISS Microscopy) equipped with a Plan-Apochromat DIC 63× NA 1.40 oil objective and ZEISS ZEN software. Images were processed in FIJI using a Z-projection (sum slices). Identical intensity threshold values were set in all images of each experiment and the mean grey value of TNC7 was measured in 80–200 cells per condition. The comparison of different conditions was based on the average of the mean grey values and their standard deviation. Statistical significance was assessed using a two-tailed unpaired *t* test.

### Proliferation and cell cycle assays

For proliferation assay, 5 × 10^5^ cells were plated at day 0 in a six-well plate in the presence or absence of DOX (1 μg/ml). Cells were trypsinized and counted every day for the next 4 days, replating 5 × 10^5^ cells each time into fresh culture. Trypan-Blue stained cells were also counted every day in this assay to quantify dead cells. Statistical significance was assessed using two-tailed paired *t* tests. For colony formation assay, 1000 cells were plated and left to form colonies over a period of 6 days in 12-well plates. Colonies were rinsed twice with 1× PBS, stained with 2% methylene blue (in 70% EtOH) and then rinsed in 70% EtOH to remove excess staining. For cell cycle analysis, ES cells were harvested from six-well plates following 48 h with or without DOX-treatment (1 μg/ml), washed with 1× PBS and stored at −20 °C in 70% EtOH. Prior to analysis, cells were treated with RNAse A (25 μg/ml in 1× PBS) for 20 min at 37 °C, then incubated with Propidium iodide (10 μg/ml in 1× PBS) for 20 min at 4 °C and analysed by flow cytometry on a BD Accuri C6 Plus instrument (BD Biosciences).

### In silico prediction of LKF and sgRNAs target sites

Sites for the LKF repressor were searched in the mm10 mouse genome using bowtie (Langmead et al, 2009) (version 1.2.3) with options: -f -v 0 -y -a. To identify sgRNAs predicted sites, alignments to the sgTf-mono2 and sgTf-mono3 sgRNAs sequences without considering the PAM sequence were first identified using RIsearch2 (Alkan et al, 2017) (version 2.1) with the following options: -s 1:20 -m 2:0 -e 10000 -l 0 --noGUseed -p3. RIsearch2 results were used as input for CRISPRoff (Alkan et al, 2018) (version 1.1.2) to detect sites with an NGG PAM sequence with options: --evaluate_all --no_azimuth. LKF and sgRNAs predicted sites were assigned to RepeatMasker annotations using bedtools intersect (Quinlan and Hall, 2010) (version 2.29.0) with options: -wao -f 1. RepeatMasker Viz. 2021 track data from UCSC were used to annotate 61 LKF repressor and 133 sgRNAs predicted target sites, given that they lacked associated elements in the RepeatMasker annotations (indicated by asterisks in Dataset EV1). To quantify the number of predicted target sites overlapping individual repeats, bedtools intersect was used with the -c option.

### ChIP

ES cells grown in 10 cm petri dishes in the presence or absence of DOX (1 μg/ml) for 48 h were harvested using trypsin, washed in 1× PBS and fixed with 1% (v/v) formaldehyde for 10 min at room temperature. Fixation was quenched with 0.125 M glycine for 5 min. Cells were then pelleted, washed three times with cold PBS, aliquoted (~10 × 10^6^ cells per Eppendorf tube per IP) and frozen in dry ice. Pellets were lysed in 350 µL buffer LB3 (10 mM Tris-HCl/pH 8.0, 100 mM NaCl, 1 mM EDTA, 0.5 mM EGTA, 0.1% Na-Deoxycholate, 0.5% N-lauroylsarcosine, 1× protease inhibitors) for 10 min at 4 °C on a rotating wheel. Samples were sonicated using a Bioruptor (Diagenode) for 30 min at maximum output (3 cycles of 10 min: 30 s on/off, with 5 min on ice between cycles) to reach a fragmentation between 200 and 500 bp. Lysates were then supplemented with 100 μL of buffer LB3, 50 μL of 10% Triton X-100 and centrifuged at full speed for 5 min at 4 °C; the supernatant (sonicated chromatin) was collected, and 10% was saved as input. In parallel, 50 μL Dynabeads Protein G (Invitrogen) per IP were washed and resuspended in 400 μL PBS with 0.5% BSA, then blocked for 1 h at room temperature with antibodies for IP (Cas9, Active Motif AB_2793760: 4 µg/IP; Normal Mouse IgG, Merck 12-371: 4 µg/IP). Antibody-conjugated beads were resuspended in 50 μL of buffer LB3 and incubated with chromatin overnight at 4 °C on a rotating wheel. Beads were washed subsequently twice with TF-WBI (20 mM Tris-HCl/pH 7.4, 150 mM NaCl, 0.1% SDS, 1% Triton X-100, 2 mM EDTA), twice with TF-WBIII (250 mM LiCl, 1% Triton X-100, 0.7% Na-DOC, 10 mM Tris-HCl, 1 mM EDTA), and twice with TET (0.2% Tween-20, 10 mM Tris-HCl/pH 8.0, 1 mM EDTA). Chromatin was eluted from beads with 70 μL elution buffer (0.5% SDS, 300 mM NaCl, 5 mM EDTA, 10 mM Tris-HCl/pH 8.0) containing 4 μL proteinase K (10 mg/ml) for 1 h at 55 °C, followed by overnight incubation at 65 °C to reverse crosslinking. A second elution was performed with 30 μL of the same buffer for 1 min, and both eluates were pooled. DNA was subsequently purified using Qiagen MinElute columns according to the manufacturer’s instructions. qPCR was used to confirm successful immunoprecipitation using primers for known targets and negative loci (Dataset EV11).

### ChIP-seq and data processing

For sequencing, DNA fragments were repaired, A-tailed, ligated with Illumina adapter, and sequenced (paired-end, 2 × 150 bp) on a NovaSeq X plus by Novogene Co., with 90–100 million paired reads per IP sample and 40 million paired reads per input sample on average. Libraries were processed using trim_galore (https://github.com/FelixKrueger/TrimGalore) (version 0.6.10) to remove sequencing adapters, low-quality base pairs and too short reads with the following parameters: --stringency 1 --length 105 --quality 30. Filtered reads were mapped to the mm10 genome using bowtie2 ((Langmead and Salzberg, 2012); https://github.com/BenLangmead/bowtie2) (version 2.5.4) with the following parameters: --very-sensitive --end-to-end -X 400 --no-mixed --no-discordant. As the default reporting parameters were not modified, Bowtie2 searched for multiple valid alignments and reported the best one, with equally good alignments resolved pseudo-randomly. Therefore, reads originating from young L1 elements may be assigned to one of several equivalent genomic locations. After mapping, mitochondrial reads were filtered out using samtools view (Li et al, 2009) (version 1.20) and the grep command (grep -v chrM), and duplicates were removed using Picard tools (MarkDuplicates and REMOVE_DUPLICATES) (“Picard Toolkit.” 2019. Broad Institute, GitHub Repository. https://broadinstitute.github.io/picard/) (version 3.3.0). To obtain the set of uniquely mapped reads, BAM files were further filtered using the following criteria: minimum MAPQ of 1 and absence of the “XS:i” flag. Finally, BAM files of samples sequenced in multiple runs were merged. Raw (FASTQ files) and processed data were deposited in GEO under accession number GSE212329.

### ChIP-seq peak calling

Peak calling was performed with MACS ((Zhang et al, 2008); https://github.com/macs3-project/MACS) (version 2.2.9.1) on filtered IP reads from both uniquely mapped and multimapping read sets, using the corresponding input sample as control. The following options were used: -f BAMPE --keep-dup all --call-summits. Replicates were treated as pooled samples for peak calling using uniquely mapped reads. Narrow peaks were retained after excluding those overlapping the ENCODE mm10 blacklist. Peaks with a signal value < 18 for the uniquely mapped reads and <27 for the multimapping reads were removed. For the analysis of the VPR-D4 ( + DOX) condition including multimapping reads, one replicate contained comparatively fewer peaks above this threshold; therefore, peaks shared with the other replicate that passed the filtering criteria were also retained. Bedtools (closest and intersect functions; version 2.31.1) was used to annotate peaks with BED files containing L1 annotations from Repeat Masker, and to compare peaks with in silico predicted targets.

### RNA-seq

All five selected ESC clones were sequenced in biological duplicates. For each sample, total RNA (800 ng) was used for library preparation using the Illumina TruSeq stranded total RNA Library Prep kit following the manufacturer’s protocols. Sequencing was performed in 100 bp paired-end reads using a Novaseq 6000 instrument, with 90–100 million paired reads per sample on average. Raw (FASTQ files) and processed data were deposited in GEO under accession number GSE212329.

### DNA-seq

Genomic DNA was extracted from PGK12.1 mESCs using the DNeasy blood and tissue kit (Qiagen), following the manufacturer’s instructions. The DNA-seq library for whole genome sequencing was prepared with 1 µg of genomic DNA, using the Illumina TruSeq DNA Library Prep kit, following the manufacturer’s instructions. Sequencing was performed in 100 bp paired-end reads using an Illumina HiSeq 2500 instrument. Raw sequencing data are available through the NIH BioProject accession number PRJNA875055.

### SNP calling

Read pairs from the DNA-seq library were trimmed using trim_galore (version 0.6.3) and mapping to the mm10 genome was performed using BWA mem (Li and Durbin, 2009) (version 0.7.17-r1188) with parameters: -M -k 22. Alignments were saved in BAM format and filtered using samtools view (version 1.9) with parameters: -q 20 -f 2. In addition, alternative hits and mitochondrial reads were removed as follows: grep -v -e ‘XA:Z:’ -e ‘SA:Z:’ -e ‘chrM’. Filtered BAM files were sorted with samtools sort and duplicates removed with Picard MarkDuplicates (version 2.20.8). To identify 129/PGK SNPs, bcftools (Li, 2011) (version 1.9) and freebayes (Garrison and Marth, 2012) (version 1.3.2) were implemented. For bcftools, the BAM file and the mm10 genome were indexed with samtools index and samtools faidx, respectively. Likelihoods were computed and variants called by first running bcftools mpileup with option -Ou, and then bcftools call with options: -mv -Ob. Variants were filtered based on quality using bcftools view with the following parameters: -i ‘%QUAL > = 19’ -Ob. Variant calling with freebayes was performed with default parameters and the results were also filtered by quality with vcffilter (Garrison et al, 2022) (version 1.0.0) with option -f -f “QUAL > 19”. Variants obtained with bcftools and freebayes were filtered to only keep heterozygous SNPs using bcftools view and the following parameters: -v snps -g het -M2. Heterozygous SNPs from both programs were merged using bcftools merge and converted to BED format, to generate a list of coordinates with all possible heterozygous sites (Dataset EV12), which was used to mask the reference genome for read mapping and minimize mapping biases. In addition, the common set of SNPs from both programs was identified using bcftools isec with option -n = 2. These SNPs were filtered by quality using the values computed by freebayes as follows: vcffilter -f “QUAL > 20 & DP > 10 & SAF > 0 & SAR > 0 & RPR > 1 & RPL > 1”. Public 129 SNP reference annotations were downloaded from the Wellcome Sanger Institute website (129P2_OlaHsd.mgp.v5.snps.dbSNP142.vcf.gz), to define which of the identified alleles corresponded to the 129 genotype. Only those variant positions in common with the published reference were used to assign allele-specific reads. Finally, common SNPs overlapping low complexity regions (defined by RepeatMasker annotations) were filtered out using bedtools intersect with option -v. Thus, a total of 1,019,011 common heterozygous SNPs were identified for 129/PGK (Dataset EV13).

### RNA-seq data processing

Libraries were processed to remove sequencing adapters from read pairs using trim_galore. Then, to remove potential rRNA contamination, reads were mapped to a reference of rDNA annotations using STAR (Dobin et al, 2013) (version 2.7.2b) with the following parameters: --alignIntronMax 500000 --alignMatesGapMax 500000 --alignEndsType EndToEnd --winAnchorMultimapNmax 2000 --outFilterMultimapNmax 10000 --outFilterMismatchNmax 999 --outFilterMismatchNoverLmax 0.2 --seedMultimapNmax 20000 --outSAMtype None --outReadsUnmapped Fastx. Filtered reads were mapped to the mm10 genome using an N-masked reference that was generated based on the merged SNP annotations of the PGK12.1 cell line (Dataset EV12) using bedtools maskfasta (version 2.29.2). To quantify gene and single repeat element expression, read pairs were mapped to the N-masked genome using STAR to report uniquely mapped reads: --alignIntronMax 500000 --alignMatesGapMax 500000 --alignEndsType EndToEnd --outFilterMultimapNmax 1 --outFilterMismatchNmax 999 --outFilterMismatchNoverLmax 0.03 --outSAMattributes NH HI NM MD --outSAMmultNmax 1 --outSAMtype BAM Unsorted. On the other hand, to quantify repeat expression by family/subfamily, multimapped read pairs were saved by randomly assigning them to one of the possible hits using the following parameters: --alignIntronMax 500000 --alignMatesGapMax 500000 --alignEndsType EndToEnd --winAnchorMultimapNmax 2000 --outFilterMultimapNmax 10000 --outFilterMismatchNmax 999 --outFilterMismatchNoverLmax 0.03 --seedMultimapNmax 20000 --outSAMattributes NH HI NM MD --outSAMmultNmax 1 --outSAMtype BAM Unsorted. BAM files with mapped reads were filtered to remove singletons with samtools view and the following options: -b -f 0x2. In addition, mitochondrial reads were filtered out using samtools view and the grep command (grep -v chrM). To assign read pairs to specific alleles, filtered BAM files and the 129/PGK SNP annotations (Dataset EV13) were used as input for SNPsplit (Krueger and Andrews, 2016) (version 0.3.4) with the following options: --paired --no_sort. SNPsplit generated one BAM file for each allele (129 and PGK) and one for unassigned reads. These 3 BAM files were merged using samtools merge to generate a BAM file with total signal. BAM files were sorted by coordinate using picard SortSam. Total and allele-specific quantifications of gene and repeat expression were performed using featureCounts (Liao et al, 2014) (version 2.0.1) with ENSEMBL gene annotations (Cunningham et al, 2022) (v98) and RepeatMasker annotations from the UCSC genome browser (Nassar et al, 2023) (discarding simple, low complexity and exonic repeats), respectively, and with the following parameters: (a) for genes, -C -p -s 2 -t exon -g gene_id; (b) for single repeats, -C -p -s 2; (c) for repeat families/subfamilies, -C -p -s 2 -M. Repeat coordinates were specified as repeat IDs in the SAF file used by featureCounts. Family/subfamily quantification was obtained by summing counts of all repeats according to their classification. Read counts of single repeats were used to compute Transcripts Per Million (TPM) values and obtain their expression fold change upon DOX treatment. Signal profiles were generated for each library and by strand using bamCoverage (Ramirez et al, 2016) (version 3.1.3), a scaling factor (100000000/library size) and the following parameters: --binSize 1 --normalizeUsing CPM --filterRNAstrand [forward or reverse] --effectiveGenomeSize 2467481108 --outFileFormat bigwig --scaleFactor [computed for each library].

### Normalization by repeat family and subfamily

Read count tables of repeat families and subfamilies were processed to add up reads of repeat elements grouped by family or subfamily and obtain their total quantification. The two tables generated were independently joined with the read count table of genes to perform normalization. The R package DESeq2 (Love et al, 2014) (version 1.26.0) was used to calculate normalized counts for all libraries by clone. Normalized counts were extracted from the DESeqDataSet using the function counts with the option normalized=TRUE, and only the normalized counts of repeat families and subfamilies were used for further analyses.

### Differential gene and repeat expression

Contrast analyses of libraries with and without DOX treatment by clone were performed using DESeq2 (Love et al, 2014). To generate one input table for DESeq2, read count tables of genes and single repeats were joined. Genes and repeats with less than 10 total counts across libraries were discarded for comparison. DE analysis was performed using the DESeq function and computed values extracted using the results function. To calculate corrected *P* values, genes and repeats discarded by independent filtering or considered as outliers (NA values in *P* value and adjusted *P* value columns) were removed from the results table. Then, the full column of adjusted *P* values was deleted and new *P* values were estimated using the Z-scores with the function fdrtool setting statistic = “normal”. Adjusted *P* values were then computed using the Benjamini–Hochberg method with the function *P*adjust. Results of genes were extracted and visualized using volcano plots generated with the function EnhancedVolcano (https://github.com/kevinblighe/EnhancedVolcano) and setting the option pCutoff = 0.01, to add a line at the adjusted *P* value threshold used to consider genes as differentially expressed.

### Metagene plot signal

To generate repeat expression profiles, coordinates of differentially expressed L1MdTfs by strand and clone were converted to BED format. In the case of the repressor clone, only the downregulated repeats were considered, whereas for the activator clones, only the upregulated repeats were considered. deepTools (version 3.4.3) (Ramirez et al, 2016) was used to compute average values and generate two signal plots by clone, one in sense and one in antisense orientation. First, average values by strand were calculated using computeMatrix scale-regions with options: -m 6000 -b 2000 -a 2000 --missingDataAsZero. For sense transcription, signal profiles (bigWig files) corresponding to the strand of the annotated L1MdTfs were used as computeMatrix input, while signal profiles of the opposite strand of the annotated L1MdTfs were used to calculate the antisense signal. Then, values obtained for each of the BED files containing DE L1MdTfs in the forward and reverse strand were joined using computeMatrixOperations rbind. Finally, plots were generated using plotProfile with options: --averageType mean --perGroup.

### Clustering of genes and repeats

Fold changes of DE genes and L1MdTf repeats were used to compute Z-scores, to perform K-means clustering using the kmeans function in R (R Core Team, 2023) (version 3.6.1). The number of clusters was selected based on visual assessment (heat maps) of the generated clusters. The joint table of raw read counts of genes and single repeats of all clones was filtered to remove features with less than ten total reads across libraries. A regularized log transformation was applied to this filtered table using the DESeq2 rlog function with option: blind=FALSE. Normalized read counts of DE genes and L1MdTf repeats were extracted and used to generate gene expression heat maps based on the order obtained by clustering their corresponding Z-scores.

### Distance analyses

Genomic distances between L1MdTf repeats and all genes and non-L1 repeats, which were included in the DE analyses, were calculated using bedtools (version 2.29.0) function closest with the following parameters: -d -t “first”. BED files used for these analyses contained the coordinates of full gene/repeat annotations. Genes and non-L1 repeats were classified according to the DE results, significance of the observed differences in the distance distribution of classes was assessed using Wilcoxon-test (wilcox.test function in R; null hypothesis: the compared distributions differ by a location shift of zero). To test the dependence between distance and gene/repeat classification, Chi-squared tests were performed using the chisq.test function in R with option: simulate.*p*.value = TRUE; null hypothesis: no association between distance and classification. Residuals from this test were plotted using the corrplot package (https://github.com/taiyun/corrplot) (version 0.88). Associations between transcriptional status and proximity to predicted targeted or non-targeted L1MdTfs on Fig. EV3A were assessed using Fisher’s exact test, comparing each category to the “No change” reference group. *P* values were then corrected for multiple comparisons using the Bonferroni method.

### TAD and chromatin loop annotations

Publicly available TAD annotations (*n* = 2712) from Hi-C data of mESCs (Bonev et al, 2017) were classified by the presence/absence of DEGs (up- or downregulated independently) and L1MdTfs (targeted according to in silico predictions). For each group of TADs with or without DEGs, the percentages harboring predicted targeted L1MdTfs were calculated for visualization. Chi-squared tests of independence were performed to test for associations between these two features in the activation and repression systems (null hypothesis: no association between TAD classifications based on DEGs and L1MdTfs). Processed Hi-C data from Bonev et al, generated by Juicer (Durand et al, 2016) was downloaded from the Aiden Lab repository (http://hicfiles.s3.wasabisys.com/external/bonev/ES_mapq30.hic), and analyzed with Mustache (Roayaei Ardakany et al, 2020) using the default KR normalization (-r 5 kb -pt 0.01) to call loops. Overlap between loop anchors, DEGs and predicted targeted L1MdTf elements was assessed using bedtools intersect.

### Chromatin state analysis

The ChromHMM model of ESCs used for analyses was downloaded from: https://github.com/guifengwei/ChromHMM_mESC_mm10. Fold enrichment of DE genes and repeats in the 12 annotated states was tested using ChromHMM OverlapEnrichment (Ernst and Kellis, 2017) (version 1.23).

### Gene ontology enrichment analysis

Enrichment analyses of GO terms, KEGG pathways and Uniprot tissue expression were performed with the Database for Annotation, Visualization and Integrated Discovery – DAVID (Sherman et al, 2022) (version 6.8) by providing the ENSEMBL IDs of DE genes in the activator system and using the whole mouse genome as background. Results tables were downloaded from the online tool and adjusted *P* values, fold enrichment values and number of genes by term were used for visualization. To correct for gene length bias in GO enrichment analysis, GOseq (run in Galaxy; (Young et al, 2010)) and GOglm (https://github.com/gu-mi/GOglm/tree/master; (Mi et al, 2012)) programs were used with default settings.

### Allelic expression analyses

Allele-specific repeat and gene read counts of 129 and PGK obtained with featureCounts were used to calculate allelic ratios and test for differences from the expected biallelic expression with DESeq2. For each clone, a DDS object was generated using its read count table and a design matrix specifying the DOX treatment, replicate information and the allele. The design formula for contrasts was set as follows: ~treatment + treatment:replicate + treatment:allele. Genes and repeats with less than 10 reads across samples were removed from the DDS object. Then, size factors were set to 1 and significance testing performed with the DESeq function. Results tables were extracted with the results function and the following options: alpha = 0.01, filterFun = ihw (Ignatiadis et al, 2016). The obtained adjusted *P* values represent genes or repeats with significant bias from biallelic expression (allelic score = 0.5). PGK/129 ratios were calculated from the log2 fold changes in the results table and allelic scores were then calculated as: (PGK/129)/(1+PGK/129). Genes and repeats with adjusted *P* values lower than 0.01 were considered as features with skewed allelic expression.

### Data visualization

Results from bioinformatics analyses were plotted using R (version 3.6.1) and the ggplot2 package (https://ggplot2.tidyverse.org/) (version 3.1.1).

## Supplementary information

Appendix

Peer Review File

Dataset EV1

Dataset EV2

Dataset EV3

Dataset EV4

Dataset EV5

Dataset EV6

Dataset EV7

Dataset EV8

Dataset EV9

Dataset EV10

Dataset EV11

Dataset EV12

Dataset EV13

Source data Fig. 1

Source data Fig. 2

Source data Fig. 3

Source data Fig. 4

Source data Fig. 5

Source data Fig. 6

Expanded View Figures

## Data Availability

Raw RNA-seq and ChIP-seq data generated in this study have been deposited in NCBI’s Gene Expression Omnibus (Edgar et al, 2002) and are accessible through GEO Series accession number GSE212329. DNA-seq data have been deposited in NCBI’s Sequence Read Archive (Katz et al, 2022) under study accession SRX17869356. Both datasets have links to BioProject accession number PRJNA875055 in the NCBI BioProject database. The source data of this paper are collected in the following database record: biostudies:S-SCDT-10_1038-S44319-026-00854-w.
