# Supplementary material for: Transcriptional perturbation of LINE-1 elements reveals their cis-regulatory potential
Source: EMBO Rep. 2026 Jul 8;27(16):4824–54. doi: 10.1038/s44319-026-00854-w (PMC13503937; doi:10.1038/s44319-026-00854-w)
Supplement: Supplementary file 1 [file 44319_2026_854_MOESM1_ESM.pdf]

Appendix for:

Transcriptional perturbation of LINE-1 elements reveals their *cis*-regulatory potential

Yuvia A Pérez-Rico, Anna Le Breton, Aurélie Bousard, Lenka Henao Misikova, Eskeatnaf Mulugeta, Sérgio F de Almeida, Alysson R Muotri, Edith Heard and Anne-Valerie Gendrel*

* Correspondence: anne-valerie.gendrel@gimm.pt

Table of contents:

Appendix Figure S1……………………………………………………………………..**Page 2**

Appendix Figure S2……………………………………………………………………..**Page 4**

Appendix Figure S3……………………………………………………………………..**Page 6**

Appendix Figure S4……………………………………………………………………..**Page 8**

Appendix Figure S5…………………………………………………………………….**Page 10**

Appendix Figure S6…………………………………………………………………….**Page 12**

Appendix Figure S7…………………………………………………………………….**Page 14**

Appendix Figure S1

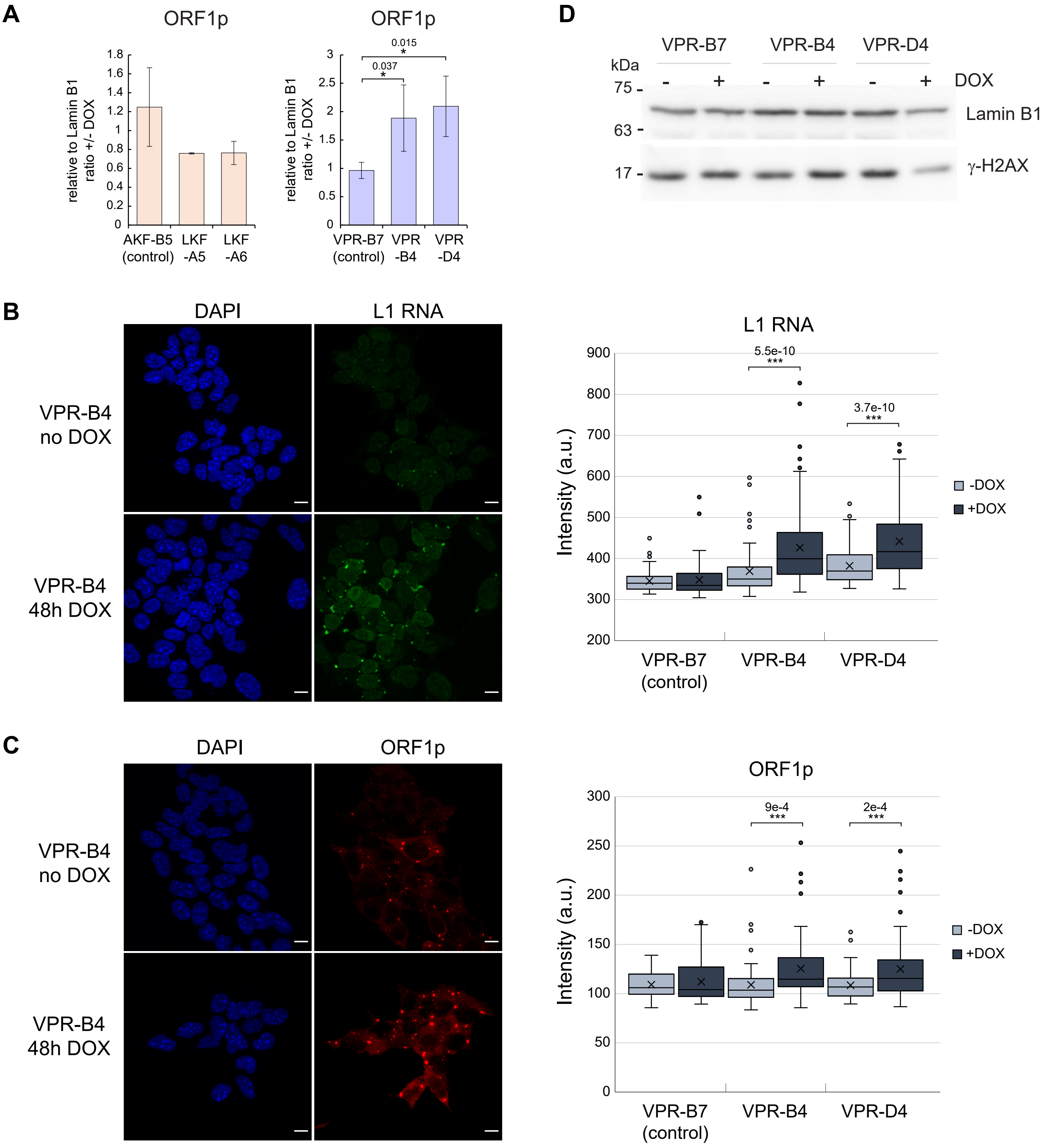

Appendix Figure S1- Visualization of L1 RNA and ORF1p expression in control and activator clones. (A) Quantification of Western blots shown in Fig.2D (left panel) and Fig.2F (right panel). The signals from the two ORF1p bands were combined for analysis. For each clone, the DOX to non-DOX signal ratio was calculated and normalized to the corresponding Lamin B1 signal (n=3 biological replicates for repressor clones & VPR-B7; n=4 for VPR-B4 & VPR-D4). **(B)** Representative images of L1 RNA FISH in activator clone VPR-B4 in the presence or absence of DOX for 48 h (left) (scale bar, 10 µm). Quantification of L1 RNA signal, detected with full-length L1 probe (TNC7), in control and activator clones based on the average of the mean grey values per cell and their standard deviation for each clone/condition (n=80-200 cells) (right). **(C)** Representative images of ORF1 immunofluorescence in activator

clone VPR-B4 in the presence or absence of DOX for 48 h (left) (scale bar, 10 µm). Quantification of ORF1 signal in control and activator clones based on the average of the mean grey values per cell and their standard deviation for each clone/condition (n=50-90 cells) (right). **(D)** Western blot analysis showing no change in gH2AX levels in activator clones VPR-B4 and VPR-D4, after DOX treatment for 48 h. LaminB1 is used as loading control. This experiment was repeated twice with similar results. Statistics: two-tailed unpaired t-test (panel A), with Welch corrections (panels B, C): * $p<0.05$, ** $p<0.01$, *** $p<0.001$; null hypothesis: there is no difference in L1 RNA and ORF1p levels between −DOX and +DOX conditions.

Appendix Figure S2

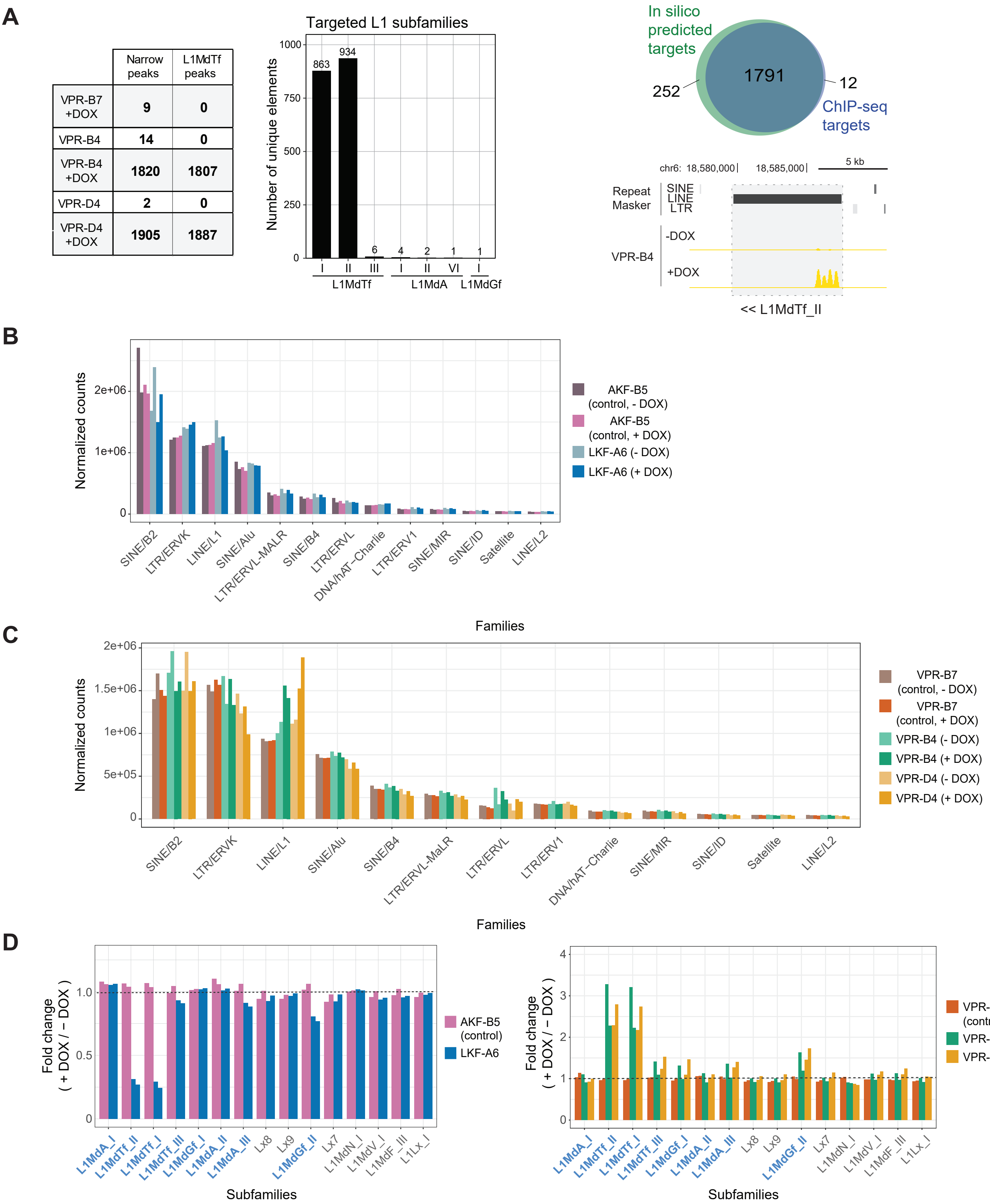

Appendix Figure S2- dCas9-VPR off-target assessment and analysis of repeat expression by family and subfamily. (A) Results of ChIP-seq analyses including multimapping reads. Left: number of total narrow peaks and those overlapping L1MdTfs by VPR clone and condition. Middle: estimation of the number of young L1Md unique elements bound by dCas9 in clones VPR-B4 and VPR-D4 upon DOX-induction. Left: Venn diagram showing near-pearfect overlap between in silico predicted targets and observed bound targets by

ChIP-seq, and an example of a peak within the 5'UTR of an L1MdTf located on chromosome 6. **(B)** Bar plot showing normalized read counts (y-axis) per repeat family in repressor (LKF-A6) and control (AKF-B5) clones with and without DOX treatment. **(C)** Bar plot showing normalized read counts (y-axis) per repeat family in activator (VPR-B4, VPR-D4) and control (VPR-B7) clones with and without DOX treatment. **(D)** Bar plots showing fold change of normalized read counts after DOX treatment (y-axis) per L1 subfamily in repressor (left) and activator (right) clones with their respective controls. Dash lines mark values equal to 1.

Appendix Figure S3

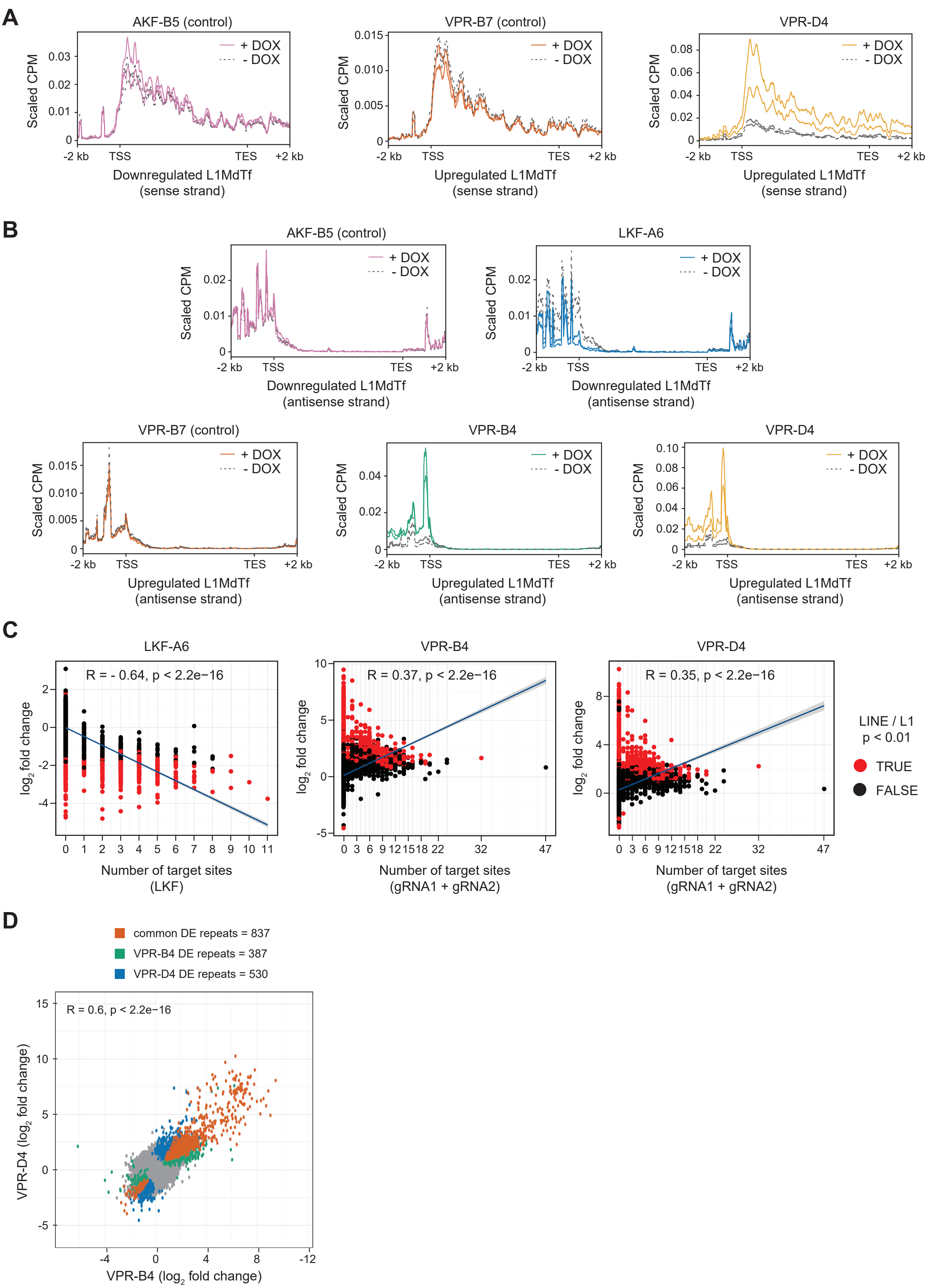

Appendix Figure S3- Analysis of L1MdTf repeat expression after DOX treatment. (A) Metagene plot of normalized RNA-seq signal (shown for AKF-B5, VPR-B7 and VPR-D4 clones, replicates A & B) on L1MdTf repeats showing downregulation in LKF-A6 (left) or

upregulation in the activator clones (middle, right) upon DOX treatment. The x-axis is scaled to represent full annotations and the 2 kb upstream and downstream regions. **(B)** Metagene plot of normalized RNA-seq signal (shown for all clones sequenced, replicates A & B) on the antisense strand of L1MdTf repeats showing downregulation in the LKF-A6 clone (top) or upregulation in the activator clones (bottom) upon DOX treatment. The x-axis is scaled to represent full annotations and the 2 kb upstream and downstream regions. **(C)** Correlation between expression fold change and number of LKF (left) or gRNA (middle, right) predicted target sites for L1 repeats with (red) and without (black) significant changes in expression in repressor (left) and activator (middle, right) clones upon DOX treatment. Pearson correlation values and their significance from t-tests are displayed; null hypothesis: the correlation is due to chance. **(D)** Comparison of expression fold changes of repeats in the VPR-B4 and VPR-D4 clones after DOX treatment. Grey dots represent repeats with no significant changes in expression and colored dots represent repeats identified as DERs in both clones or in one of the clones only. Pearson correlation coefficient and significance from t-test are shown; null hypothesis: the correlation is due to chance.

Appendix Figure S4

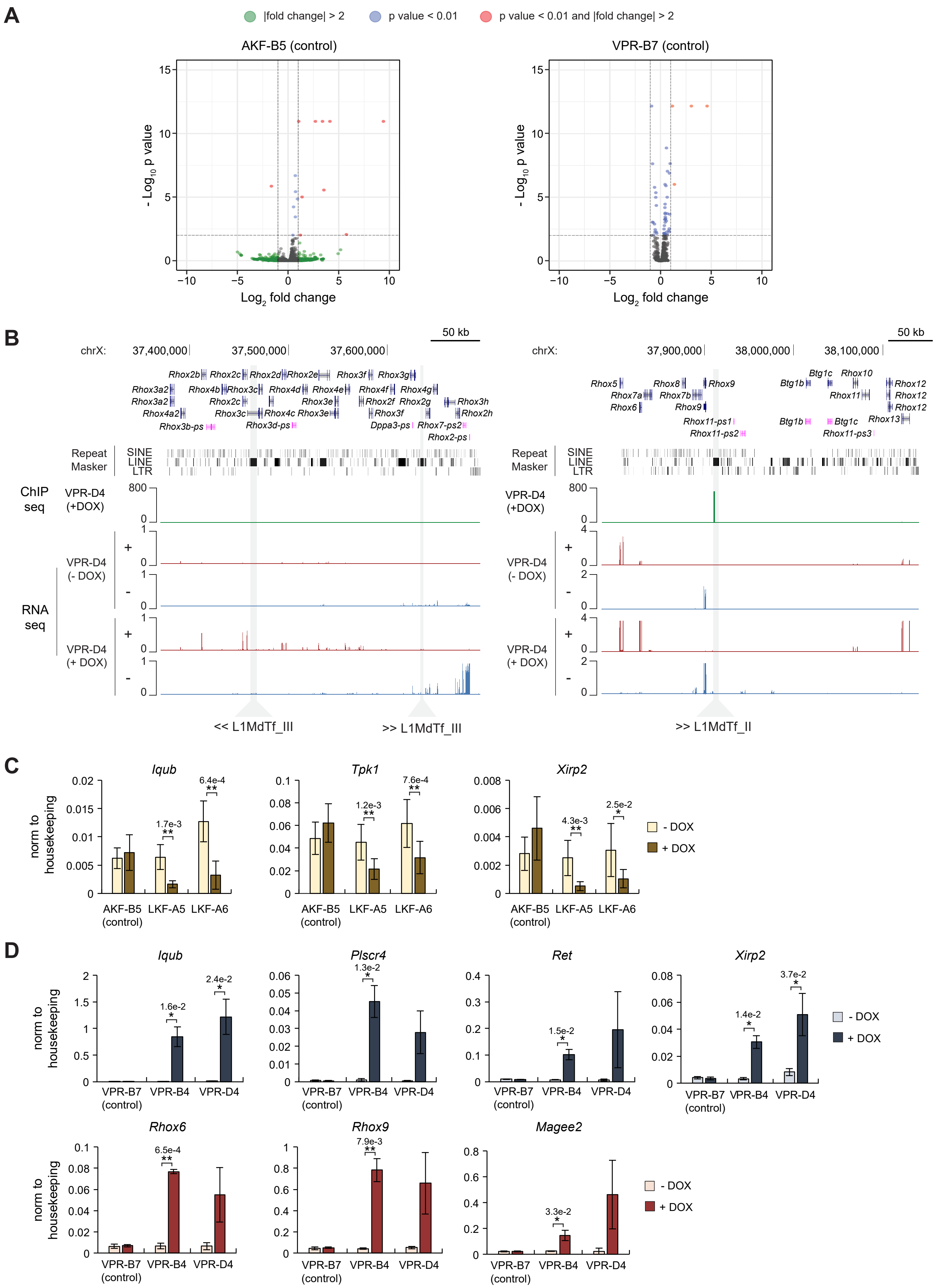

Appendix Figure S4- DE analysis of genes in control clones and examples of selected and validated DEGs. (A) Volcano plots showing gene expression fold changes (x-axis) and their significance (y-axis) in the repressor AKF-B5 (left) and activator VPR-B7 (right) control clones. **(B)** Examples of upregulated genes in the *Rhox* cluster on the X chromosome following activation of L1MdTf repeats. ChIP-seq for dCas9 (VPR-D4 clone, +DOX) profile is shown in green (uniquely mapped reads). RNA-seq profiles by strand are shown in red (+ strand) and blue (- strand) for the DOX-treated and untreated samples. L1MdTf repeats located in the cluster and their orientation are highlighted. **(C)** qPCR results for selected genes showing specific downregulation in repressor clones, following DOX treatment for 48 h. Levels are normalized relative to housekeeping gene expression. Error bars represent the calculated standard deviation considering 6 biological replicates. **(D)** qPCR results for selected genes (top, autosomal genes; bottom, X-linked genes) showing specific upregulation in the activator clones, following DOX treatment for 48 h. Levels are normalized relative to housekeeping gene expression and error bars represent the calculated standard deviation considering 3 biological replicates. Statistics: two-tailed paired t-test (panel D): * $p<0.05$, ** $p<0.01$; null hypothesis: there is no difference in expression levels between −DOX and +DOX conditions.

Appendix Figure S5

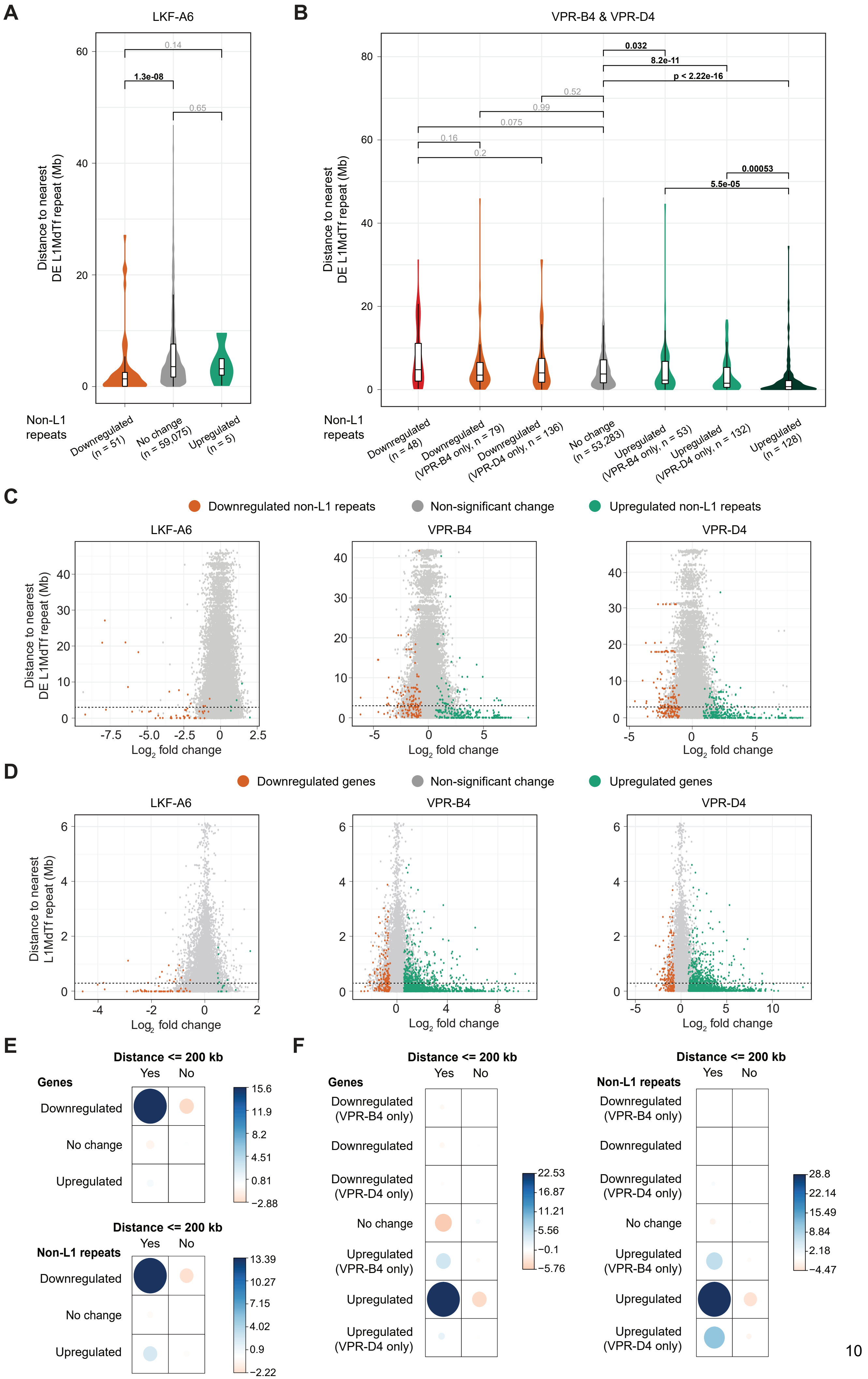

Appendix Figure S5- Distance analyses between non-L1 DERs and DE L1MdTf repeats. **(A)** Distribution of distances between DE L1MdTf repeats and non-L1 repeats classified according with DE analysis results in the LKF-A6 clone. Significance values of the differences between distributions are displayed above violin plots and were calculated using two-sided Wilcoxon rank-sum tests (statistically significant values are in bold); null hypothesis: the compared distributions differ by a location shift of zero. **(B)** Distribution of distances between DE L1MdTf repeats and non-L1 repeats classified according with DE analysis results in the activator clones. Significance values as described for panel A. **(C)** Comparison between expression fold change upon L1MdTf repression (LKF-A6) or activation (VPR-B4, VPR-D4) and distance to nearest DE L1MdTf repeat for all non-L1 repeats tested for DE. Dashed lines indicate a distance of 3 Mb. **(D)** Comparison between expression fold change upon L1MdTf repression or activation and distance to nearest annotated L1MdTf repeat for all genes tested for DE. Dashed lines indicate a distance of 260 kb. **(E)** Pearson residuals from Chi-squared tests (null hypothesis: no association between the features of interest) representing the relevance of attributes (i.e. classification based on expression and distance) to the total Chi-squared score for genes (top) and non-L1 repeats (bottom) in the repressor clone. Bigger circles depict higher contribution of table cells to the score. Colors indicate positive and negative association between rows and columns. **(F)** Pearson residuals from Chi-squared tests as described on panel E for genes and non-L1 repeats in the activator clones.

Appendix Figure S6

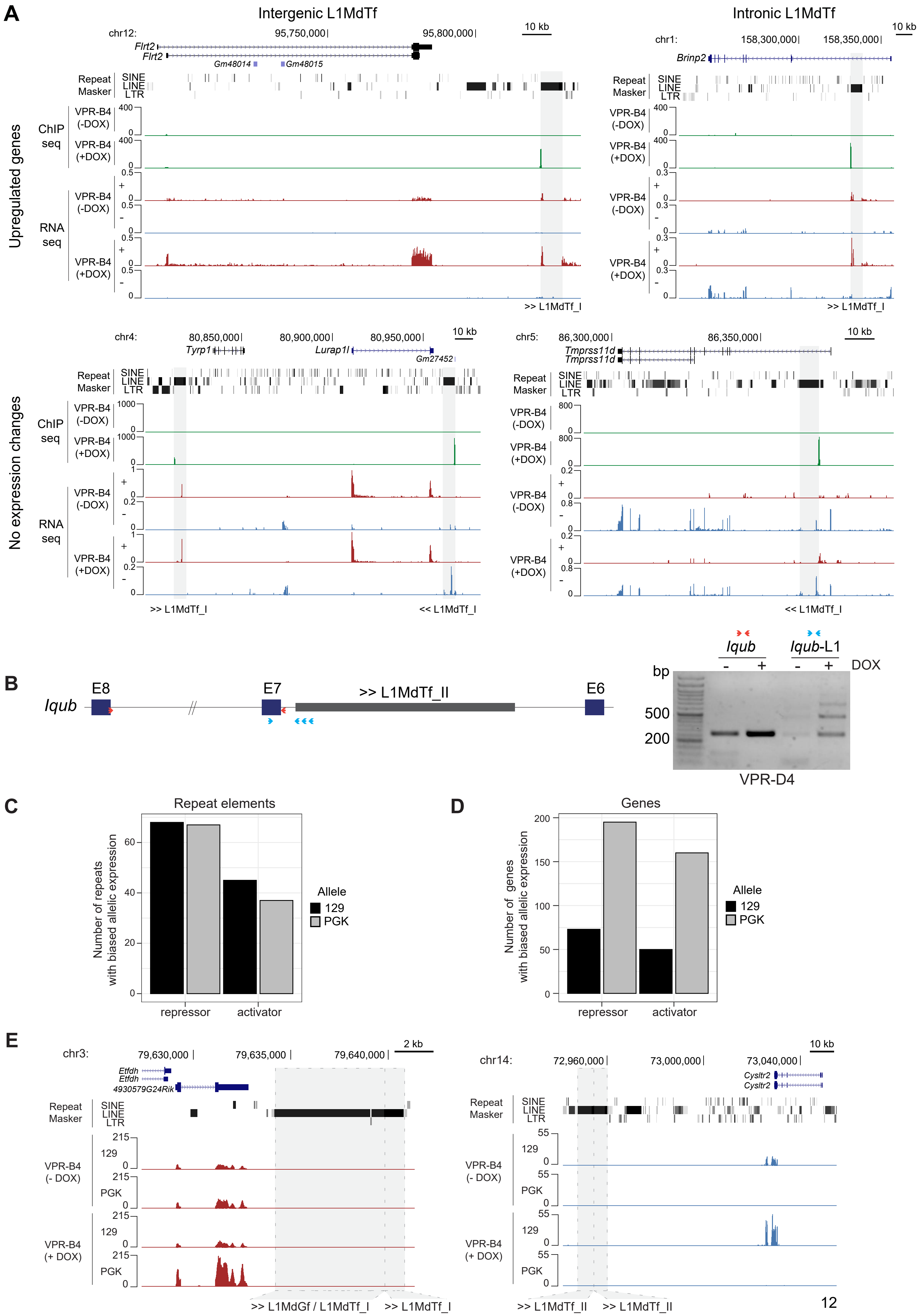

Appendix Figure S6- Impact of L1MdTf expression perturbation on neighboring gene expression and allele-specific transcription. (A) Genome browser ChIP-seq for dCas9 (in green, uniquely mapped reads) and RNA-seq (in red for + strand; blue for - strand) tracks showing examples where DOX-induced dCas9 binding at nearby intergenic or intronic L1MdTf elements is associated with either gene upregulation (*Flrt2* & *Brinp2*) or no significant change (*Tyrp1*, *Lurap1l* & *Tmprss11d*). **(B)** Semi-quantitative RT-PCR analysis of the *Iqub* locus in the VPR-D4 clone using two different sets of primers. The red primer pair overlaps *Iqub* exonic regions and yields a PCR amplicon of 263 bp, while the blue primer pair overlaps an *Iqub* intron and the repetitive monomers within the 5'UTR of the intronic L1 element, yielding products of 254, 466 and 678 bp. **(C)** Repeat elements with skewed allelic expression in L1MdTf repressing and activating conditions. Allele classification (129 or PGK) shows the allele from which elements are preferentially expressed. **(D)** Genes with skewed allelic expression as described in C. **(E)** Genome browser screenshots for the *4930579G24Rik* and *Cysltr2* genes, which show skewed expression following dCas9-VPR-mediated activation of L1MdTf repeats.

Appendix Figure S7

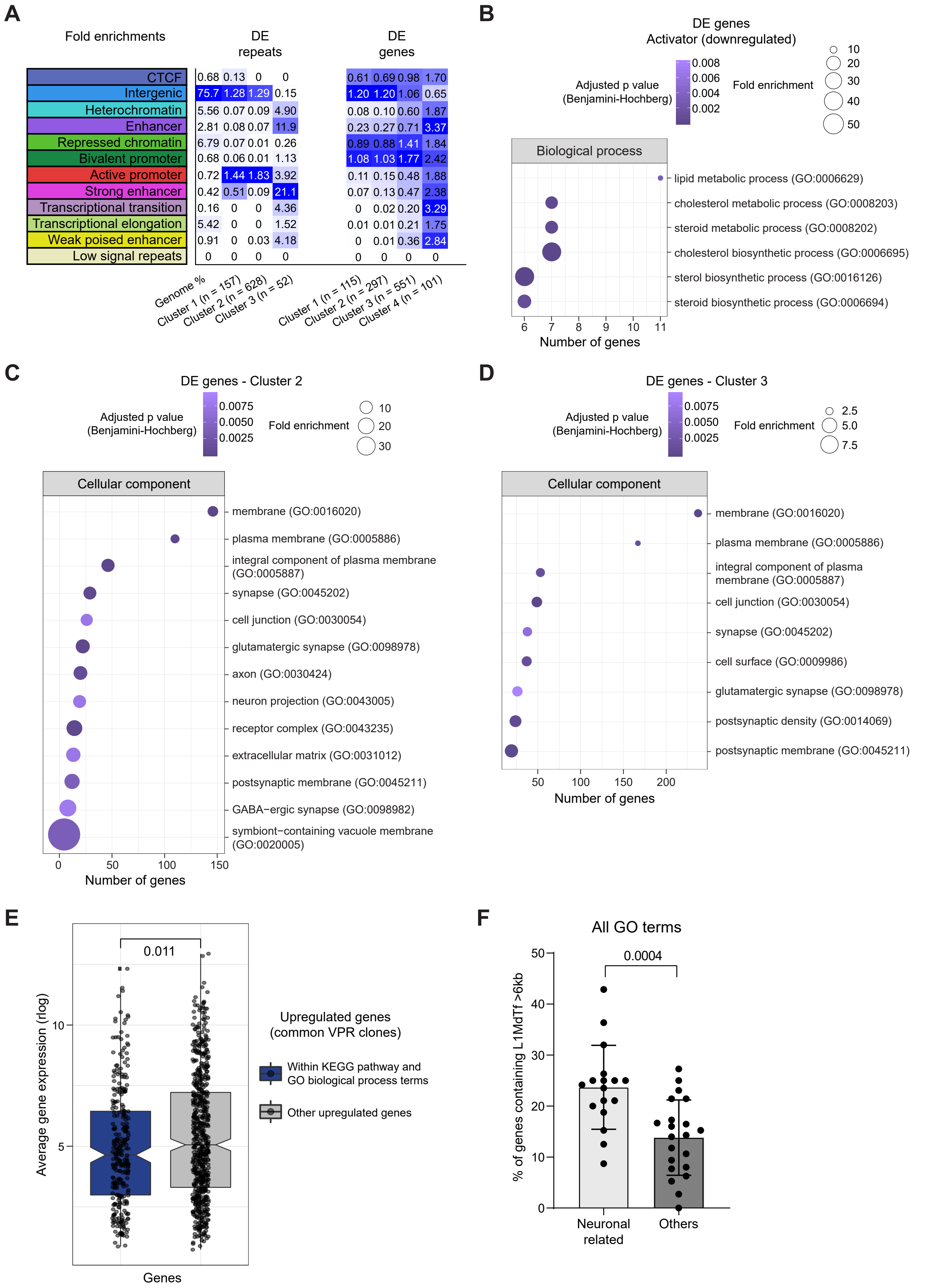

	Genome %	Cluster 1	Cluster 2	Cluster 3	Cluster 1	Cluster 2	Cluster 3	Cluster 4
CTCF	0.68	0.13	0	0	0.61	0.69	0.98	1.70
Intergenic	75.7	1.28	1.29	0.15	1.20	1.20	1.06	0.65
Heterochromatin	5.56	0.07	0.09	4.90	0.08	0.10	0.60	1.87
Enhancer	2.81	0.08	0.07	11.9	0.23	0.27	0.71	3.37
Repressed chromatin	6.79	0.07	0.01	0.26	0.89	0.88	1.41	1.84
Bivalent promoter	0.68	0.06	0.01	1.13	1.08	1.03	1.77	2.42
Active promoter	0.72	1.44	1.83	3.92	0.11	0.15	0.48	1.88
Strong enhancer	0.42	0.51	0.09	21.1	0.07	0.13	0.47	2.38
Transcriptional transition	0.16	0	0	4.36	0	0.02	0.20	3.29
Transcriptional elongation	5.42	0	0	1.52	0.01	0.01	0.21	1.75
Weak poised enhancer	0.91	0	0.03	4.18	0	0.01	0.36	2.84
Low signal repeats	0	0	0	0	0	0	0	0

Appendix Figure S7- Chromatin state and GO enrichment analyses for DEG and DER clusters. (A) Heat map showing fold enrichment for each chromatin state of DE repeat and gene clusters shown on Fig. 3G and Fig. 4E. Increased color darkness represents increased enrichment. **(B)** GO enrichment analysis of biological process terms for the common downregulated genes in activator clones. The number of genes by term is shown on the x-axis, circle size indicates fold enrichment and darker colors indicate higher significance based on Fisher's Exact Test; null hypothesis: the proportion of observed genes in the analyzed list is not different from the background. **(C)** GO enrichment analysis of cellular component terms for the genes in cluster 2. **(D)** GO enrichment analysis of cellular component terms for the genes in cluster 3. For C and D, plot annotations are as described for panel B. **(E)** Box plot showing the distribution of average expression of genes that are part of the enriched GO biological process terms or KEGG pathways (shown on Fig. 6C) and the rest of genes classified as upregulated in the activator clones. Box plots show the 25th, 50th and 75th percentiles of fold changes. The inter-quantile range (IQR) is defined as the difference between the 75th and 25th percentiles. The upper whisker extends to the largest value within 1.5 x IQR above the third quartile, and the lower whisker extends to the smallest value within 1.5 x IQR below the first quartile. The notches indicate a 95% confidence interval around the median. P-value from two-sided Wilcoxon rank-sum testing the significance of the observed difference in distributions is displayed above the boxes; null hypothesis: the compared distributions differ by a location shift of zero. **(F)** Plot showing the percentage of upregulated genes with FL-L1MdTf elements (>6kb) among neuronal-related GO terms versus all other GO terms (from Fig. 6C and Appendix Fig. S7C,D). P-value from two-tailed unpaired t-test (panel F); null hypothesis: there is no difference in the percentage of genes containing L1MdTf belonging to neuronal-related terms and that of other GO terms.
