# Supplementary material for: Transcriptional perturbation of LINE-1 elements reveals their cis-regulatory potential
Source: EMBO Rep. 2026 Jul 8;27(16):4824–54. doi: 10.1038/s44319-026-00854-w (PMC13503937; doi:10.1038/s44319-026-00854-w)
Supplement: Supplementary file 2 [file 44319_2026_854_MOESM22_ESM.pdf]

Expanded View Figures

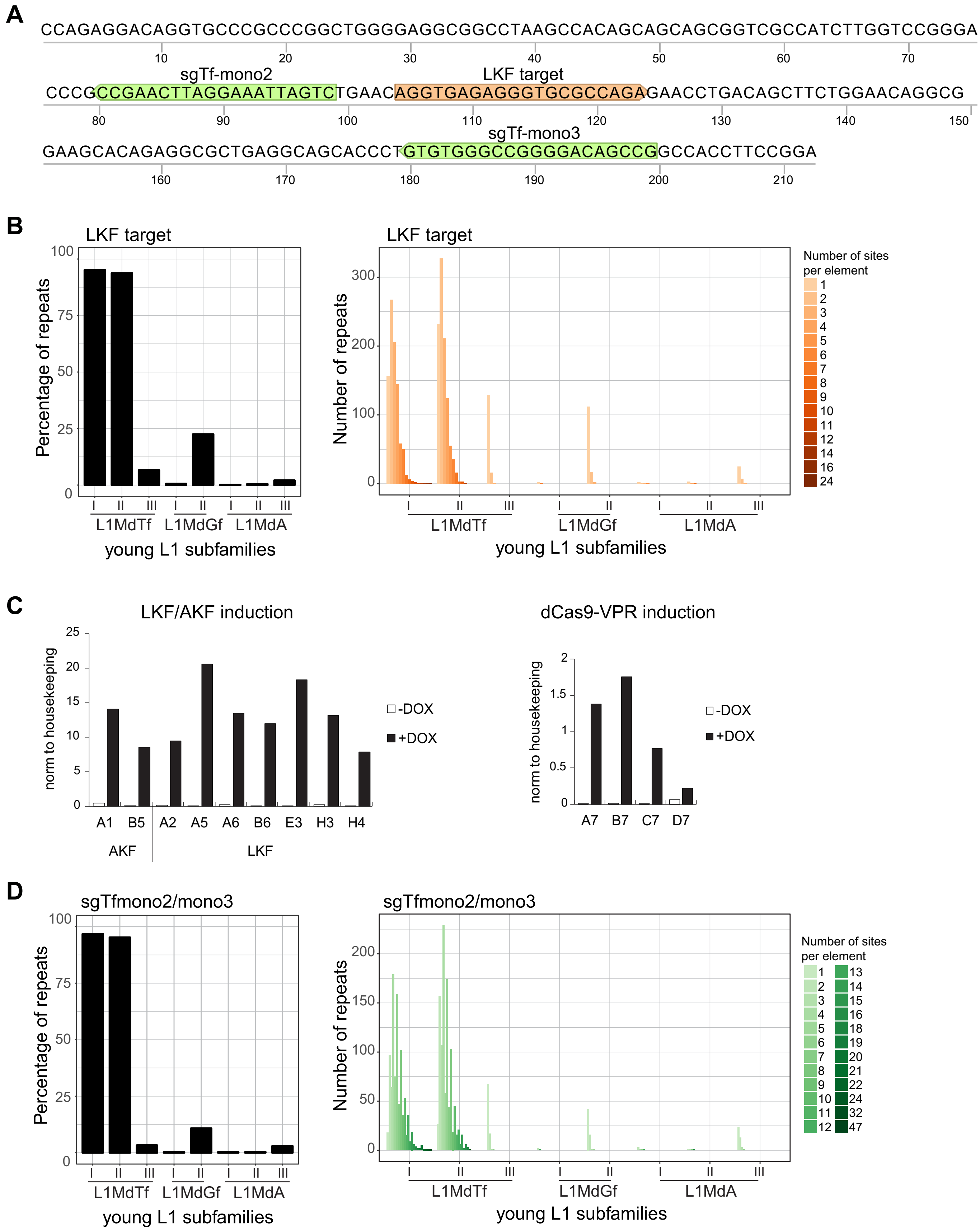

 © The Author(s)

◀ **Figure EV1. Identity and genomic representation of L1MdTf-repressor and activator predicted target sites.**

(**A**) Positions of LKF and sgRNAs (Tf-mono2 and Tf-mono3) target sequences within the 212 bp L1MdTf monomer consensus sequence. (**B**) Estimation of the percentage of young L1Md subfamilies bound by LKF repressor (left). Predicted number of elements from young L1Md subfamilies bound by LKF repressor according to the number of sites per element (right). (**C**) Following puromycin selection, colony picking and genotyping, 9 clones were analysed upon DOX treatment (48 h) by qPCR for expression of the LKF/AKF transgenes (left) and 4 clones for the expression of the dCas9-VPR transgene (right). (**D**) Estimation of the percentage of young L1Md subfamilies targeted by Tfmono2/3 guide RNAs (left). Number of elements from young L1Md subfamilies targeted by Tfmono2/3 according to the number of sites per element (right).

© The Author(s)

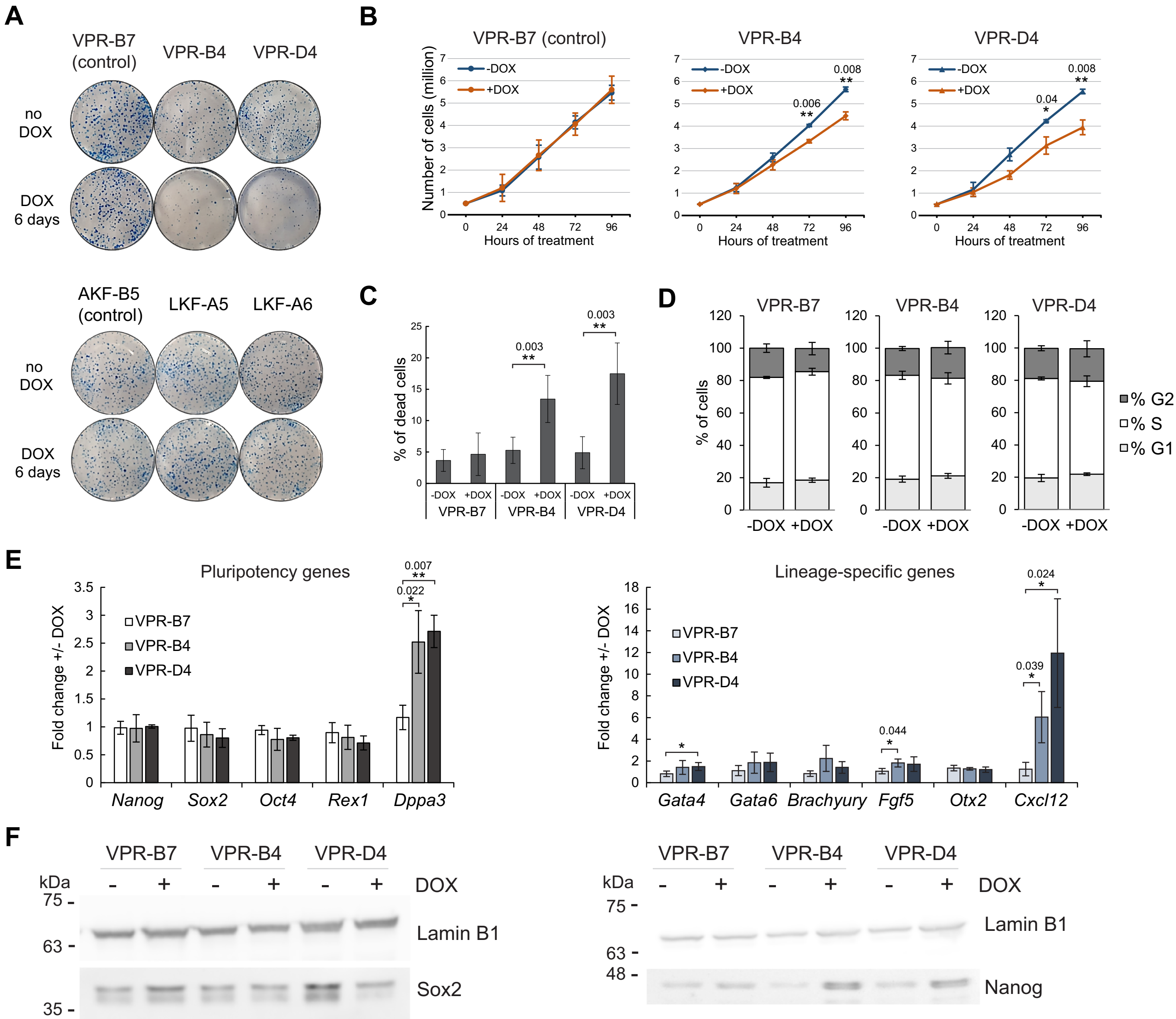

Figure EV2. Characterization of ESCs with perturbed L1MdTf expression, including analyses of proliferation, cell death, cell cycle progression, and marker expression.

(**A**) Representative images of colony formation assays in ESCs treated with or without DOX for 6 days, shown for both repressor and activator clones, as well as their respective controls. Colonies are marked by methylene blue stain. (**B**) Proliferation assay of VPR-B4, VPR-D4 and control clone, over 4 days with or without DOX treatment. Cells were counted every day. Data are shown as mean ± s.e.m. ($n = 3$ biological replicates). (**C**) Percentage of cell death calculated by counting Trypan blue-positive cells in VPR clones treated with or without DOX, every 24 h for 4 days. Data are presented as mean ± s.e.m. ($n = 7$). (**D**) Cell cycle analysis by flow cytometry using Propidium iodide staining, following 48 h of DOX treatment or no treatment in activator clones. Data are shown as mean ± s.e.m. ($n = 3$ biological replicates). (**E**) qPCR analysis of pluripotency and differentiation markers in untreated and DOX-treated (48 h) VPR-B7, VPR-B4 and VPR-D4 clones. Data are shown as mean ± s.e.m. ($n = 3$ or 4 biological replicates). (**F**) Western blot analysis showing no change in Sox2 and Nanog levels in activator clones VPR-B4 and VPR-D4, after DOX treatment for 48 h. LaminB1 is used as loading control. This experiment was repeated twice with similar results. Statistics: two-tailed paired (**B**, **C**, **E**) *t* test: *$P < 0.05$, **$P < 0.01$; null hypothesis: there is no difference in cell proliferation, cell death and expression levels between −DOX and +DOX conditions.

 © The Author(s)

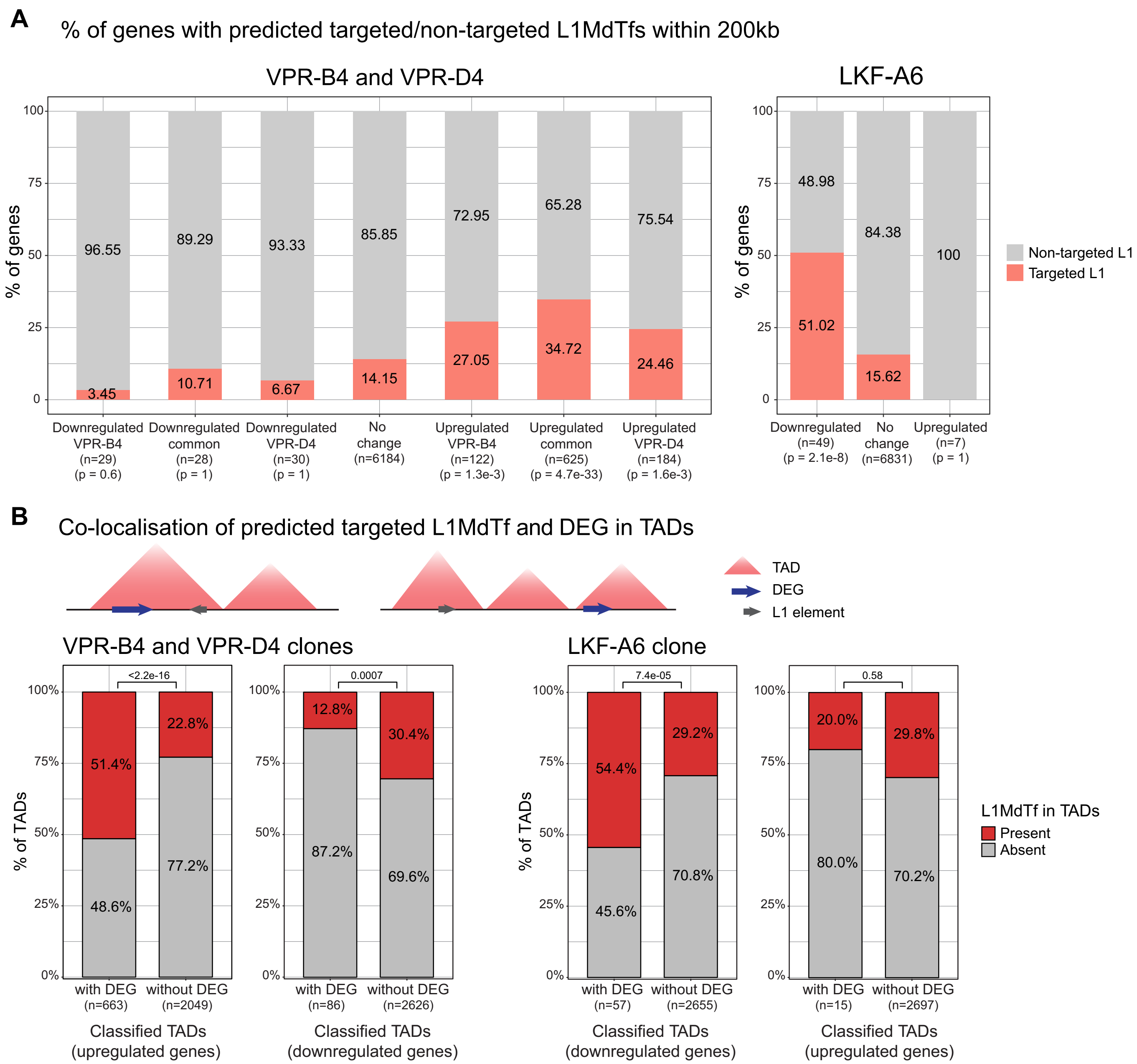

Figure EV3. Impact of predicted targeted L1MdTf on gene expression and TAD co-localisation.

(**A**) Percentages of genes (up-, downregulated, or with no change) with predicted targeted (red) versus non-targeted (grey) L1MdTfs within 200 kb of their vicinity in VPR-B4, VPR-D4 and LKF-A6 clones. The number of genes considered for each category and clone are indicated. *P* values were calculated using Fisher's exact test, with the "No change" category as reference, followed by Bonferroni correction for multiple comparisons; null hypothesis: transcriptional status is independent of proximity to predicted targeted/non-targeted L1MdTfs. (**B**) Proportion of predicted targeted L1MdTf elements located in the same TAD as upregulated or downregulated genes in VPR-B4/VPR-D4 (left) and in LKF-A6 (right) clones. The number of TADs considered for each category and clone are indicated. *P* values above the histograms were calculated using Chi-squared tests of independence (with Yates' continuity correction) to assess associations between L1MdTf presence/absence and DEG presence/absence in TADs; null hypothesis: no association between TAD annotations based on DEGs and L1MdTfs with predicted binding sites. The top diagram illustrates TAD annotation.

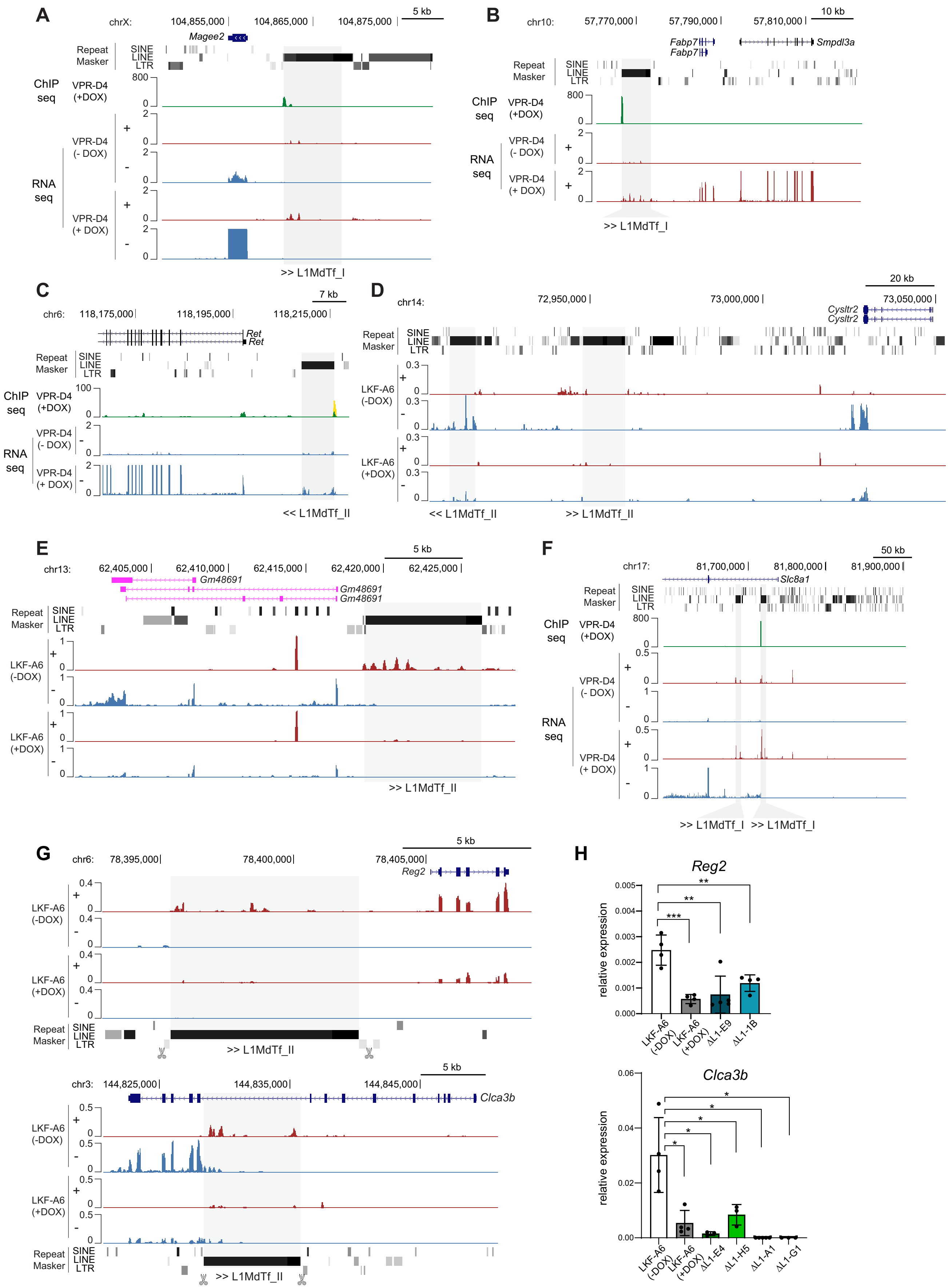
A
chrX: 104,855,000 104,865,000 104,875,000 5 kb
Magee2
Repeat Masker SINE LINE LTR
ChIP seq VPR-D4 (+DOX)
RNA seq VPR-D4 (- DOX) VPR-D4 (+ DOX)
>> L1MdTf_I
B
chr10: 57,770,000 57,790,000 57,810,000 10 kb
Fabp7 Fabp7 Smpdl3a
>> L1MdTf_I
C
chr6: 118,175,000 118,195,000 118,215,000 7 kb
Ret Ret
<< L1MdTf_II
D
chr14: 72,950,000 73,000,000 73,050,000 20 kb
Cysltr2 Cysltr2
LKF-A6 (-DOX) LKF-A6 (+DOX)
<< L1MdTf_II >> L1MdTf_II
E
chr13: 62,405,000 62,410,000 62,415,000 62,420,000 62,425,000 5 kb
Gm48691
>> L1MdTf_II
F
chr17: 81,700,000 81,800,000 81,900,000 50 kb
Slc8a1
>> L1MdTf_I >> L1MdTf_I
G
chr6: 78,395,000 78,400,000 78,405,000 5 kb
Reg2
>> L1MdTf_II
chr3: 144,825,000 144,835,000 144,845,000 5 kb
Clca3b
>> L1MdTf_II
H
Reg2
relative expression
LKF-A6 (-DOX) LKF-A6 (+DOX) ΔL1-E9 ΔL1-1B
Clca3b
LKF-A6 (-DOX) LKF-A6 (+DOX) ΔL1-E4 ΔL1-H5 ΔL1-A1 ΔL1-G1

 © The Author(s)

Figure EV4. Evidence of *cis*-regulation of genes by proximal L1MdTf repeats.

(**A**) *Magee2*, (**B**) *Fabp7/Smpdl3a* and (**C**) *Ret* gene vicinities showing increased expression upon L1MdTf activation. ChIP-seq for dCas9 (VPR-D4 clone, +DOX) profile is shown in green (uniquely mapped reads) and yellow (multimapped reads, not shown to scale). RNA-seq profiles by strand are shown in red (+ strand) and blue (− strand) for DOX-treated and untreated activator samples. Adjacent L1MdTf repeats with reciprocal expression change, and their orientation, are highlighted. (**D**) *Cysltr2* gene vicinity showing decreased expression upon L1MdTf LKF-mediated repression. Two adjacent L1MdTf elements, and their orientation, are highlighted. (**E**) *Gm48691* vicinity showing decreased expression upon adjacent L1MdTf repression as in (**D**). (**F**) *Slc8a1* vicinity showing increased expression upon L1MdTf activation. Intronic L1MdTfs with reciprocal changes and located in inverse orientation with respect to *Slc8a1* are highlighted. (**G**) *Reg2* (top) and *Clca3b* gene vicinities (bottom). RNA-seq profiles by strand (red (+ strand) and blue (− strand)) show downregulation of *Reg2* and *Clca3b* in LKF-A6 clone following DOX treatment. For both loci, L1MdTf elements are highlighted and the position of the sgRNAs used for CRISPR-mediated deletion are indicated with scissors. (**H**) qPCR analysis of *Reg2* (top) and *Clac3b* (bottom) in parental repressor clones LKF-A6 with or without 48 h of DOX treatment, compared to CRISPR-deleted (ΔL1) clones (2 independent clones for *Reg2*; 4 clones for *Clca3b*). Data are shown as mean ± s.e.m. ($n = 3$ to 5 biological replicates). Statistics: two-tailed unpaired *t* test (**H**): *$P < 0.05$, **$P < 0.01$, ***$P < 0.001$. Exact *P* values of significant differences shown from left to right: 0.0008, 0.0061, 0.0086 for *Reg2*; 0.0137, 0.0244, 0.0462, 0.0214, 0.0214 for *Clca3b*; null hypothesis: there is no difference in expression levels between −DOX and +DOX conditions, and between −DOX condition and ΔL1 clones.

© The Author(s)
