# Supplementary material for: Transcriptional perturbation of LINE-1 elements reveals their cis-regulatory potential
Source: EMBO Rep. 2026 Jul 8;27(16):4824–54. doi: 10.1038/s44319-026-00854-w (PMC13503937; doi:10.1038/s44319-026-00854-w)
Supplement: Supplementary file 3 [file 44319_2026_854_MOESM2_ESM.pdf]

EMBO reports

# Transcriptional perturbation of LINE-1 elements reveals their cis-regulatory potential

Yuvia Pérez-Rico, Anna Le Breton, Aurelie Bousard, Lenka Henao Misikova, Eskeatnaf Mulugeta, Sergio de Almeida, Alysson Muotri, Edith Heard, and Anne-Valerie Gendrel

*Corresponding author(s): Anne-Valerie Gendrel (anne-valerie.gendrel@gimm.pt)*



## Review Timeline:

| | |
|---|---|
| Transfer Date: | 12th Mar 26 |
| Editorial Decision: | 24th Apr 26 |
| Revision Received: | 12th Jun 26 |
| Accepted: | 18th Jun 26 |

*Editor: Esther Schnapp*

## Transaction Report: This manuscript was transferred to EMBO Reports following peer review at Review Commons.

(Note: With the exception of the correction of typographical or spelling errors that could be a source of ambiguity, letters and reports are not edited. Depending on transfer agreements, referee reports obtained elsewhere may or may not be included in this compilation. Referee reports are anonymous unless the Referee chooses to sign their reports.)

**Review #1**

**1. Evidence, reproducibility and clarity:**

**Evidence, reproducibility and clarity (Required)**

**Summary**

The authors utilize engineered transcriptional effectors (CRISPRa or KRAB-Zinc Finger proteins) to activate or inhibit the expression of retrotransposons in mouse embryonic stem cells. They focus on a young L1 family in mouse, L1MdTf, which is particularly active. This study reveals extensive alterations in gene expression for surrounding genes, and it suggests that the dysregulated genes are enriched in repressive chromatin and preferentially associated with neuronal functions.

**Major comments**

The experiments are well-executed and controlled overall. However, to fully support the conclusions, a few aspects of the work could be strengthened:

1. Engineered Effector Binding: The binding sites for the engineered effectors (CRISPRa or KRAB-Zinc Finger proteins) were predicted computationally, but not validated experimentally using techniques like ChIP-seq. Although mapping reads within L1MdTf might be challenging, ChIP-seq could still be informative. It would help define the ratio of on-target vs. off-target binding and elucidate potential off-target effects (binding outside L1MdTf repeats).
2. Mechanism of action: The study somehow assumes that the effectors influence L1MdTf transcription, which then impacts the host transcriptome. However, it is also possible that the engineered effectors directly modulate nearby cis-regulatory elements (promoters or enhancers) due to their proximity to L1MdTf sequences. Further investigation is needed to differentiate between these possibilities.
3. Statistics: The study lacks robust statistical testing in several figures (e.g., Fig. 1B,D; 2C,E; S2; S5C,D). Additionally, the null hypotheses and statistical models used are not explicitly defined. To strengthen the analysis, the authors could quantify the percentage of differentially expressed (DE) or allele-specifically expressed genes associated with targeted L1s compared to non-targeted L1s or other relevant control groups. Furthermore, neuronal genes are known to be longer than average, which can bias enrichment analyses like those performed using DAVID. This increased length makes them more likely to harbor L1 insertions and potentially be affected by the engineered effectors targeting these elements.

It would be informative to see how the conclusions change if the null models account for gene size or only consider genes with full-length L1MdTf elements.
4. Allele-specific expression: The analysis of allele-specific expression could be further strengthened by incorporating L1 heterozygosity data. Since the whole genome sequence of these cells is available, calling genotypes for L1 insertions using established pipelines like MELT or others would be a straightforward approach. This would allow the authors to ensure that allele-specific expression patterns are indeed associated with L1 insertional polymorphisms.

**Minor comments**

5. Western blot analysis could benefit from a more quantitative approach. Analyzing the intensity profiles of the ORF1p bands might help differentiate between potential ORF1p isoforms and pinpoint the specific changes (reduction or increase) in the top band. Additionally, an earlier explanation for the observed doublet band as a consequence of coexisting L1 families would be helpful for readers unfamiliar with mouse L1s.
6. The chromHMM enrichment analysis reports fold enrichments (ratios of observed vs. expected associations with chromatin states). However, the magnitude of these enrichments seems to be modest in most cases. It would be informative to see the actual number of sequences falling into each category for a better understanding of the enrichment patterns.

**2. Significance:**

**Significance (Required)**

*Strengths*

- This study contributes to a limited body of research exploring the consequences of the global activation or repression of entire transposable element families.
- Notably, the authors achieve this manipulation in a relevant cell type (embryonic stem cells) where retrotransposition is known to occur, utilizing a retrotransposition-competent L1 family (L1MdTf).

*Limitations*

- While the study offers a relatively novel approach, the biological question addressed could be further refined. This would allow for more specific conclusions regarding the role/consequences of L1MdTf dysregulation.

- Artificially targeting L1 sequences is conceptually similar with randomly targeting the genome for transcriptional effects, since many, possibly most, L1 elements may not be naturally expressed under any biological conditions.

While the manuscript confirms the known potential of L1 sequences to act as cis-regulatory elements, it does not provide significant new insights into the mechanisms underlying their regulatory potential. Notably, a recent study by Tong et al. IJMS (2024) demonstrated the activation of young human L1 elements using CRISPRa. This work similarly confirmed the ability of young human L1 elements to form chimeric transcripts and influence gene expression, likely through promoter or enhancer activity. However, key limitations of both studies are that the strength of the engineered effector might influence the observed transcriptional effects, and that the targeted L1 elements might not be naturally expressed, potentially leading to an overestimation of the regulatory impact of L1 activation.

The study focuses on the impact of L1MdTf on gene expression, but exploring the phenotypic consequences of manipulating its expression could significantly broaden its significance. This would provide valuable insights into the functional impact of L1 activity in a broader context, potentially attracting a wider audience. In this context, L1MdTf silencing in mESC cells which naturally express these elements could be more insightful than artificially over-activating them. Phenotypic analyses could encompass cell cycle progression, commitment to the primed pluripotent state, totipotency, differentiation potential, DNA damage response, or X chromosome inactivation. Identifying whether observed phenotypes arise from cis-effects (on neighboring genes) or trans-effects (mediated by L1 RNA or proteins) would be crucial for understanding the underlying mechanisms.

My field of expertise lies in molecular and cellular biology and genomics, with a particular focus on transposable elements.

**3. How much time do you estimate the authors will need to complete the suggested revisions:**

**Estimated time to Complete Revisions (Required)**

**(Decision Recommendation)**

Between 3 and 6 months

**4. *Review Commons* values the work of reviewers and encourages them to get credit for their work. Select 'Yes' below to register your reviewing activity at [Web of Science Reviewer Recognition Service](#) (formerly Publons); note that the content of your review will not be visible on Web of Science.**

**Web of Science Reviewer Recognition**

Yes

**Review #2**

**1. Evidence, reproducibility and clarity:**

**Evidence, reproducibility and clarity (Required)**

In this study Pérez-Rico et al. set out to study the cis-regulatory effects of evolutionary young L1 elements (specifically L1MdTf) in mouse embryonic stem cells. Using state of the art systems, they manage to silence/activate these elements expression and study the following gene expression cascade.

**Major comments:**

1. A general assessment or profiling of the L1MdTf expression in WT mESC is missing. It is very challenging to set their results in perspective (especially those of the repression system) without a general overview of the baseline L1-expression in mESC. How many elements does the WT mESC express? How is the genomic distribution of these elements? And how does it relate to nearby gene expression?
2. Off-target effects are very poorly documented and limited to in silico analysis. Documentation of changes in H3K4me3 and H3K9me3 upon activation/silencing would be beneficial to interpret the results. As it is now it is very difficult to understand why the activation-system is so much stronger that the silencing system.
3. It would be beneficial to explain why different experimental strategies were used for silencing/activation?
4. The study would benefit of a more detailed characterization of their systems' effect. E.g. It is not clear from Figure 3D-E how many of these repeats are part of the L1MdTf subfamily or other closely related subfamilies. This is relevant to assess the specificity of the systems.
5. The RNA-seq analysis relies on reads that are too short to uniquely map most L1s in the

mouse genome. This should be mentioned and discussed.
6. A key issue that remains unresolved is the functional of L1-silencing/activation in mESC. Does this change cell morphology, pluripotency, or differentiation potential? This is essential information.
7. Fig 3B: Is this n=2? Hence why there is no statistical analyses? Same for Fig 5A-B: here a p-value is presented but it would be good to highlight the statistically significant results somehow.
8. Are only L1MdTf elements plotted in Fig 3D and 3E? As the authors claim that the system is quite specific, it would be good to have a clear view of the repeats that are differentially expressed and are evolutionarily close to the L1MdTf family. The figure might also benefit of some further characterization on a family level (multi-mapping) rather than single elements (unique mapping or multi mapping with reads that map to a single location, like here)
9. The authors provide some evidence for the existence of a bidirectional promoter on L1MdTf elements. In what elements was this observed? Does it result in chimeric transcripts? And if so, are these genes in line with the enrichment found?

**Minor comments:**

1. The introduction would benefit from a more detailed description of the L1-content in the mouse genome including the presence of polymorphic elements.
2. There is no characterization of the edited mESC. Stainings? Markers from bulk RNA seq analysis? Morphology assessment?

**2. Significance:**

**Significance (Required)**

The manuscript is timely and in line to some similar and very recent studies suggesting a link between evolutionary young L1s and neuronal-related genes in mice (e.g. Toda et al.). However, the manuscript is quite preliminary in the current form. The investigation of potential off-target effects of their system requires more in-depth investigations. In addition, the manuscript could be improved with further exploration into the functional consequences of L1-silencing/activation.

**3. How much time do you estimate the authors will need to complete the suggested revisions:**

**Estimated time to Complete Revisions (Required)**

**(Decision Recommendation)**

Between 3 and 6 months

**4. *Review Commons* values the work of reviewers and encourages them to get credit for their work. Select 'Yes' below to register your reviewing activity at [Web of Science Reviewer Recognition Service]() (formerly Publons); note that the content of your review will not be visible on Web of Science.**

**Web of Science Reviewer Recognition**

No

**Review #3**

**1. Evidence, reproducibility and clarity:**

**Evidence, reproducibility and clarity (Required)**

Perez-Rico et al use dCAS9 to activate and repress L1 expression levels in the subfamily level for younger mouse L1s to address their cisregulatory potential

They choose to transcriptionally perturb full-length L1s subfamily.

An important topic, and I liked the approach of using CRISPR to perturb an entire subfamily of transposable elements. However, this study has yet to add any new findings or conclusions. This study can be improved by further analysis or experimentation. The regulatory role of L1s in cis-regulatory function has been previously demonstrated in mouse and human stem cell systems using multiple approaches, including CRISPR-mediated perturbations, CRISPR-based deletions, and 3D chromatin structure analysis. My main concern is the analysis is very superficial and lacks integration of other datatypes such as HiC to further support the role of L1s in enhancer activity and further validation on candidate loci.
One of the major limitations of CRISPR-based perturbations is that it can be difficult to conclude the cis-regulatory role of these targeted elements (L1) as synthetic tethering of VPR or KRAB domain can indirectly spread their effect in cis, which otherwise may not have a specific cis-regulatory role to regulates genes in cis.
Some of the major comments that needs to be addressed to strengthen the manuscript conclusions

1. One of the important controls could be targeting different or older L1s to show how it can affect gene expression. can authors perturb older families?
2. Although meta-analysis of L1s show up/downregulation of L1MdTf,
ChIPseq for dCAS9 is needed to demonstrate which L1s are directly targeted in the experiment and analyse the data separately for dCAS9 bound L1s vs rest of the L1s in the subfamily.
3. L1s' transcriptional activation could be independent of their cis-regulatory activity. So, it is important to analyse the enhancer chromatin feature of TEs: Do they make 3D contact with the nearby genes?
4. how many replicates of RNA seq is done?
5. Are genes that respond to CRISPR activation in the same TAD? Do they loop with LINE1?
6. Figures 4 A &B show clear perturbation of host genes upon CRISPRi. However, they need to show L1s outside the genes (not in the introns) as CRISPR dCAS9 perturbation of Intronic L1s is expected to affect the expression of genes.
7. One of the key experiment missing is to genetically delete candidate L1s that are intergenic that are predicted to have a cis-regulatory function in the RNAseq data.
8. Authors should present the RNAseq data for genes 10 to 100 kb away from dCAS9-KRAB and dCAS9-VPR targeted L1s. It will be interesting to compare KRAB v VPR effect with distance.
9. The suggested role of L1s in neuronal gene expression is not strongly supported by their experiments or anlaysis.
The authors end the results section with "Most of these genes are not expressed or have low expression levels in ESCs compared to other upregulated genes that are not part of these processes and pathways (Fig.S9E)". This result suggests that dCAS9-VPR targeting to multiple (How many are targeted is missing) L1s leads to the activation of many genes that are not normally expressed in mESCs due to the spreading of transcriptional activators or mESCs getting spontaneously differentiated to the neuronal lineage. Authors need to address these points.

**Minor comments**

Fig 2E) which housekeeping gene

Figures 4 A &B, show if these L1s make looping interactions or are in the same TAD as target genes.

**Referee Cross-commenting**

I agree with the other two reviewers points.

**2. Significance:**

**Significance (Required)**

This study can be improved by further analysis or experimentation. In its current version, the conceptual advance offered by the current results and approach of the manuscript seem limited.

**3. How much time do you estimate the authors will need to complete the suggested revisions:**

**Estimated time to Complete Revisions (Required)**

**(Decision Recommendation)**

Between 3 and 6 months

**4. *Review Commons* values the work of reviewers and encourages them to get credit for their work. Select 'Yes' below to register your reviewing activity at Web of Science Reviewer Recognition Service (formerly Publons); note that the content of your review will not be visible on Web of Science.**

**Web of Science Reviewer Recognition**

Yes

**Perez-Rico et al.**

We thank the editor and the reviewers for their positive and constructive comments. Below is our point-by-point responses.

---------------------------------------------------------------------------

**Reviewer #1 (Evidence, reproducibility and clarity (Required)):**

Summary

The authors utilize engineered transcriptional effectors (CRISPRa or KRAB-Zinc Finger proteins) to activate or inhibit the expression of retrotransposons in mouse embryonic stem cells. They focus on a young L1 family in mouse, L1MdTf, which is particularly active. This study reveals extensive alterations in gene expression for surrounding genes, and it suggests that the dysregulated genes are enriched in repressive chromatin and preferentially associated with neuronal functions.

Major comments

The experiments are well-executed and controlled overall. However, to fully support the conclusions, a few aspects of the work could be strengthened:

1. Engineered Effector Binding: The binding sites for the engineered effectors (CRISPRa or KRAB-Zinc Finger proteins) were predicted computationally, but not validated experimentally using techniques like ChIP-seq. Although mapping reads within L1MdTf might be challenging, ChIP-seq could still be informative. It would help define the ratio of on-target vs. off-target binding and elucidate potential off-target effects (binding outside L1MdTf repeats).

We thank the reviewer for this suggestion and agree that this is an important control. We have now performed ChIP-seq for dCas9 in the VPR control (B7; +DOX condition only) clone and in the activation VPR clones (B4 & D4; -DOX and +DOX conditions). A similar experiment could not be performed in the repression clones, because of the lack of suitable antibody for the LKF/AKF effectors. The ChIP-seq data, presented in **Fig.3B,C** shows very specific targeting of the dCas9-VPR effector towards L1MdTfs, mainly from the L1MdTf_I and L1MdTf_II subfamilies, in agreement with our in silico analysis of the gRNAs targets (Fig.S1). We found very few off-target binding sites, at repeats of other L1 subfamilies and 3 genes, one of which was common between the two clones (**Table S3**).

2. Mechanism of action: The study somehow assumes that the effectors influence L1MdTf transcription, which then impacts the host transcriptome. However, it is also possible that the engineered effectors directly modulate nearby cis-regulatory elements (promoters or enhancers) due to their proximity to L1MdTf sequences. Further investigation is needed to differentiate between these possibilities.

We agree with the reviewer's concern about the mechanism of action. In the discussion section, we consider and discuss the different cis- and trans-mechanisms through which L1 could impact host gene regulation, based on previous reports and on our data. In addition, to rule out direct regulation mediated by the engineered effectors in cases where L1MdTfs are located within or very close to genes, we have now included an experiment where we have performed deletions of two L1MdTf elements that are

proximal to two DEGs (*Clca3b* and *Reg2*) in the repressor clone LKF-A6. We then tested the impact of these deletions on expression of the adjacent genes (shown in **Fig.S9G,H**). In both cases, we were able to reproduce the effect observed upon DOX induction, e.g. decreased expression of the genes upon L1MdTf deletions, indicating in these two examples direct cis-regulation by the proximal L1MdTf elements. Furthermore, ChIP-seq/RNA-seq data of the activator clones show that proximal binding of the dCas9-VPR effector and subsequent L1MdTf upregulation is not sufficient to induce expression of nearby genes (cf. examples of *Fsip2*, *A930005H10Rik* & *Dph5* genes in **Fig.S10A**).

3. Statistics: The study lacks robust statistical testing in several figures (e.g., Fig. 1B,D; 2C,E; S2; S5C,D).

We thank the reviewer for highlighting the need for additional statistics. The data shown in **Fig.1B,D** corresponds to an RT-qPCR experiment from single cDNA samples per clone analysed (technical replicates only) as a preliminary screen to identify candidate clones, rather than a statistical comparison of clones AKF vs LKF. We have then included the results of one-sample t-tests to determine if the differences in fold changes of LKF clones are significantly different from the expected value of 1.

Furthermore, we have now included results of statistical tests (p-values of paired/unpaired t-tests) for the subsequent characterization/comparisons by qPCR of the selected clones as shown in **Fig.2C,E** and **Fig.S6C,D** (former Fig.S5), and the microscopy image analysis in **Fig.S2**.

Additionally, the null hypotheses and statistical models used are not explicitly defined.

The statistical models used for each experiment/test are indicated in the corresponding figure legend and in the methods section (e.g. qPCR, distance analysis, RNA FISH, IF, proliferation assays). We have also explicitly included the null hypothesis of each test in the legend or methods section.

To strengthen the analysis, the authors could quantify the percentage of differentially expressed (DE) or allele-specifically expressed genes associated with targeted L1s compared to non-targeted L1s or other relevant control groups.

To strengthen our analysis on the distance between DEGs and L1s, we have now included the analysis suggested by the reviewer. We calculated the percentage of DEGs whose closest L1MdTf, within a 200 kb window of their gene body, is a targeted or non-targeted element. We found, for example, that out of the 625 common VPR-upregulated genes with an L1MdTF located within 200kb, 34.72% (217 genes) have a targeted L1MdTf within this window, whereas 65.28% are associated with untargeted L1MdTf elements within the same range. In spite of the apparent low percentage, 34.72% was higher than the percentages observed for downregulated genes or genes without significant change in expression (shown in **Fig.S8A**). The same bias in association of DEGs and targeted L1MdTfs was observed in the LKF clone, in this case for downregulated genes.

Furthermore, neuronal genes are known to be longer than average, which can bias enrichment analyses like those performed using DAVID. This increased length makes them more likely to harbor L1 insertions and potentially be affected by the engineered effectors targeting these elements. It would be informative to see how the conclusions change if the null models account for gene size or only consider genes with full-length L1MdTf elements.

We thank the reviewer and directly address both suggested null models. In our latest analyses we observed that neuronal genes' greater length can indeed bias the GO enrichments and increase L1 insertion probability. Specifically, we performed length-adjusted analyses by GOseq and GOglm, which eliminated all GO enrichments, and we calculated the percentage of genes containing FL-L1MdTf elements (>6kb) for each GO term from **Fig.6** and **Fig.S11** (former Fig.S9), which showed that the percentage of genes containing FL-L1MdTf elements in GO neuronal terms is significantly higher than for other GO and KEGG pathway terms. The latter highlights the increased susceptibility of neuronal genes to transcriptional interference or misregulation of L1 elements, notably in cellular contexts where these genes are not necessarily expressed, such as ESCs in this study. As suggested by the reviewer, we also performed L1-focused GO analysis by only taking into consideration genes with intronic FL-L1s, and retrieved significant enrichment of neuronal terms exclusively (5 terms with adjusted p-value < 0.01). Therefore, while gene length influences the raw enrichments, L1MdTf-containing neuronal genes show specific susceptibility to VPR modulation beyond RNA-seq size effects alone. The relevant relationship between L1s and neuronal genes is further supported by the fact that the only chromatin loop interactions detected between DEGs and L1MdTfs match genes with neuronal functions. Of note, in contrast to RNA-seq counts that can be influenced by gene length, Hi-C data used to detect loops was counted by dividing the genome in 5 kb bins, therefore, neuronal genes will not be preferentially detected simply by their greater length. Finally, while we cannot discard the possibility of the engineered effectors directly affecting the expression for all DEGs, we have now included evidence of binding confirmed by ChIP-seq without impact on the bound/nearby gene in the revised version.

4. Allele-specific expression: The analysis of allele-specific expression could be further strengthened by incorporating L1 heterozygosity data. Since the whole genome sequence of these cells is available, calling genotypes for L1 insertions using established pipelines like MELT or others would be a straightforward approach. This would allow the authors to ensure that allele-specific expression patterns are indeed associated with L1 insertional polymorphisms.

We agree that incorporating L1 heterozygosity data would enhance the allele-specific expression analysis. However, the whole genome sequence data in our study is of lower coverage (23x) than the typical minimum of 30x recommended for reliable and comprehensive detection of L1 insertion polymorphisms using established pipelines like MELT.

Minor comments

5. Western blot analysis could benefit from a more quantitative approach. Analyzing the intensity profiles of the ORF1p bands might help differentiate between potential ORF1p isoforms and pinpoint the specific changes (reduction or increase) in the top band. Additionally, an earlier explanation for the observed doublet band as a consequence of coexisting L1 families would be helpful for readers unfamiliar with mouse L1s.

We have now quantified the ORF1p signal in non-treated and DOX-treated samples, by normalizing to the LaminB1 signal of our Western blot analyses for both the repressor (Fig.2D) and activator clones (Fig.2F). This quantification, shown on **Fig.S2A**, accounts for both ORF1p isoforms and highlights the specific changes- either a reduction or an increase- observed for the upper band (which corresponds to the L1MdTfs ORF1 isoform). In addition, we added further explanation for the observed doublet band

corresponding to ORF1p isoforms in the relevant result section (see "*Characterization of ESC lines encoding engineered L1MdTf-repressor and activator*").

6. The chromHMM enrichment analysis reports fold enrichments (ratios of observed vs. expected associations with chromatin states). However, the magnitude of these enrichments seems to be modest in most cases. It would be informative to see the actual number of sequences falling into each category for a better understanding of the enrichment patterns.

We now provide a supplementary table (**Table S19**) with the actual number of fragments falling in each chromHMM category and for the different categories of DEGs and DERs in both systems (corresponding to Fig.6A and Fig.S11A).

Reviewer #1 (Significance (Required)):

Strengths
- This study contributes to a limited body of research exploring the consequences of the global activation or repression of entire transposable element families.
- Notably, the authors achieve this manipulation in a relevant cell type (embryonic stem cells) where retrotransposition is known to occur, utilizing a retrotransposition-competent L1 family (L1MdTf).

Limitations
- While the study offers a relatively novel approach, the biological question addressed could be further refined. This would allow for more specific conclusions regarding the role/consequences of L1MdTf dysregulation.
- Artificially targeting L1 sequences is conceptually similar with randomly targeting the genome for transcriptional effects, since many, possibly most, L1 elements may not be naturally expressed under any biological conditions.

We appreciate these points and have refined the text to better reflect the data. Our main objective was to target the promoter of full length young L1MdTfs, which have high transcriptional activity potential due to evolutionary youth. In contrast, randomly targeting the genome or older, transcriptionally inert repeat families (related to point #1 of reviewer 3) would primarily hit sequences lacking this expression competence. While most L1MdTfs are silent in ESCs, their demonstrated expression in other contexts (and our observed activation upon dCas9-VPR targeting) indicates that they may possess specific features enabling conditional activity. Furthermore, the key distinctions from random targeting include: 1) as confirmed by the ChIP-seq data, dCas9-VPR binds targeted L1MdTf promoters, not randomly; 2) proximal binding is insufficient for local activation to occur as exemplified by *Fsip2*, *A930005H10Rik* and *Dph5* genes (**Fig.S10A**).

While the manuscript confirms the known potential of L1 sequences to act as cis-regulatory elements, it does not provide significant new insights into the mechanisms underlying their regulatory potential. Notably, a recent study by Tong et al. IJMS (2024) demonstrated the activation of young human L1

elements using CRISPRa. This work similarly confirmed the ability of young human L1 elements to form chimeric transcripts and influence gene expression, likely through promoter or enhancer activity. However, key limitations of both studies are that the strength of the engineered effector might influence the observed transcriptional effects, and that the targeted L1 elements might not be naturally expressed, potentially leading to an overestimation of the regulatory impact of L1 activation.

The study focuses on the impact of L1MdTf on gene expression, but exploring the phenotypic consequences of manipulating its expression could significantly broaden its significance. This would provide valuable insights into the functional impact of L1 activity in a broader context, potentially attracting a wider audience. In this context, L1MdTf silencing in mESC cells which naturally express these elements could be more insightful than artificially over-activating them. Phenotypic analyses could encompass cell cycle progression, commitment to the primed pluripotent state, totipotency, differentiation potential, DNA damage response, or X chromosome inactivation. Identifying whether observed phenotypes arise from cis-effects (on neighboring genes) or trans-effects (mediated by L1 RNA or proteins) would be crucial for understanding the underlying mechanisms.

We thank the reviewer for their input about the potential significance of our work.

In this study, we decided to focus our analyses more on the consequences of L1MdTf upregulation than their downregulation, given the moderate impact observed on gene expression upon L1 downregulation.

In the revised version, we provide new results about the phenotypic consequences of L1MdTf expression perturbation in undifferentiated ES cells, notably the impact on cell proliferation, cell death, cell cycle, pluripotency and DNA damage (shown in **Fig.S2D**; **Fig.S3**). The results obtained show that L1MdTf upregulation, but not L1MdTf downregulation, have a significant impact on cell proliferation/cell death and in the formation and size of ESC colonies. Besides this, we did not observe any impact on cell cycle progression and DNA double strand breaks levels. We also quantified changes in gene expression for a number of genes associated with pluripotency and lineage-specification. This analysis revealed that the expression of pluripotency and differentiation markers is only mildly affected.

The consequences of L1MdTf expression perturbation upon differentiation of ESCs, particularly in relation to X-chromosome inactivation and neuronal-lineage commitment, are beyond the scope of this manuscript and will be the focus of future studies.

My field of expertise lies in molecular and cellular biology and genomics, with a particular focus on transposable elements.

**Reviewer #2 (Evidence, reproducibility and clarity (Required)):**

In this study Pérez-Rico et al. set out to study the cis-regulatory effects of evolutionary young L1 elements (specifically L1MdTf) in mouse embryonic stem cells. Using state of the art systems, they manage to silence/activate these elements expression and study the following gene expression cascade.

Major comments:

1. A general assessment or profiling of the L1MdTf expression in WT mESC is missing. It is very challenging to set their results in perspective (especially those of the repression system) without a general overview of the baseline L1-expression in mESC. How many elements does the WT mESC express? How is the genomic distribution of these elements? And how does it relate to nearby gene expression?

This is a very interesting point raised by the reviewer and a complex question to address.

We have assessed the baseline expression of L1MdTf elements in WT mESCs using our RNA-seq data (in -DOX conditions) and found that between 800 and 1,500 L1MdTf elements (considering L1MdTf_I, _II, and _III sub-families) are expressed, as shown in the following plot (duplicates are displayed for each clone).

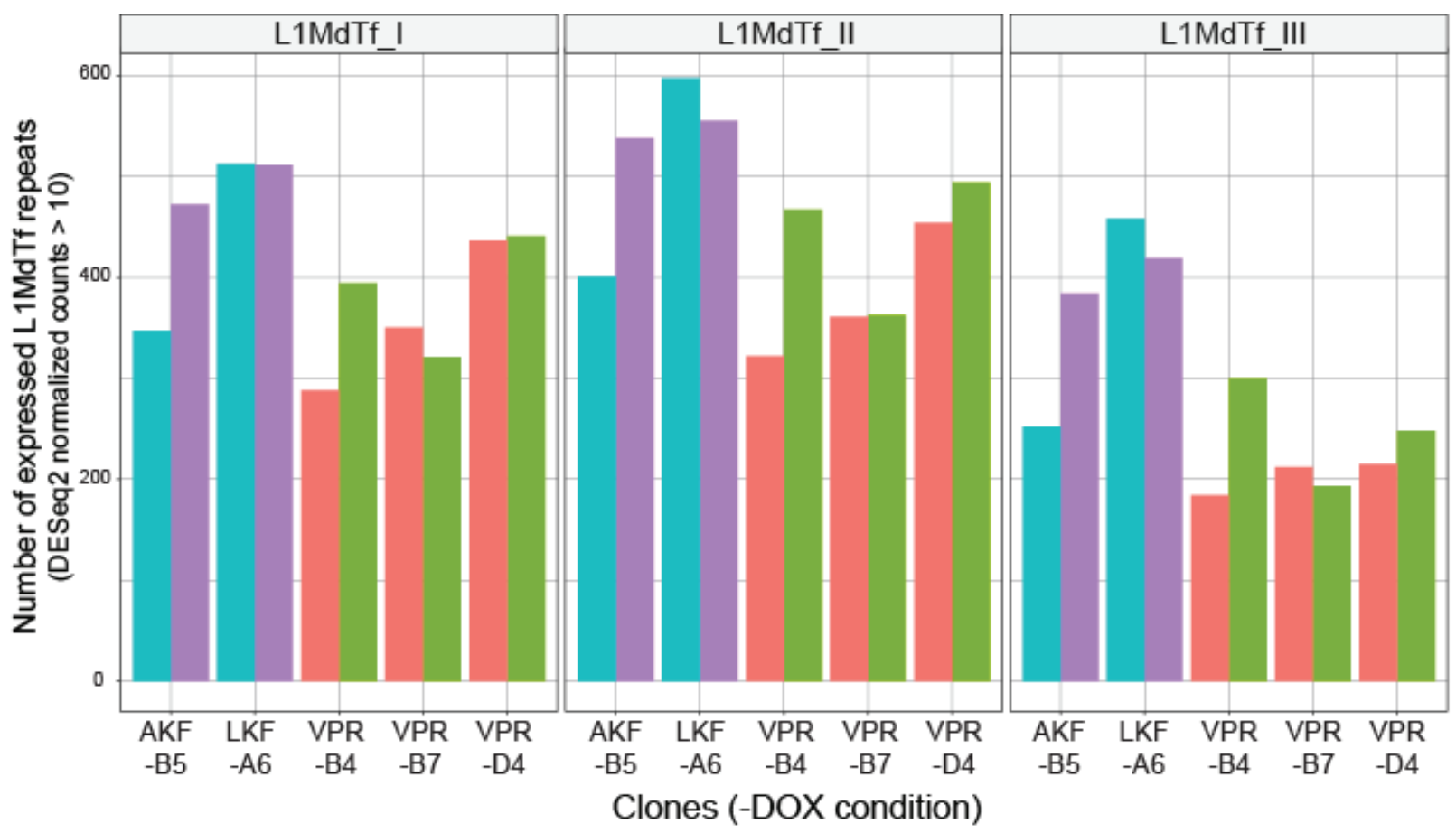


This range corresponds to approximately 15% to 30% of all L1MdTf elements in the genome. For this calculation, we only included elements with a DESeq2 normalized expression value greater than 10, chosen to match the minimum expression of elements showing significant changes in the differential expression analysis. Consistent with our differential expression analysis, these expressed L1MdTf elements are distributed broadly across the entire genome. We decided not to include this plot in the revised version, as the added value would be limited and the data can be calculated from the supplementary tables, but included a sentence referring to the mentioned values: "*In the absence of DOX treatment, we detected a basal expression of 800 to 1,500 L1MdTf elements across L1MdTf_I, _II, and _III subfamilies (Table S6-S8)*."

In addition, determining a general, global relationship between L1MdTf expression and nearby gene expression is challenging. However, we now provide specific cases that highlight a potential impact. The *Iqub* gene example (**Fig.4A**; **Fig.S10B**) highlights that the intronic L1MdTf_II element, expressed in -DOX conditions, partly impacts *Iqub* expression. In addition, the deletion experiment of two L1MdTfs (**Fig.S9G,H**), performed in the LKF-A6 clone, suggests that *Reg2* and *Clca3b* genes may be subject to endogenous regulation by the nearby L1, in the absence of any experimental perturbation.

2. Off-target effects are very poorly documented and limited to in silico analysis. Documentation of changes in H3K4me3 and H3K9me3 upon activation/silencing would be beneficial to interpret the results. As it is now it is very difficult to understand why the activation-system is so much stronger that the silencing system.

To document off targets experimentally, we provide ChIP-seq data for dCas9 in the CRISPRa (dCas9-VPR) clones. Unfortunately, we could not provide similar data for the repressor cell lines due to lack of suitable antibody. However, we found that the vast majority of dCas9 ChIP-seq peaks overlap with L1MdTf elements and that the number of off-targets are minimal, overlapping a few other L1 repeat types and only 3 genes, one of which was common between the two clones (**Table S3**). Remarkably, the ChIP-seq peaks largely corroborate the targets identified through our initial in silico analysis (**Fig.3C**).

The strength differences observed for the activation system compared to the repression system relate to the impact on gene expression and not to the efficiency of L1MdTf modulation itself, as both systems appear equally efficient at modulating the expression of the targeted L1MdTf elements (cf. Fig.2C to F). However, the impact on gene expression is much more pronounced after L1MdTf upregulation, likely due to the broader dynamic range of upregulation versus downregulation of L1MdTfs. If a L1 element is low or moderately expressed, the possible downregulation is consequently mild compared to the level of upregulation that can be achieved from low or even no expression. With this context, adding data for active or repressive histone marks upon system activation or silencing would not be beneficial for interpreting these results, nor provide further mechanistic insights, as both VPR and KRAB domains are known to robustly modulate their respective histone marks and the differential effect on gene expression is a downstream consequence of the L1MdTf starting expression level.

3. It would be beneficial to explain why different experimental strategies were used for silencing/activation?

We understand the reviewer's comment regarding the use of different experimental systems. The Zinc Finger (ZF) system was obtained from a collaborator (Dr Alysson Muotri, an author in this manuscript) and available in the lab when this project started. Preliminary experiments comparing CRISPRi and the ZF system revealed that ZF were more effective at downregulating L1MdTf expression when analysing cell populations. Based on these results, we chose to proceed with the ZF approach. We have now included a sentence in the manuscript to clarify this.

4. The study would benefit of a more detailed characterization of their systems' effect. E.g. It is not clear from Figure 3D-E how many of these repeats are part of the L1MdTf subfamily or other closely related subfamilies. This is relevant to assess the specificity of the systems.

We have added a column to annotate L1MdTfs in these panels (now **Fig.3F-G**). Moreover, the specific information is provided on Table S6 (for repressor clone LKF-A6), Table S7 (for activator clone VPR-B4), and Table S8 (for activator clone VPR-D4). In addition, the numbers are indicated in the following sentence in the results section: "*Overall, using unique mapping, we found 513 individual repeat annotations differentially expressed (padj<0.01) in the LKF-expressing clone, with a substantial fraction (n=377 out of 1,023 elements tested) corresponding to repressed L1MdTf annotations (Table S6). In the VPR-B4 and -D4 clones, we found 1,400 and 1,421 repeat annotations with significant changes in expression (padj<0.01), respectively, of which a large part corresponds to upregulated L1MdTf elements (n=709 out of 2,315 for VPR-B4, Table S7; n=659 out of 1,815 for VPR-D4, Table S8). [...] By comparing the DERs in all clones, we identified 206 repeat annotations showing reciprocal expression changes mediated by the engineered effectors and corresponding mostly to L1MdTf elements (Fig.3F; Table S9)*".

5. The RNA-seq analysis relies on reads that are too short to uniquely map most L1s in the mouse genome. This should be mentioned and discussed.

Although our study relies on short reads (paired-end 2x150bp) to analyse gene and TE expression, we were able to identify uniquely mapped reads within L1 elements, including within elements from the youngest families, as shown also by other groups (He et al, 2019; Pal et al, 2023; Adami et al, 2025). Yet, this unique mapping strategy certainly underestimates the expression of young L1 elements, as reads that map to multiple locations are not considered. We included a sentence to make this point clearer at the beginning of the discussion section.

6. A key issue that remains unresolved is the functional of L1-silencing/activation in mESC. Does this change cell morphology, pluripotency, or differentiation potential? This is essential information.

In our revised version, we provide additional data about cell proliferation, cell death, cell cycle, pluripotency and DNA damage. This is shown in **Fig.S2D**; **Fig.S3**. Briefly, we observed that L1MdTf upregulation, but not downregulation, has a significant impact on cell proliferation/cell death and in the formation and size of mESC colonies. However, we did not observe any changes on cell cycle progression and DNA double strand breaks levels. Moreover, the expression of pluripotency and differentiation markers is only mildly affected.

7. Fig 3B: Is this n=2? Hence why there is no statistical analyses? Same for Fig 5A-B: here a p-value is presented but it would be good to highlight the statistically significant results somehow.

The former Fig.3B showed distributions based on data from one RNA-seq library. In the updated figure panel (now **Fig.3D**), the violin plots represent the distribution of average expression fold changes for individual L1MdTf repeats classified by subfamily (L1MdTf_I, _II and _III) for each clone considering the two RNA-seq libraries. We have also included annotations to indicate significant differences between the distributions. The statistical test to assess differences in expression of specific L1MdTfs was performed by DESeq2 using the RNA-seq duplicates and discussed in the "*Engineered effectors affect expression of a large number of L1MdTf elements*" section.

In addition, we have highlighted significant p-values in **Fig.5A**/**5B**, as well as in **Fig.S7A**/**S7B**, to make it clearer for the reader.

8. Are only L1MdTf elements plotted in Fig 3D and 3E? As the authors claim that the system is quite specific, it would be good to have a clear view of the repeats that are differentially expressed and are evolutionarily close to the L1MdTf family. The figure might also benefit of some further characterization on a family level (multi-mapping) rather than single elements (unique mapping or multi mapping with reads that map to a single location, like here).

In Fig.3D and 3E (now **Fig.3F-G**), the heat maps display the expression fold change and normalized expression of all DERs (including L1MdTfs but also other repeats - cf. Table S7 and S8), identified with the unique mapping strategy. As already mentioned in point 4, the exact numbers are included in the results

section and information for each repeat can be found in the supplemental tables (Table S9 and Table S10). Nevertheless, we included an additional column to highlight repeats of L1MdTf subfamilies.

We agree that family level analyses are also relevant, and for this reason, we show in Fig.S3 normalized read counts of different families of repeats and for LINE-1 subfamilies, some of which being evolutionarily young and close to the L1MdTf subfamily (L1MdGf_I and Gf_II; L1MdA_I to A_III). Of note, these data are based on the random mapping strategy that consisted of assigning multi-mapped reads to a single location randomly.

9. The authors provide some evidence for the existence of a bidirectional promoter on L1MdTf elements. In what elements was this observed? Does it result in chimeric transcripts? And if so, are these genes in line with the enrichment found?

We appreciate the reviewer's interest in the potential bidirectional promoter activity of L1MdTf elements.

The fact that signal is observed using metagene plots (average values) already indicates that the bidirectional transcription is not only present in 1 or 2 elements but rather a broader phenomenon across many L1MdTf elements. Furthermore, this activity is noticeably exacerbated upon DOX treatment in the activator clones. Yet, to address this concern, we have generated heat maps of the RNA-seq signal for individual L1MdTf elements, across the analyzed clones in -/+DOX conditions (see example below). These maps indicate that L1MdTf antisense transcription is not limited to a few elements. In addition, as illustrated with the examples of the *Iqub* gene shown in **Fig.S7E**, this antisense activity can give rise to chimeric transcripts, which become more abundant upon DOX induction. Given that these results do not provide mechanistic insights beyond what the metagene plots already show, we decided to not include these heat maps on the manuscript.

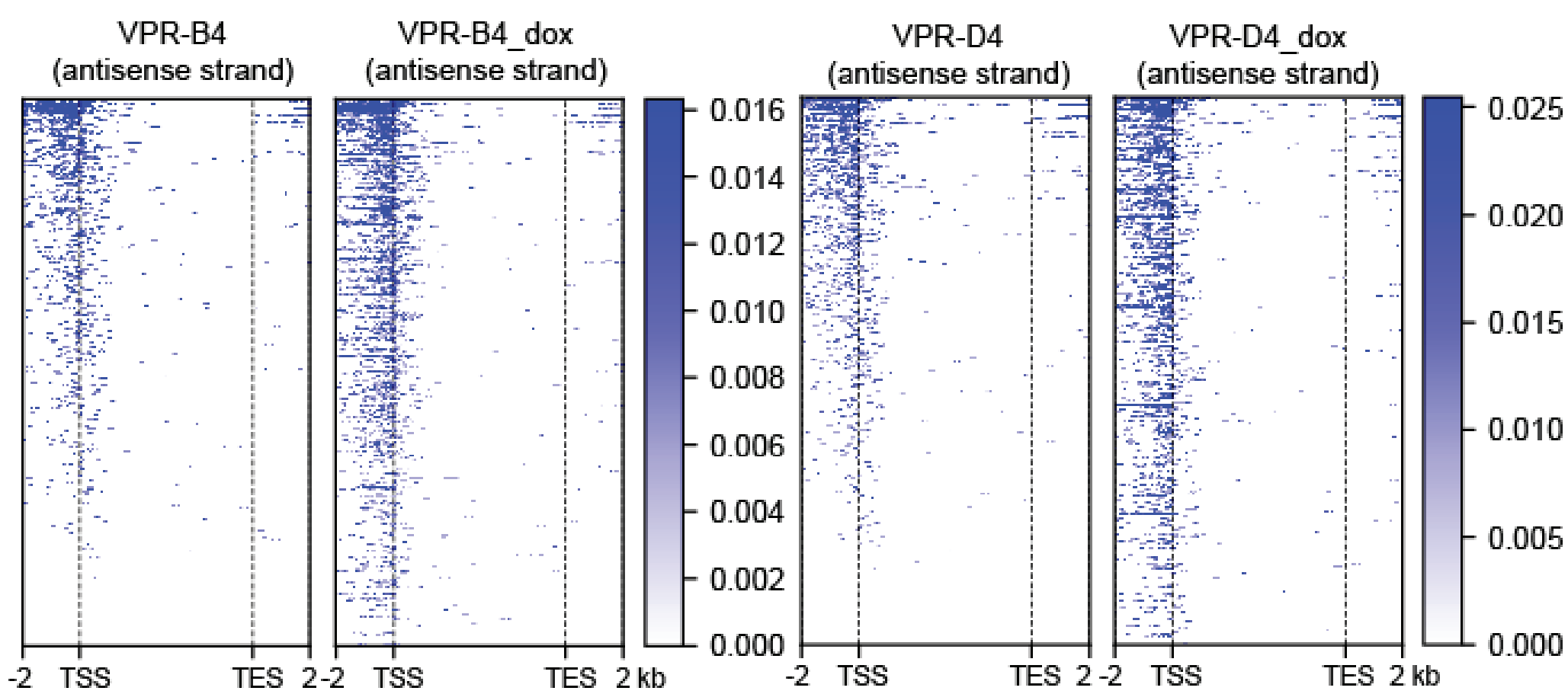


Panels showing heat maps of antisense RNA-seq signals across single L1MdTFs in clone VPR-B4 (-/+DOX; left) and VPR-D4 (-/+DOX; right).

Minor comments:

1. The introduction would benefit from a more detailed description of the L1-content in the mouse genome including the presence of polymorphic elements.

In the introduction, we refer to the Sookdeo et al. (2013) study and the L1base2 resource paper (Penzkofer 2021), which are key reference publications regarding the L1 content and annotations in the mouse genome. For the presence of polymorphic elements, we cite the Ferraj et al study (2023) in the discussion, which explores structural variants caused by TEs across different mouse strains.

2. There is no characterization of the edited mESC. Stainings? Markers from bulk RNA seq analysis? Morphology assessment?

As explained in our answer for point #6, we have now included a supplementary figure (**Fig.S2D**; **Fig.S3**) providing additional data about cell proliferation, cell death, cell cycle, DNA damage and expression analysis of marker genes (pluripotency and lineage-specific) upon DOX induction in our engineered ES cell lines.

Reviewer #2 (Significance (Required)):

The manuscript is timely and in line to some similar and very recent studies suggesting a link between evolutionary young L1s and neuronal-related genes in mice (e.g. Toda et al.). However, the manuscript is quite preliminary in the current form. The investigation of potential off-target effects of their system requires more in-depth investigations. In addition, the manuscript could be improved with further exploration into the functional consequences of L1-silencing/activation.

We thank the reviewer for recognizing the timeliness of our manuscript and its connection to recent work on young L1s and neuronal genes. We have significantly revised the manuscript to address the concerns regarding both off-target effects and the functional consequences of L1 modulation. These additions significantly strengthen the findings and set the manuscript beyond a preliminary stage by robustly validating our tools and providing clear functional evidence.

Regarding the off-targets concern, we have now incorporated ChIP-seq data for dCas9 in the activation clones, demonstrating that the vast majority of dCas9 binding sites target L1MdTf elements as intended. This confirms that the number of off-targets is minimal and the system is highly specific.

We have substantially expanded the functional consequences of L1MdTf modulation with several experiments that directly explore the functional role of L1MdTfs, including deletion of specific elements. Our results highlight the endogenous regulatory potential of L1MdTfs on the mouse ESC transcriptome.

**Reviewer #3 (Evidence, reproducibility and clarity (Required)):**

Perez-Rico et al use dCAS9 to activate and repress L1 expression levels in the subfamily level for younger mouse L1s to address their cisregulatory potential

They choose to transcriptionally perturb full-length L1s subfamily.
An important topic, and I liked the approach of using CRISPR to perturb an entire subfamily of transposable elements. However, this study has yet to add any new findings or conclusions. This study can be improved by further analysis or experimentation. The regulatory role of L1s in cis-regulatory

function has been previously demonstrated in mouse and human stem cell systems using multiple approaches, including CRISPR-mediated perturbations, CRISPR-based deletions, and 3D chromatin structure analysis. My main concern is the analysis is very superficial and lacks integration of other datatypes such as HiC to further support the role of L1s in enhancer activity and further validation on candidate loci.

One of the major limitations of CRISPR-based perturbations is that it can be difficult to conclude the cis-regulatory role of these targeted elements (L1) as synthetic tethering of VPR or KRAB domain can indirectly spread their effect in cis, which otherwise may not have a specific cis-regulatory role to regulates genes in cis.

Some of the major comments that needs to be addressed to strengthen the manuscript conclusions

1) One of the important controls could be targeting different or older L1s to show how it can affect gene expression. can authors perturb older families?

We understand the reviewer's concern about the potential spreading effects of KRAB or VPR effectors, which could impact nearby genes beyond direct *cis*-regulation. While we agree that testing older L1 families could provide additional insights about cis-regulation mediated by repeat elements, it will not clarify the specific functions of L1MdTfs. Older L1 subfamilies exhibit more sequence divergence, reducing gRNA design efficiency, therefore, targeting a comparable number of loci would require extensive re-optimization beyond the scope of this study. Importantly, L1MdTfs retain full-length ORFs and demonstrated activity potential, unlike transcriptionally inert older families. Thus, perturbing inert repeats would likely yield null results, while our results include examples of DEG correlating with L1MdTf activation. In addition, even if we would manage to perturb the expression of older L1s and observe changes in expression, any effects on nearby gene expression would still raise the same question: are these changes due to physical proximity and binding alone, or do they result from actual transcriptional perturbation?

2) Although meta-analysis of L1s show up/downregulation of L1MdTf, ChIPseq for dCAS9 is needed to demonstrate which L1s are directly targeted in the experiment and analyse the data separately for dCAS9 bound L1s vs rest of the L1s in the subfamily.

In the revised version of our manuscript, we now provide ChIP-seq data for dCas9 in the VPR control (B7) and activation clones (B4 & D4). A similar experiment could not be performed in the repression clones, because of the lack of suitable antibody for the LKF/AKF effector.

The data in **Fig.3B,C**/**Fig.S10A** show that dCas9–VPR targets L1MdTf elements with high specificity. ChIP-seq peaks largely corroborate the targets predicted by our initial *in silico* analysis (**Fig.3C**). Also, the number of off-target binding sites is minimal; we detected overlap with 14 L1 elements from other subfamilies and 3 genes (one shared between the two clones) (**Table S3**).

In our analyses (**Fig.3D-G**/**Fig.S5**; **Fig.5**/**Fig.S7A-C**), we actually handle L1MdTfs according to their classification as differentially expressed or targeted elements, and analyses for which all annotated L1MdTfs were considered are clearly labelled in the figures/figure legends.

3) L1s' transcriptional activation could be independent of their cis-regulatory activity. So, it is important to analyse the enhancer chromatin feature of TEs: Do they make 3D contact with the nearby genes?

To address this point, we analyzed to which extent DEGs and targeted L1MdTfs are located in the same TADs, using Hi-C data from ES cells (Bonev et al, 2017; 10.1016/j.cell.2017.09.043). We observed a significant association between the presence of DEGs and targeted L1MdTfs on TADs (**Fig.S8B**) and identified chromatin loops between DEGs and L1MdTfs (examples in **Fig.6D**). For the identified loops, we provide a supplementary table (**Table S24**), indicating which L1-loop anchors *cis*-regulatory elements annotated by ENCODE were observed.

These results were included in the manuscript as follows:

*"To account for 3D genome organization beyond linear distance, we examined co-localisation of DEGs and targeted L1MdTf within topologically associating domains (TADs) (Bonev et al, 2017). Notably, 51.4% of TADs containing VPR-upregulated genes also harbored targeted L1MdTfs, in contrast to 12.8% for downregulated genes. In the LKF clone, the opposite pattern was observed: TADs with downregulated genes were significantly enriched for L1MdTfs (Fig.S8B)."*

*"Notably, all chromatin loops detected between targeted L1MdTfs and DEGs involve genes associated with neuronal functions (Fig.6D; Table S24)."*

4) how many replicates of RNA seq is done?

We sequenced two biological replicates of each clone. This information is included in the methods section of the manuscript.

5) Are genes that respond to CRISPR activation in the same TAD? Do they loop with LINE1?

As explained in point #3, we have now included TAD annotations to deepen on the distance analyses and also searched for chromatin loops between DEGs and L1MdTfs.

6) Figures 4 A &B show clear perturbation of host genes upon CRISPRi. However, they need to show L1s outside the genes (not in the introns) as CRISPR dCAS9 perturbation of Intronic L1s is expected to affect the expression of genes.

In the revised version of this manuscript, we provide an experiment where we perform targeted deletion of two L1MdTf elements in the repressor clone LKF-A6. One example concerns the *Reg2* gene, located ~3 kb downstream an intergenic L1MdTf element and whose expression is affected upon repression (**Fig.S9G,H**). Furthermore, we have also included in **Fig.S9D,E** examples of two representative genes (*Cysltr2* and *Gm48691*) located nearby intergenic L1MdTfs and downregulated upon L1MdTf repression. For the sake of space, we could not include more examples.

7) One of the key experiment missing is to genetically delete candidate L1s that are intergenic that are predicted to have a cis-regulatory function in the RNAseq data.

As suggested by the reviewer, we have now performed targeted deletions of two L1MdTf elements in the repressor clone LKF-A6: one intergenic element located near the *Reg2* gene, and one intronic element within the *Clca3b* gene. As shown in **Fig.S9G,H**, deletion of either element recapitulates the effects observed upon DOX-induced downregulation of L1MdTf expression, namely, reduced expression of the nearby genes *Reg2* and *Clca3b*. These results support a *cis*-regulatory role for these two L1MdTf elements.

8) Authors should present the RNAseq data for genes 10 to 100 kb away from dCAS9-KRAB and dCAS9-VPR targeted L1s. It will be interesting to compare KRAB v VPR effect with distance.

We performed this analysis by looking at the log2FC of expression (+/-DOX) of common VPR-upregulated or LKF-downregulated genes, grouped based on their distance to targeted L1s, within windows between 0 and 200kb. The data presented below show that for both the repression or the activation systems, genes located from 0 to ~150 kb away from targeted L1s have a higher/lower FC than genes located further away from targeted L1s. In addition, some genes located further away (more than 200 kb) from targeted elements show FC value significantly different than 0, indicating long-range transcriptional effects of the perturbed L1s or trans-regulation. However, given that the number of LKF-downregulated genes is much lower than that of common VPR-upregulated, we cannot meaningfully compare KRAB vs VPR effects. We therefore chose not to include these data in the manuscript, and focus on the Hi-C data that overcomes limitations of linear distances.

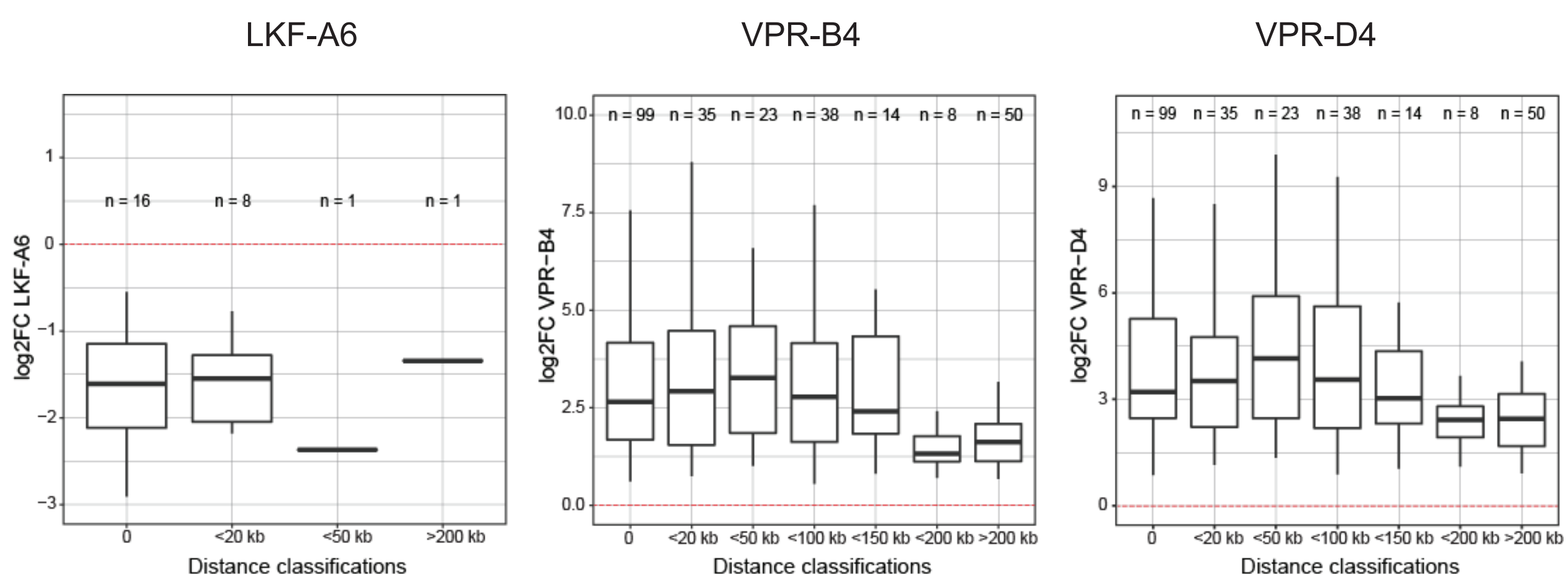


9) The suggested role of L1s in neuronal gene expression is not strongly supported by their experiments or anlaysis. The authors end the results section with "Most of these genes are not expressed or have low expression levels in ESCs compared to other upregulated genes that are not part of these processes and pathways (Fig.S9E)". This result suggests that dCAS9-VPR targeting to multiple (How many are targeted is missing) L1s leads to the activation of many genes that are not normally expressed in mESCs due to the spreading of transcriptional activators or mESCs getting spontaneously differentiated to the neuronal lineage. Authors need to address these points.

Regarding the number of targeted elements, the first paragraph of the results section reports the in silico prediction of ~1,000 full-length and ~1,000 shorter L1MdTf elements as potential targets for both effectors, LKF and dCas9-VPR. In this revised version, this estimate is now further supported by our dCas9

ChIP-seq data, which revealed a number of binding sites comparable with our predictions (**Fig.3C**; **Table S3**).

To check for possible differentiation of our ESC lines following L1 upregulation, we examined the expression of pluripotency and early differentiation marker genes in the activator clones following DOX induction by qPCR. The results revealed only modest changes in gene expression, consistent with our RNA-seq data. In addition, ES cell morphology and colony formation showed no signs of differentiation. Overall, this suggests that pluripotency is maintained and there is no evidence of spontaneous differentiation.

Finally, regarding potential gene misregulation as a consequence of spreading of VPR effectors, this is something that we cannot exclude for all loci. However, as indicated in our previous answers, our ChIP-seq data indicate that proximal binding of the dCas9 -VPR effector does not necessarily lead to misregulation of nearby genes (representative examples in **Fig.S10A**).

Minor comments
Fig 2E) which housekeeping gene

Gene expression was normalised to two housekeeping genes (*Bactin*, *Rrm2*) as indicated in the methods section.

Figures 4 A &B, show if these L1s make looping interactions or are in the same TAD as target genes.

*Pkhd1* is a long gene (~560kb) comprising 67 exons. The TAD annotated at this locus begins just upstream of exon 32 encompasses the two intronic L1MdTf elements highlighted in Fig.4A and extended beyond the end of the gene. The annotated chromatin loops do not contact these L1MdTf elements; but one loop instead contacts a nearby young L1MdA element. For *Iqub*, there are no annotated TAD boundaries or loops interacting over the gene.

As this information does not add any further information besides what is described in the manuscript regarding TAD/chromatin loop annotations, we decided not to show this in Fig.4A.

**Referee Cross-commenting**

I agree with the other two reviewers points.

Reviewer #3 (Significance (Required)):

This study can be improved by further analysis or experimentation. In its current version, the conceptual advance offered by the current results and approach of the manuscript seem limited.

We appreciate the reviewer's constructive feedback that has significantly strengthened the conceptual advance of our study with the new data and analyses incorporated. Our major revisions have comprehensively addressed all concerns and mainly include:

- Direct functional evidence that L1MdTf elements are capable of influencing the expression of neighboring genes in cis in ESCs through targeted deletion experiments. This moves beyond mere correlation to establish direct regulation.
- The inclusion of Hi-C analysis provides critical insights into the 3D chromatin context of L1MdTf elements. This data reveals that L1MdTfs often reside in the same domains as genes impacted by the effectors and some of them form loops with promoter-proximal regions. These findings could explain how an L1 element, even when apparently distal, could physically regulate gene expression.
- We robustly validated our CRISPR/dCas9 system through dCas9 ChIP-seq, confirming its high on-target specificity at L1MdTf promoters, with minimal off-target binding, establishing a reliable tool for future studies on repetitive element regulation.

**1st Editorial Decision** **24th Apr 2026**

Dear Anne-Valerie,

Thank you for the submission of your revised manuscript. We have now received the enclosed report from referee 1, who was asked to assess it. Referee 1 still has some more comments that I would like you to address and incorporate before we can proceed with the official acceptance of your manuscript.

A few editorial requests will also need to be addressed:

- Please add a "Data Availability Statement" (DAS) to the end of the Methods. It should list data that were generated during this study and deposited in public databases. If no such data were generated, please mention this in the DAS.

- The conflict of interest subheading needs to be corrected to "Disclosure and Competing Interests Statement"

- Per journal policy, the statement DATA NOT SHOWN (p4, p9, p12) must be removed. Please either show the data or re-write.

- Please co-submit with your final ms a completed author checklist, which you can download from our author guidelines <https://link.springer.com/journal/44319/submission-guidelines>. The completed author checklist will also be part of the transparent peer-review file.

- Please upload individual production quality Figure files with your final ms. There are 11 suppl. figures, these can either be published in an Appendix file, or some of these can be published as EV figures. In either case, the nomenclature needs to be corrected in all places (source file name, title in the system, legend in the ms, callout in the ms). "Supplemental" should not be used. You can find more information in our guide to authors online.

- There are 3 Excel files uploaded with 26 suppl. tables. The nomenclature of all of these items need to be updated to Datasets and each Dataset needs to have its legend in the same file (dataset legends should be removed from the ms). There are two options:
1) The 3 Excel files can be renamed to Dataset EV1 - Dataset EV3 and each tab should be updated to Dataset EV1a, Dataset EV1b,... Dataset EV2a, Dataset EV2b, etc... Dataset EV3a, Dataset EV3b, etc. Each legend then needs to be provided in the same tab, labeled in the same way (not as Table S1, etc.)
2) Each tab can be upldd as a separate dataset so that we have 26 datasets Dataset EV1-Dataset EV26 and each legend can then be provided as a separate sheet/tab in that Excel file.

- There are also 2 text files uploaded as Data S1, Data S2; since this nomenclature is incorrect, please update these to Dataset EV#; their legends also need to be removed from the ms and provided in their respective txt files.

- The Materials and Methods section should include a separate Reagents and Tools Table file (listing key reagents, experimental models, software and relevant equipment and including their sources and relevant identifiers) and a Methods and Protocols section in which methods should be described using a step-by-step protocol format with bullet points. More information and a downloadable Table template is available in our guide to authors online: https://link.springer.com/journal/44319/submission-guidelines

* Figure Legends - Comments *

- Please note that the legend for figure 6D is missing in the manuscript. This needs to be rectified.
- Please note that the exact p values are not provided in the legends of figures 2C,E; 3D; 4D. Please provide exact values as reasonable.
- Please indicate the statistical test used for data analysis in the legends of figures 4B.
- Please note that the box plots need to be defined in terms of minima, maxima, centre, bounds of box and whiskers, and percentile in the legends of figures 3D; 5A,B.
- Please note that information related to n is missing in the legends of 3D; 4B; 5A,B.
- Please note that the measure of center for the error bars needs to be defined in the legends of figures 2C,E.
- Please note that the scale bar is missing for figures 2A,I.

I would like to suggest some minor changes to the second half of the abstract that needs to be written in present tense. Do you agree with this :

Here, we examine this systematically using engineered mouse embryonic stem cells in which expression of one active L1 subfamily is perturbed. We find that FL-L1 activation leads to the misregulation of ~1,024 genes, whereas FL-L1 repression affects ~81 genes. In most cases (68%), misexpressed genes contain an intronic FL-L1 or lie near a FL-L1 (<260 kb). Gene

ontology analysis shows that upon L1 activation, upregulated genes are enriched for neuronal function-related terms, suggesting that some L1 elements may have evolved to control neuronal gene networks. These results illustrate the cis-regulatory potential of FL-L1 elements and suggest a broader role for L1s than anticipated.

EMBO press papers are accompanied online by A) a short (1-2 sentences) summary of the findings and their significance, B) 2-3 bullet points highlighting key results and C) a synopsis image that is exactly 550 pixels wide and 300 pixels high. The synopsis image should provide a sketch of the major findings, like a graphical abstract. Please note that text needs to be readable at the final size. Please send us this information along with the final manuscript.

I look forward to seeing a final version of your manuscript as soon as possible.

Best regards,
Esther

Esther Schnapp, PhD
Senior Editor
EMBO reports

Referee #1:

The authors have addressed most of my initial concerns. However, one major issue persists, along with several minor points that need clarification.

Major point:

1. However, defining the true set of L1 loci effectively bound by Cas9 remains problematic, as it is unclear how multimapping reads influence the reported output. The main text states that "150 bp paired-end sequencing allowed mapping the signal to individual L1 repeats", but my understanding of the methods section suggests that multimapping reads were retained, although what this means in practice (redundant assignment, fractional weighting, or random allocation) is left ambiguous. A direct consequence is that the least-divergent copies, those most similar to the consensus sequence used to design the gRNAs, will have reads assigned to them in a largely arbitrary manner. The near-perfect overlap between loci predicted to be targeted by the gRNA and those reported as effectively targeted is therefore likely an artefact of this assignment strategy rather than a biological signal. If so, conclusions drawn from the apparent absence of transcriptional activation at "targeted" L1 loci (p. 9, 2nd paragraph) become difficult to sustain, and any comparison or fraction involving loci that are targeted but not activated would be substantially biased. I appreciate that this is an inherent limitation of ChIP-seq combined with short-read sequencing when applied to transposable elements, and particularly to the long, multimeric 5' UTR of murine L1 elements. At a minimum, however, the authors should explicitly acknowledge this limitation and temper their conclusions accordingly. Ideally, they should repeat the analysis retaining only uniquely mapping reads (unimappers), which would provide a more conservative but reliable set of effectively targeted loci, even if this comes at the cost of reduced coverage. With this approach, false negatives (effectively targeted but not detected due to conservative ChIP-seq peak calling) could be estimated by looking at DE genes in the vicinity of an L1 predicted to be targeted by CRISPRa/i. In addition, more explicit terminology is needed throughout the text, figures, and figure legends to distinguish in silico prediction from actual binding of the effector. The authors could use "predicted binding sites" or "theoretically targeted loci" (derived from in silico analysis), and "observed binding sites" or "effectively targeted loci" (derived from ChIP-seq data), rather than using the ambiguous term "targeted loci".

Other points:

2. Fig. 4B (and other similar volcano plots): the color legend corresponds to the absolute value of fold change, not fold change itself.
3. Fig. S8B: if this figure is kept in this form or another, the x-axis is unclear, as it is not evident what "present/absent" refers to, and how this differs from L1 element presence or absence (color). The schematic should be more explicit.
4. Fig. S8: if this figure is kept in this form or another, statistical testing should use a chi-squared or Fisher's exact test with "no change" as the reference category, followed by correction for multiple comparisons.

Further comments from Referee 1:

I went through their response to all referee comments. It is a bit frustrating that they haven't used a tagged version of their repressor system, which would have allowed them to perform ChIP-seq for both effectors. But changing this at this stage would basically mean repeating half of the paper. I don't think they can do much better. My final concerns should be manageable without any new experiments, only data analysis and a bit of text editing.

**Please find below our answers to the remaining concerns from Referee #1 and to the editorial requests.**

Referee #1:

The authors have addressed most of my initial concerns. However, one major issue persists, along with several minor points that need clarification.

Major point:

1. However, defining the true set of L1 loci effectively bound by Cas9 remains problematic, as it is unclear how multimapping reads influence the reported output. The main text states that "150 bp paired-end sequencing allowed mapping the signal to individual L1 repeats", but my understanding of the methods section suggests that multimapping reads were retained, although what this means in practice (redundant assignment, fractional weighting, or random allocation) is left ambiguous. A direct consequence is that the least-divergent copies, those most similar to the consensus sequence used to design the gRNAs, will have reads assigned to them in a largely arbitrary manner. The near-perfect overlap between loci predicted to be targeted by the gRNA and those reported as effectively targeted is therefore likely an artefact of this assignment strategy rather than a biological signal. If so, conclusions drawn from the apparent absence of transcriptional activation at "targeted" L1 loci (p. 9, 2nd paragraph) become difficult to sustain, and any comparison or fraction involving loci that are targeted but not activated would be substantially biased. I appreciate that this is an inherent limitation of ChIP-seq combined with short-read sequencing when applied to transposable elements, and particularly to the long, multimeric 5' UTR of murine L1 elements. At a minimum, however, the authors should explicitly acknowledge this limitation and temper their conclusions accordingly. Ideally, they should repeat the analysis retaining only uniquely mapping reads (unimappers), which would provide a more conservative but reliable set of effectively targeted loci, even if this comes at the cost of reduced coverage. With this approach, false negatives (effectively targeted but not detected due to conservative ChIP-seq peak calling) could be estimated by looking at DE genes in the vicinity of an L1 predicted to be targeted by CRISPRa/i. In addition, more explicit terminology is needed throughout the text, figures, and figure legends to distinguish in silico prediction from actual binding of the effector. The authors could use "predicted binding sites" or "theoretically targeted loci" (derived from in silico analysis), and "observed binding sites" or "effectively targeted loci" (derived from ChIP-seq data), rather than using the ambiguous term "targeted loci".

We thank the reviewer for making us aware of the potential misunderstanding regarding the terminology used to refer to targeted sites and have modified the manuscript and figures to clearly differentiate between predicted and ChIP-seq sites. In addition, we have included an explanation of the parameters used to map the ChIP-seq data in order to explicitly describe how multimapping reads were assigned. We fully agree with the reviewer that the youngest repetitive elements are the most challenging to quantify accurately by ChIP-seq at their promoters, as reads originating from different genomic locations can be indistinguishable. Thus with our previous methodology, these reads could map to multiple locations equally well, but only one of the best alignments is reported, which could indeed lead to increased signal for a repeat of the same subfamily rather than reflecting the true element from which the read originated. With this in mind, we now state this limitation in the Discussion. As the initial aim of the ChIP-seq experiments was to discard off-target binding events at genes or

other repeat families, which could explain the large transcriptional changes that we observed upon L1MdTf activation, we kept the results of the analyses including multimapping reads as a control to increase the sensitivity of peak detection while searching for potential off-targets (shown in Appendix Fig. S2A). In fact, these comprehensive sets of mapped reads identified few peaks (13 and 18 for VPR-B4 and VPR-D4, respectively) outside L1MdTf elements, indicating the specificity of the CRISPRa system. However, we now include the results of the conservative analyses using only uniquely mapped reads in the main figures (Fig. 3B,C). As expected, the number of peaks decreased relative to the previous analyses, but ~800 peaks could be detected for the two clones and ~99% of the common peaks overlap L1MdTfs with predicted binding sites. As a result, figures showing ChIP-seq tracks were updated to show uniquely mapped peaks only, or, in some cases, both uniquely and multimapped peaks. Some of the representative examples shown in Appendix Figure S6A (former Figure S10), which display uniquely mapped peaks, were revised. Finally, as suggested by the reviewer, for most of the analyses, we work with the sets of *in silico* predicted L1MdTf targets to minimize false negatives and we have improved the terminology used throughout the text.

Other points:
2. Fig. 4B (and other similar volcano plots): the color legend corresponds to the absolute value of fold change, not fold change itself.
We have modified the legend on the figure panel to make sure it represents the absolute value of the fold change.

3. Fig. S8B: if this figure is kept in this form or another, the x-axis is unclear, as it is not evident what "present/absent" refers to, and how this differs from L1 element presence or absence (color). The schematic should be more explicit.
We have changed the labels of the x-axis and y-axis to make it clearer.

4. Fig. S8: if this figure is kept in this form or another, statistical testing should use a chi-squared or Fisher's exact test with "no change" as the reference category, followed by correction for multiple comparisons.
We performed Fisher’s exact tests as suggested, included the adjusted p-values in the figure and modified the legend and methods accordingly.

Further comments from Referee 1:

I went through their response to all referee comments. It is a bit frustrating that they haven't used a tagged version of their repressor system, which would have allowed them to perform ChIP-seq for both effectors. But changing this at this stage would basically mean repeating half of the paper. I don't think they can do much better. My final concerns should be manageable without any new experiments, only data analysis and a bit of text editing.

As the reviewer points out, the lack of a tagged repressor prevented us from directly defining observed binding sites in the repression condition. We agree that this is a limitation, but we believe that the limited number of transcriptional changes observed upon repression makes extensive off-target effects less likely.

**Editorial requests:**

A few editorial requests will also need to be addressed:

- Please add a "Data Availability Statement" (DAS) to the end of the Methods. It should list data that were generated during this study and deposited in public databases. If no such data were generated, please mention this in the DAS.
Done.

- The conflict of interest subheading needs to be corrected to "Disclosure and Competing Interests Statement"
Done.

- Per journal policy, the statement DATA NOT SHOWN (p4, p9, p12) must be removed. Please either show the data or re-write.
Done.

- Please co-submit with your final ms a completed author checklist, which you can download from our author guidelines <https://link.springer.com/journal/44319/submission-guidelines>;. The completed author checklist will also be part of the transparent peer-review file.

- Please upload individual production quality Figure files with your final ms. There are 11 suppl. figures, these can either be published in an Appendix file, or some of these can be published as EV figures. In either case, the nomenclature needs to be corrected in all places (source file name, title in the system, legend in the ms, callout in the ms). "Supplemental" should not be used. You can find more information in our guide to authors online.

- There are 3 Excel files uploaded with 26 suppl. tables. The nomenclature of all of these items need to be updated to Datasets and each Dataset needs to have its legend in the same file (dataset legends should be removed from the ms). There are two options:
1) The 3 Excel files can be renamed to Dataset EV1 - Dataset EV3 and each tab should be updated to Dataset EV1a, Dataset EV1b,... Dataset EV2a, Dataset EV2b, etc... Dataset EV3a, Dataset EV3b, etc. Each legend then needs to be provided in the same tab, labeled in the same way (not as Table S1, etc.)
2) Each tab can be upldd as a separate dataset so that we have 26 datasets Dataset EV1-Dataset EV26 and each legend can then be provided as a separate sheet/tab in that Excel file.

- There are also 2 text files uploaded as Data S1, Data S2; since this nomenclature is incorrect, please update these to Dataset EV#; their legends also need to be removed from the ms and provided in their respective txt files.

- The Materials and Methods section should include a separate Reagents and Tools Table file (listing key reagents, experimental models, software and relevant equipment and including their sources and relevant identifiers) and a Methods and Protocols section in which methods should be described using a step-by-step protocol format with bullet points. More information and a downloadable Table template is available in our guide to authors online: https://link.springer.com/journal/44319/submission-guidelines

* Figure Legends - Comments *

- Please note that the legend for figure 6D is missing in the manuscript. This needs to be rectified.

This is rectified.

- Please note that the exact p values are not provided in the legends of figures 2C,E; 3D; 4D. Please provide exact values as reasonable.

Exact p-values are now provided, either in the figure itself or in the figure legend.
For figure 4D, the reported p-value corresponds to the standard lower limit of numerical precision for this type of test in R (the machine epsilon for 64-bit doubles). Due to the arithmetic limitation, the software cannot calculate a more granular exact p value. For the other figures, exact values are now included.

- Please indicate the statistical test used for data analysis in the legends of figures 4B.
- Please note that the box plots need to be defined in terms of minima, maxima, centre, bounds of box and whiskers, and percentile in the legends of figures 3D; 5A,B.
- Please note that information related to n is missing in the legends of 3D; 4B; 5A,B.
- Please note that the measure of center for the error bars needs to be defined in the legends of figures 2C,E.
- Please note that the scale bar is missing for figures 2A,I.

All of the above points have been addressed.

I would like to suggest some minor changes to the second half of the abstract that needs to be written in present tense. Do you agree with this :

Here, we examine this systematically using engineered mouse embryonic stem cells in which expression of one active L1 subfamily is perturbed. We find that FL-L1 activation leads to the misregulation of ~1,024 genes, whereas FL-L1 repression affects ~81 genes. In most cases (68%), misexpressed genes contain an intronic FL-L1 or lie near a FL-L1 (<260 kb). Gene ontology analysis shows that upon L1 activation, upregulated genes are enriched for neuronal function-related terms, suggesting that some L1 elements may have evolved to control neuronal gene networks. These results illustrate the cis-regulatory potential of FL-L1 elements and suggest a broader role for L1s than anticipated.

We agree with the changes and have modified the text accordingly.

EMBO press papers are accompanied online by A) a short (1-2 sentences) summary of the findings and their significance, B) 2-3 bullet points highlighting key results and C) a synopsis image that is exactly 550 pixels wide and 300 pixels high. The synopsis image should provide a sketch of the major findings, like a graphical abstract. Please note that text needs to be readable at the final size. Please send us this information along with the final manuscript.

A) Sentence:

Genome-wide transcriptional repression and activation of full length LINE-1 retrotransposons in mouse ES cells unmask their cis-regulatory potential on neighboring genes, notably within neuronal gene networks.

B) Bullet points:

- Hundreds of protein-coding genes are misregulated following LINE-1 transcriptional perturbation, mainly L1MdTf activation.
- L1MdTf repression and activation predominantly impact genes within the same regulatory neighborhood.
- Neuronal genes are more vulnerable to L1MdTf cis-regulatory effects.

C) Synopsis provided in PNG format with correct pixel dimensions.

Dr. Anne-Valerie Gendrel
Gulbenkian Institute for Molecular Medicine
Chromatin & Epigenetics
Lisbon
Portugal

Dear Anne-Valerie,

I am very pleased to accept your manuscript for publication in the next available issue of EMBO reports. Thank you for your contribution to our journal.

Your manuscript will be processed for publication by EMBO Press. It will be copy edited and you will receive page proofs prior to publication. Please note that you will be contacted by Springer Nature Author Services to complete licensing and payment information.

You may qualify for financial assistance for your publication charges - either via a Springer Nature fully open access agreement or an EMBO initiative. Check your eligibility: https://link.springer.com/journal/44319/how-to-publish-with-us

Should you be planning a Press Release on your article, please notify the production team via this form in order to coordinate publication and release dates: https://forms.cloud.microsoft/e/eH4A8JVP6g

If you have any questions, please do not hesitate to contact the Editorial Office. Thank you for your contribution to EMBO Reports.

Kind regards,
Esther

Esther Schnapp, PhD
Senior Editor
EMBO reports

------------------------------------------------

>>> Please note that it is EMBO Reports policy for the transcript of the editorial process (containing referee reports and your response letter) to be published as an online supplement to each paper. If you do NOT want this, you will need to inform the Editorial Office via email immediately. More information is available here: https://link.springer.com/partners/embo-press/editorial-policies#Peer%20review

## EMBO Press Author Checklist

| | |
|---|---|
| Corresponding Author Name: Anne-Valerie Gendrel | |
| Journal Submitted to: EMBO Reports | |
| Manuscript Number: EMBOR-2026-64014V1-T | |

**USEFUL LINKS FOR COMPLETING THIS FORM**
The EMBO Journal - Author Guidelines
EMBO Reports - Author Guidelines
Molecular Systems Biology - Author Guidelines
EMBO Molecular Medicine - Author Guidelines

**Reporting Checklist for Life Science Articles (updated January**
This checklist is adapted from Materials Design Analysis Reporting (MDAR) Checklist for Authors. MDAR establishes a minimum set of requirements in transparent reporting in the life sciences (see Statement of Task: 10.31222/osf.io/9sm4x). Please follow the journal's guidelines in preparing your manuscript.
**Please note that a copy of this checklist will be published alongside your article.**

**Abridged guidelines for figures**
**1. Data**
The data shown in figures should satisfy the following conditions:
- the data were obtained and processed according to the field's best practice and are presented to reflect the results of the experiments in an accurate and unbiased manner.
- ideally, figure panels should include only measurements that are directly comparable to each other and obtained with the same assay.
- plots include clearly labeled error bars for independent experiments and sample sizes. Unless justified, error bars should not be shown for technical
- if n<5, the individual data points from each experiment should be plotted. Any statistical test employed should be justified.
- Source Data should be included to report the data underlying figures according to the guidelines set out in the authorship guidelines on Data

**2. Captions**
Each figure caption should contain the following information, for each panel where they are relevant:
- a specification of the experimental system investigated (eg cell line, species name).
- the assay(s) and method(s) used to carry out the reported observations and measurements.
- an explicit mention of the biological and chemical entity(ies) that are being measured.
- an explicit mention of the biological and chemical entity(ies) that are altered/varied/perturbed in a controlled manner.
- the exact sample size (n) for each experimental group/condition, given as a number, not a range;
- a description of the sample collection allowing the reader to understand whether the samples represent technical or biological replicates (including how many animals, litters, cultures, etc.).
- a statement of how many times the experiment shown was independently replicated in the laboratory.
- definitions of statistical methods and measures:

- common tests, such as t-test (please specify whether paired vs. unpaired), simple χ2 tests, Wilcoxon and Mann-Whitney tests, can be unambiguously identified by name only, but more complex techniques should be described in the methods section;

- are tests one-sided or two-sided?
- are there adjustments for multiple comparisons?
- exact statistical test results, e.g., P values = x but not P values < x;
- definition of 'center values' as median or average;
- definition of error bars as s.d. or s.e.m.

**Please complete ALL of the questions below.**
**Select "Not Applicable" only when the requested information is not relevant for your study.**

**Materials**

| Newly Created Materials | Information included in the manuscript? | In which section is the information available? (Reagents and Tools Table, Materials and Methods, Figures, Data Availability Section) |
|---|---|---|
| New materials and reagents need to be available; do any restrictions apply? | Yes | Reagents and Tools table, Materials and Methods |

| Antibodies | Information included in the manuscript? | In which section is the information available? (Reagents and Tools Table, Materials and Methods, Figures, Data Availability Section) |
|---|---|---|
| For **antibodies** provide the following information:<br>- Commercial antibodies: RRID (if possible) or supplier name, catalogue number and or/clone number<br>- Non-commercial: RRID or citation | Yes | Reagents and Tools table, Materials and Methods |

| DNA and RNA sequences | Information included in the manuscript? | In which section is the information available? (Reagents and Tools Table, Materials and Methods, Figures, Data Availability Section) |
|---|---|---|
| **Short novel DNA or RNA including primers, probes**: provide the sequences. | Yes | Dataset EV10 and EV11 |

| Cell materials | Information included in the manuscript? | In which section is the information available? (Reagents and Tools Table, Materials and Methods, Figures, Data Availability Section) |
|---|---|---|
| **Cell lines:** Provide species information, strain. Provide accession number in repository **OR** supplier name, catalog number, clone number, and/**OR** RRID. | Yes | Reagents and Tools table, Materials and Methods |
| **Primary cultures:** Provide species, strain, sex of origin, genetic modification status. | Not Applicable | |
| Report if the cell lines were recently **authenticated** (e.g., by STR profiling) and tested for mycoplasma contamination. | Not Applicable | |

| Experimental animals | Information included in the manuscript? | In which section is the information available? (Reagents and Tools Table, Materials and Methods, Figures, Data Availability Section) |
|---|---|---|
| **Laboratory animals or Model organisms:** Provide species, strain, sex, age, genetic modification status. Provide accession number in repository **OR** supplier name, catalog number, clone number, **OR** RRID. | Not Applicable | |
| **Animal observed in or captured from the field:** Provide species, sex, and age where possible. | Not Applicable | |
| Please detail **housing and husbandry conditions**. | Not Applicable | |

| Plants and microbes | Information included in the manuscript? | In which section is the information available? (Reagents and Tools Table, Materials and Methods, Figures, Data Availability Section) |
|---|---|---|
| **Plants:** provide species and strain, ecotype and cultivar where relevant, unique accession number if available, and source (including location for collected wild specimens). | Not Applicable | |
| **Microbes:** provide species and strain, unique accession number if available, and source. | Not Applicable | |

| Human research participants | Information included in the manuscript? | In which section is the information available? (Reagents and Tools Table, Materials and Methods, Figures, Data Availability Section) |
|---|---|---|
| If collected and within the bounds of privacy constraints report on age, sex and gender or ethnicity for all study participants. | Not Applicable | |

| Core facilities | Information included in the manuscript? | In which section is the information available? (Reagents and Tools Table, Materials and Methods, Figures, Data Availability Section) |
|---|---|---|
| If your work benefited from core facilities, was their service mentioned in the acknowledgments section? | Yes | Acknowledgments section |

**Design**

| Study protocol | Information included in the manuscript? | In which section is the information available? (Reagents and Tools Table, Materials and Methods, Figures, Data Availability Section) |
|---|---|---|
| If study protocol has been **pre-registered, provide DOI in the manuscript**. For clinical trials, provide the trial registration number **OR** cite DOI. | Not Applicable | |
| Report the **clinical trial registration number** (at ClinicalTrials.gov or equivalent), where applicable. | Not Applicable | |

| Laboratory protocol | Information included in the manuscript? | In which section is the information available? (Reagents and Tools Table, Materials and Methods, Figures, Data Availability Section) |
|---|---|---|
| Provide DOI OR other citation details if **external detailed step-by-step protocols** are available. | Not Applicable | |

| Experimental study design and statistics | Information included in the manuscript? | In which section is the information available? (Reagents and Tools Table, Materials and Methods, Figures, Data Availability Section) |
|---|---|---|
| Include a statement about **sample size** estimate even if no statistical methods were used. | Yes | Materials and Methods, Figures |
| Were any steps taken to minimize the effects of subjective bias when allocating animals/samples to treatment (e.g. **randomization procedure**)? If yes, have they been described? | Not Applicable | |
| Include a statement about **blinding** even if no blinding was done. | Not Applicable | No blinding was performed. |
| Describe **inclusion/exclusion criteria** if samples or animals were excluded from the analysis. Were the criteria pre-established?<br><br>If sample or data points were omitted from analysis, report if this was due to attrition or intentional exclusion and provide justification. | Not Applicable | |
| For every figure, are **statistical tests** justified as appropriate? Do the data meet the assumptions of the tests (e.g., normal distribution)? Describe any methods used to assess it. Is there an estimate of variation within each group of data? Is the variance similar between the groups that are being statistically compared? | Yes | Materials and Methods section, Figure legends |

| Sample definition and in-laboratory replication | Information included in the manuscript? | In which section is the information available? (Reagents and Tools Table, Materials and Methods, Figures, Data Availability Section) |
|---|---|---|
| In the figure legends: state number of times the experiment was **replicated** in laboratory. | Yes | Figure legends |
| In the figure legends: define whether data describe **technical or biological replicates**. | Yes | Figure legends |

## Ethics

| Ethics | Information included in the manuscript? | In which section is the information available? (Reagents and Tools Table, Materials and Methods, Figures, Data Availability Section) |
|---|---|---|
| Studies involving **human participants**: State details of **authority granting ethics approval** (IRB or equivalent committee(s), provide reference number for approval. | Not Applicable | |
| Studies involving **human participants**: Include a statement confirming that **informed consent** was obtained from all subjects and that the experiments conformed to the principles set out in the WMA Declaration of Helsinki and the Department of Health and Human Services Belmont Report. | Not Applicable | |
| Studies involving **human participants:** For publication of **patient photos**, include a statement confirming that consent to publish was obtained. | Not Applicable | |
| Studies involving experimental **animals**: State details of **authority granting ethics approva**l (IRB or equivalent committee(s), provide reference number for approval. Include a statement of compliance with ethical regulations. | Not Applicable | |
| Studies involving **specimen and field samples:** State if relevant **permits** obtained, provide details of authority approving study; if none were required, explain why. | Not Applicable | |

| Dual Use Research of Concern (DURC) | Information included in the manuscript? | In which section is the information available? (Reagents and Tools Table, Materials and Methods, Figures, Data Availability Section) |
|---|---|---|
| Could your study fall under dual use research restrictions? Please check biosecurity documents and list of **select agents and toxins** (CDC): https://www.selectagents.gov/sat/list.htm | Not Applicable | |
| If you used a select agent, is the security level of the lab appropriate and reported in the manuscript? | Not Applicable | |
| If a study is subject to dual use research of concern regulations, is the name of the **authority granting approval and reference number** for the regulatory approval provided in the manuscript? | Not Applicable | |

## Reporting

The MDAR framework recommends adoption of discipline-specific guidelines, established and endorsed through community initiatives. Journals have their own policy about requiring specific guidelines and recommendations to complement MDAR.

| Adherence to community standards | Information included in the manuscript? | In which section is the information available? (Reagents and Tools Table, Materials and Methods, Figures, Data Availability Section) |
|---|---|---|
| State if relevant guidelines or checklists (e.g., **ICMJE, MIBBI, ARRIVE, PRISMA**) have been followed or provided. | Not Applicable | |
| For **tumor marker prognostic studies,** we recommend that you follow the **REMARK** reporting guidelines (see link list at top right). See author guidelines, under 'Reporting Guidelines'. Please confirm you have followed these guidelines. | Not Applicable | |
| For **phase II and III randomized controlled trials**, please refer to the **CONSORT** flow diagram (see link list at top right) and submit the CONSORT checklist (see link list at top right) with your submission. See author guidelines, under 'Reporting Guidelines'. Please confirm you have submitted this list. | Not Applicable | |

## Data Availability

| Data availability | Information included in the manuscript? | In which section is the information available? (Reagents and Tools Table, Materials and Methods, Figures, Data Availability Section) |
|---|---|---|
| Have **primary datasets** been deposited according to the journal's guidelines (see 'Data Deposition' section) and the respective accession numbers provided in the Data Availability Section? | Yes | Data Availability Statement |
| Were **human clinical and genomic datasets** deposited in a public access-controlled repository in accordance to ethical obligations to the patients and to the applicable consent agreement? | Not Applicable | |
| Are **computational models** that are central and integral to a study available without restrictions in a machine-readable form? Were the relevant accession numbers or links provided? | Not Applicable | |
| If publicly available data were reused, provide the respective **data citations in the reference list**. | Not Applicable | |
