# Supplementary material for: Transcriptional perturbation of LINE-1 elements reveals their cis-regulatory potential
Source: EMBO Rep. 2026 Jul 8;27(16):4824–54. doi: 10.1038/s44319-026-00854-w (PMC13503937; doi:10.1038/s44319-026-00854-w)
Supplement: Supplementary file 5 [file 44319_2026_854_MOESM17_ESM.zip › Figure 2/2B/AKF-B5_LKF-A5-A6_Novocyte_report.pdf]

Report of 180606_1731 -G10-B5_A5_A6

File Name: 180606_1731 -G10-B5_A5_A6_Novocyte

Run Time: 11/03/2019 19:41

Cytometer: NovoCyte 451140710935

Software: NovoExpress 2.2.0

Specimen1

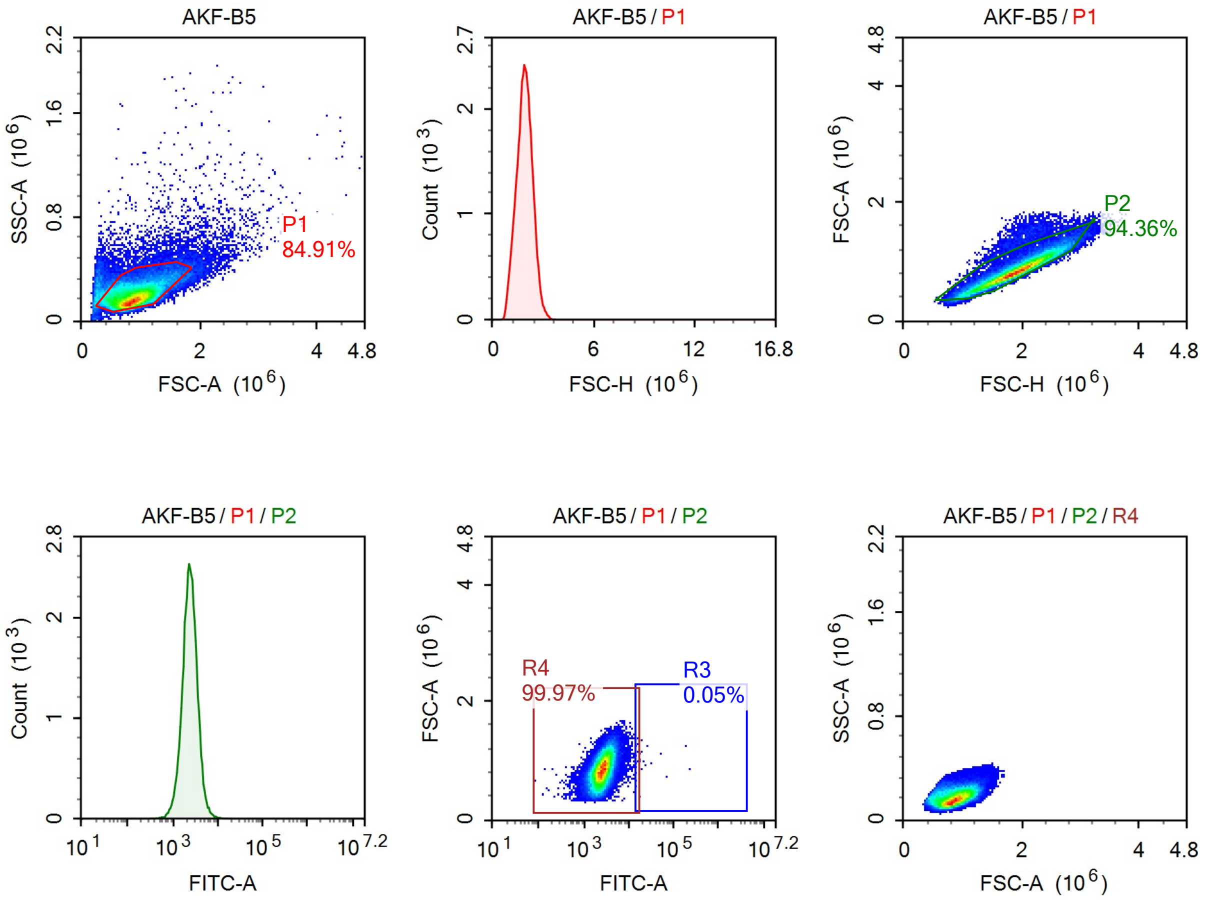

Sample Statistics of AKF-B5

Gate	Count	% Parent	X	Y	Median X	Median Y
All	50,000					
P1	42,456	84.91%	FSC-A	SSC-A	853,325	173,708
P2	40,061	94.36%	FSC-H	FSC-A	1,888,549	837,639
R3	19	0.05%	FITC-A	FSC-A	18,723	1,005,514
R4	40,050	99.97%	FITC-A	FSC-A	2,493	837,608

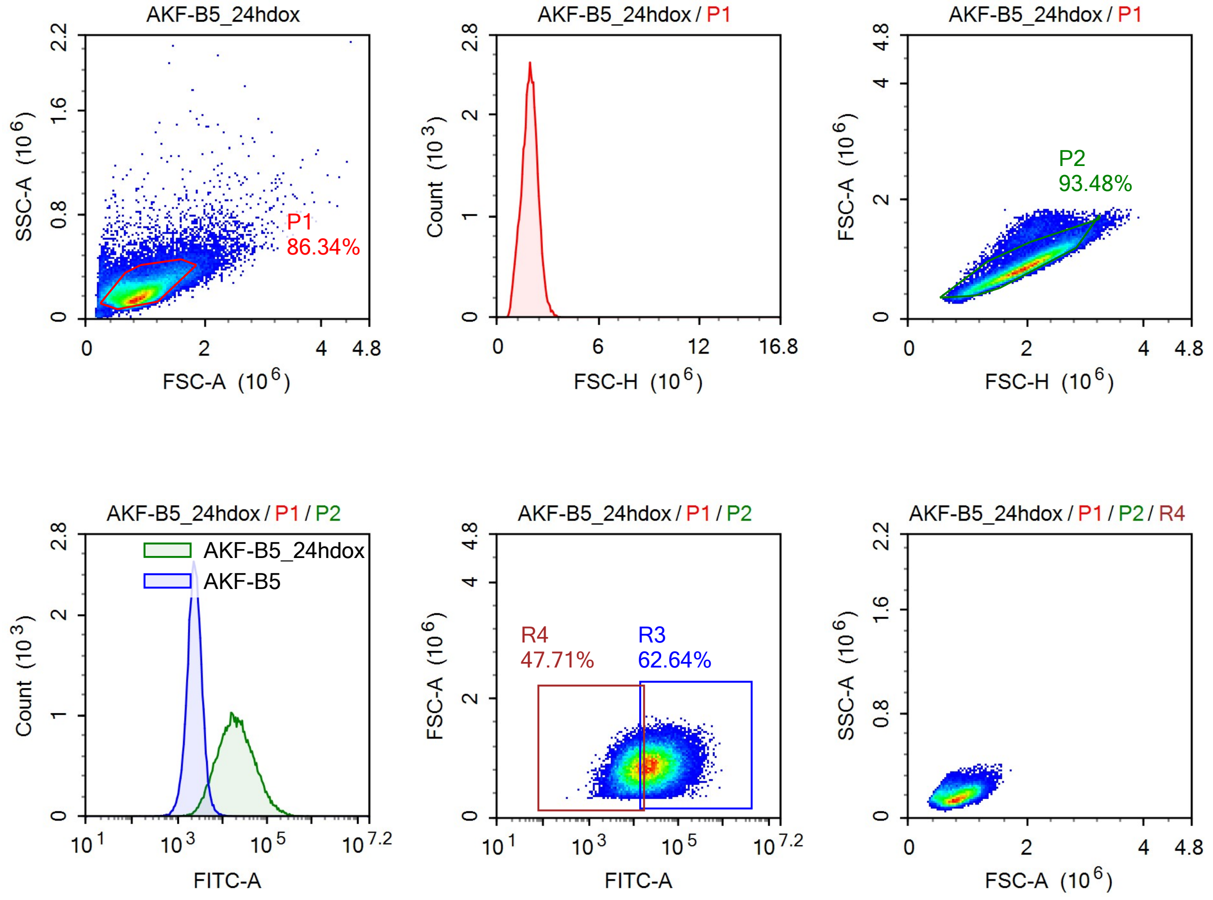

Sample Statistics of AKF-B5_24hdox

Gate	Count	% Parent	X	Y	Median X	Median Y
All	50,000					
P1	43,172	86.34%	FSC-A	SSC-A	862,576	174,646
P2	40,359	93.48%	FSC-H	FSC-A	1,915,589	843,751
R3	25,281	62.64%	FITC-A	FSC-A	31,905	877,849
R4	19,257	47.71%	FITC-A	FSC-A	10,521	800,275

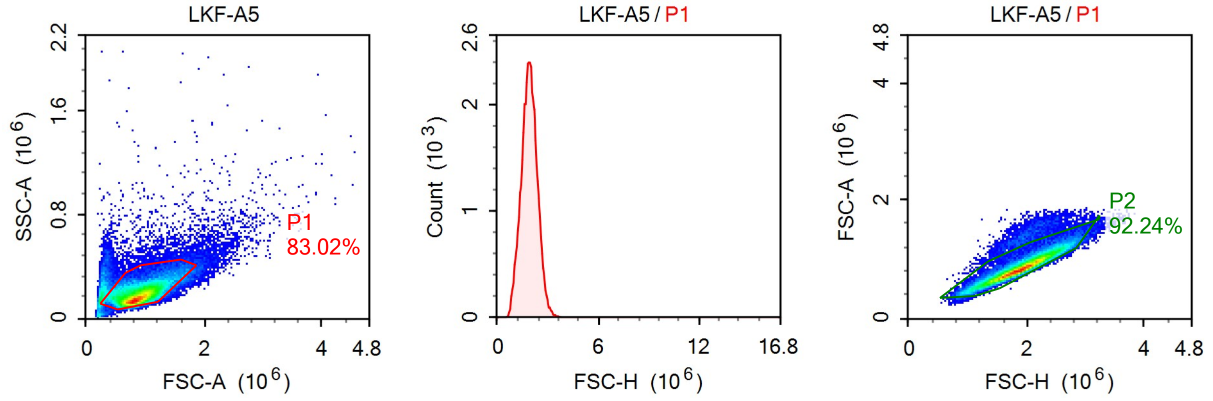

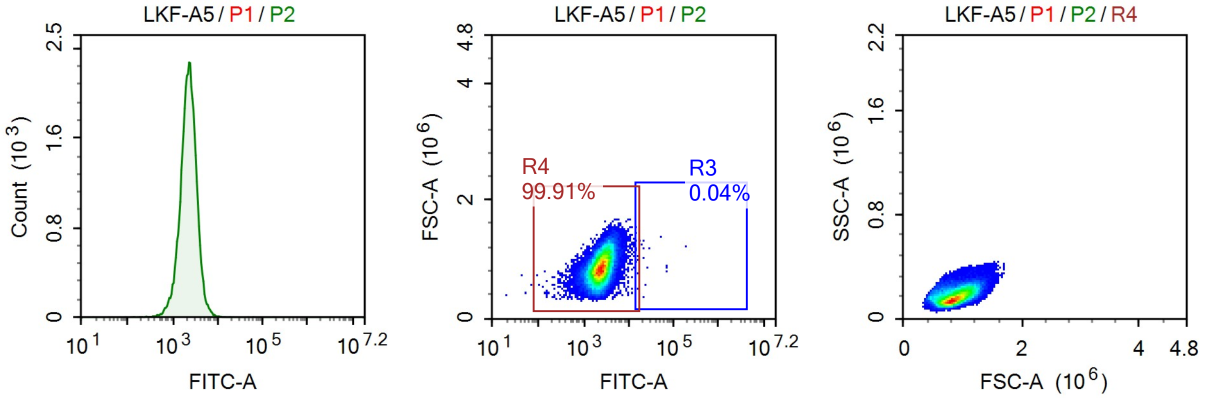

Sample Statistics of LKF-A5

Gate	Count	% Parent	X	Y	Median X	Median Y
All	50,000					
P1	41,511	83.02%	FSC-A	SSC-A	865,810	169,759
P2	38,288	92.24%	FSC-H	FSC-A	1,884,917	843,459
R3	17	0.04%	FITC-A	FSC-A	19,986	1,016,793
R4	38,255	99.91%	FITC-A	FSC-A	2,345	843,507

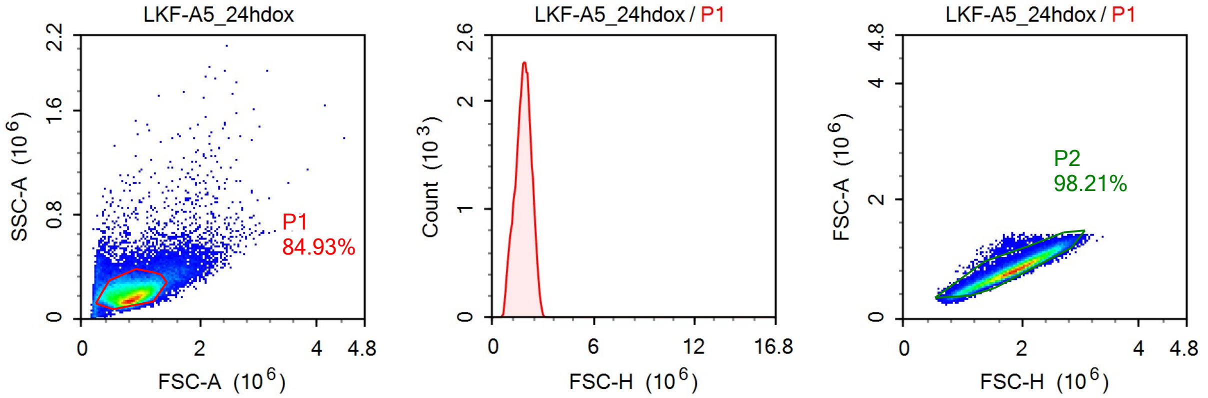

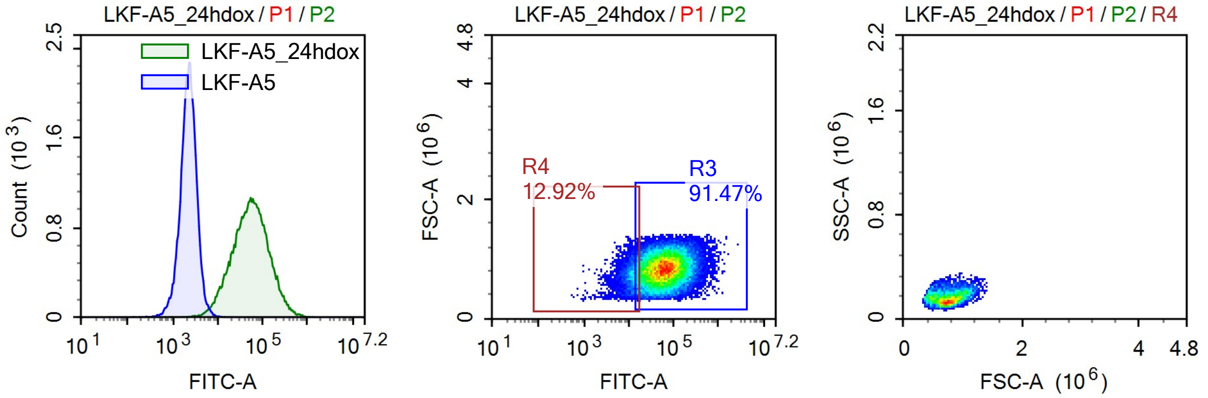

Sample Statistics of LKF-A5_24hdox

Gate	Count	% Parent	X	Y	Median X	Median Y
All	50,000					
P1	42,464	84.93%	FSC-A	SSC-A	816,360	169,444
P2	41,703	98.21%	FSC-H	FSC-A	1,850,250	814,237
R3	38,146	91.47%	FITC-A	FSC-A	61,693	823,212
R4	5,390	12.92%	FITC-A	FSC-A	13,088	724,465

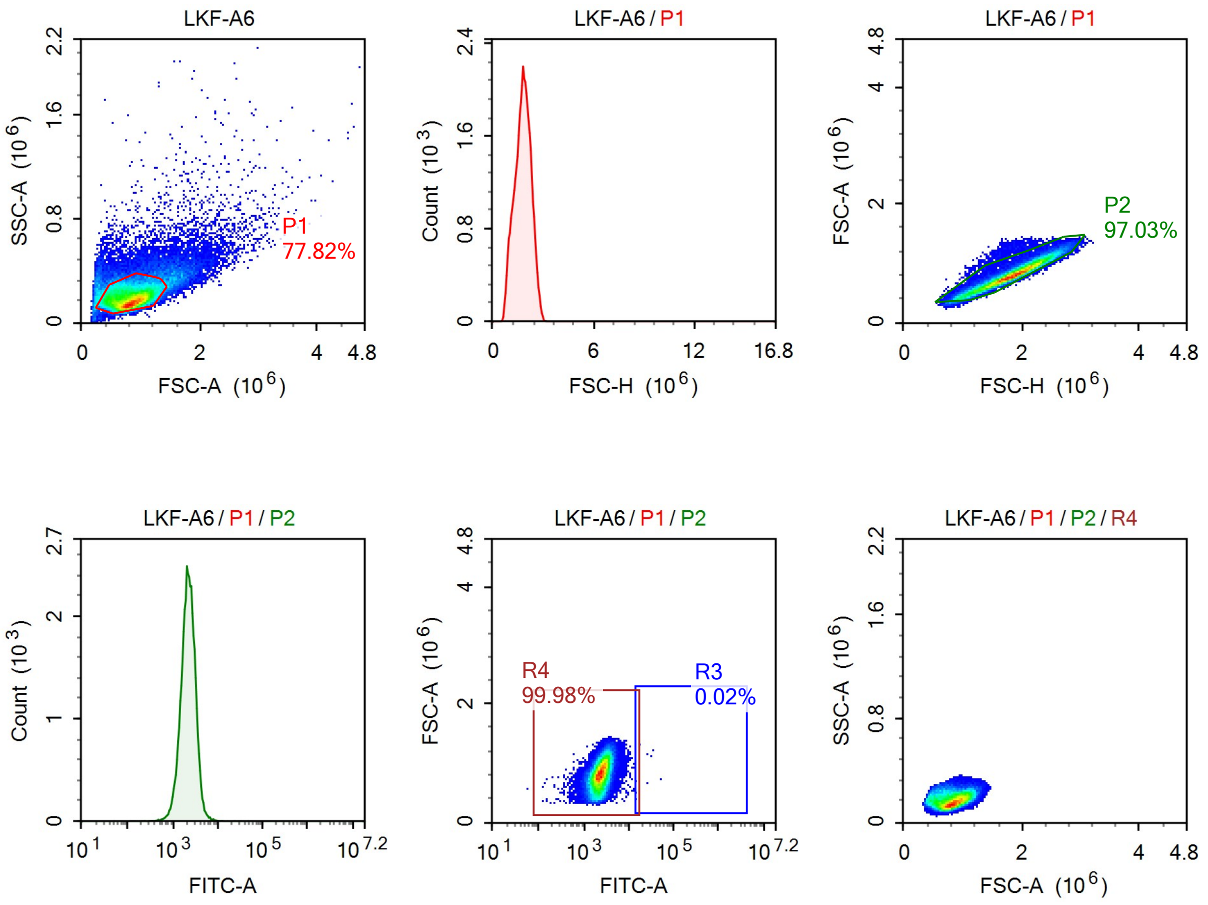

Sample Statistics of LKF-A6

Gate	Count	% Parent	X	Y	Median X	Median Y
All	50,000					
P1	38,910	77.82%	FSC-A	SSC-A	800,937	173,170
P2	37,754	97.03%	FSC-H	FSC-A	1,816,927	795,394
R3	6	0.02%	FITC-A	FSC-A	27,842	1,144,741
R4	37,746	99.98%	FITC-A	FSC-A	2,298	795,394

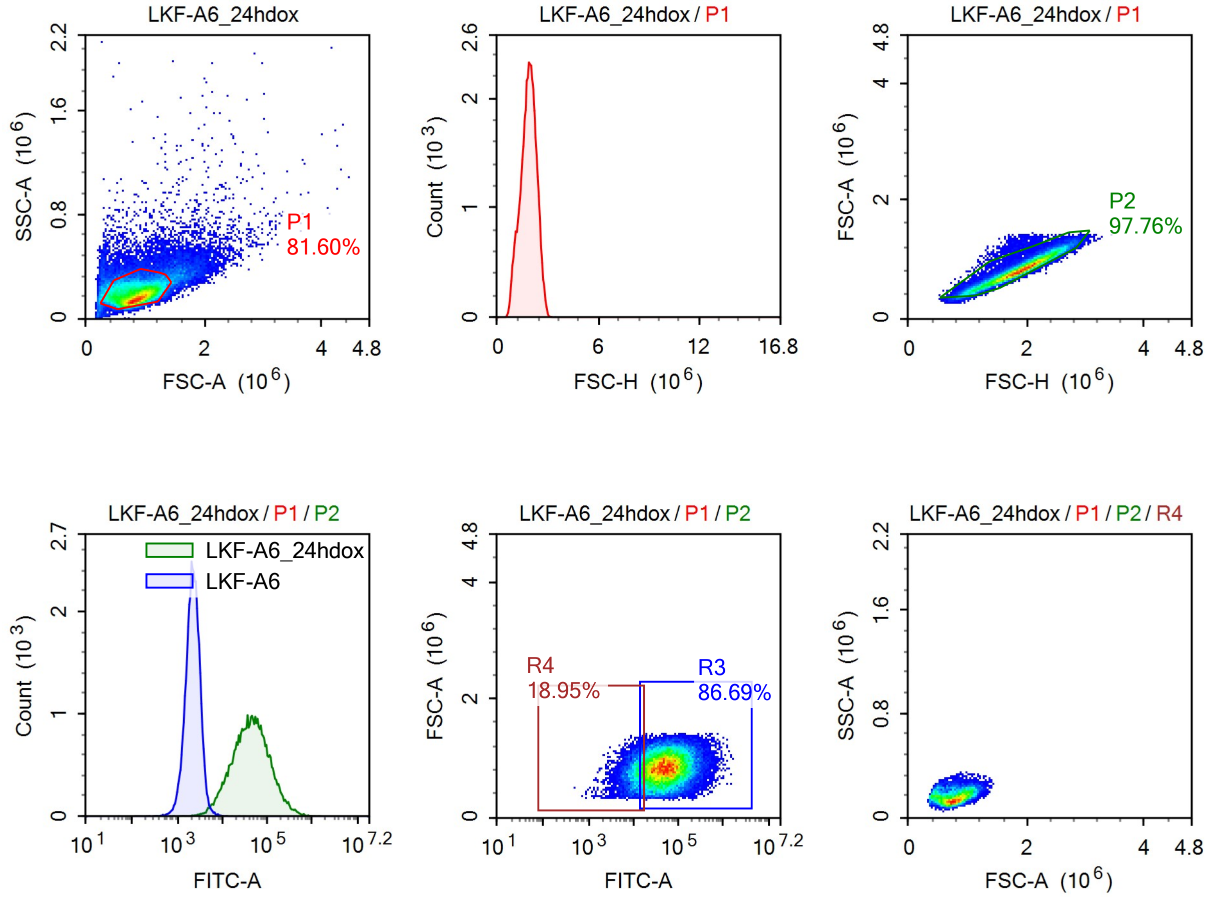

Sample Statistics of LKF-A6_24hdox

Gate	Count	% Parent	X	Y	Median X	Median Y
All	50,000					
P1	40,800	81.60%	FSC-A	SSC-A	820,255	168,528
P2	39,888	97.76%	FSC-H	FSC-A	1,879,076	816,636
R3	34,580	86.69%	FITC-A	FSC-A	52,328	830,825
R4	7,560	18.95%	FITC-A	FSC-A	12,384	739,877

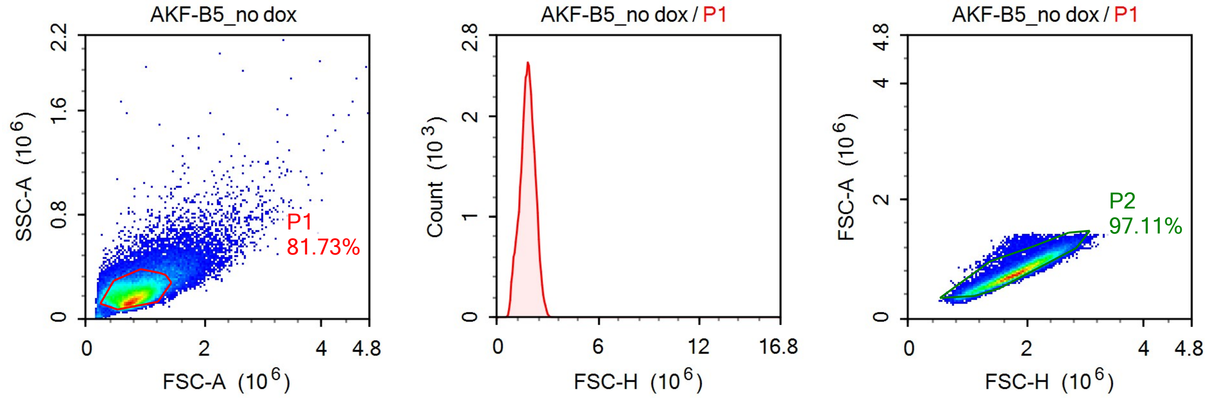

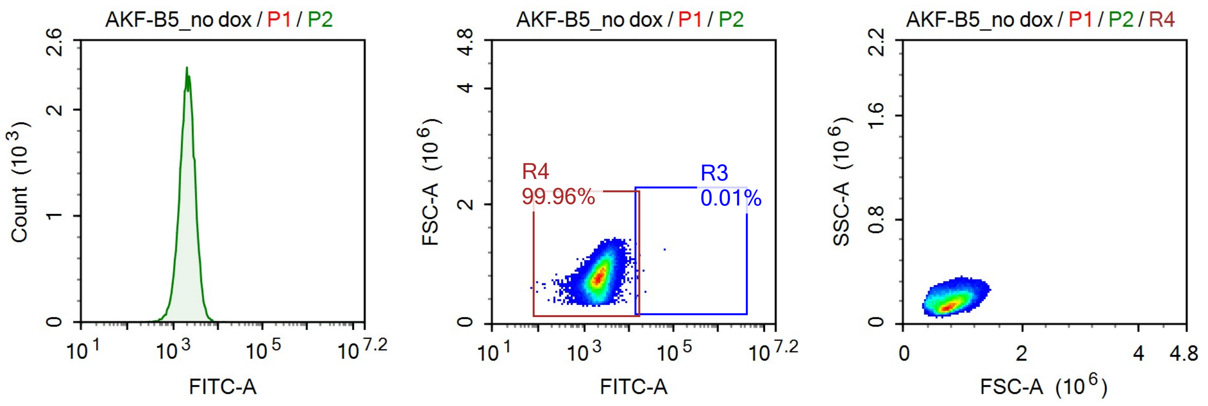

Sample Statistics of AKF-B5_no dox

Gate	Count	% Parent	X	Y	Median X	Median Y
All	50,000					
P1	40,865	81.73%	FSC-A	SSC-A	762,085	159,582
P2	39,685	97.11%	FSC-H	FSC-A	1,804,911	757,545
R3	4	0.01%	FITC-A	FSC-A	21,608	1,025,743
R4	39,670	99.96%	FITC-A	FSC-A	2,196	757,595

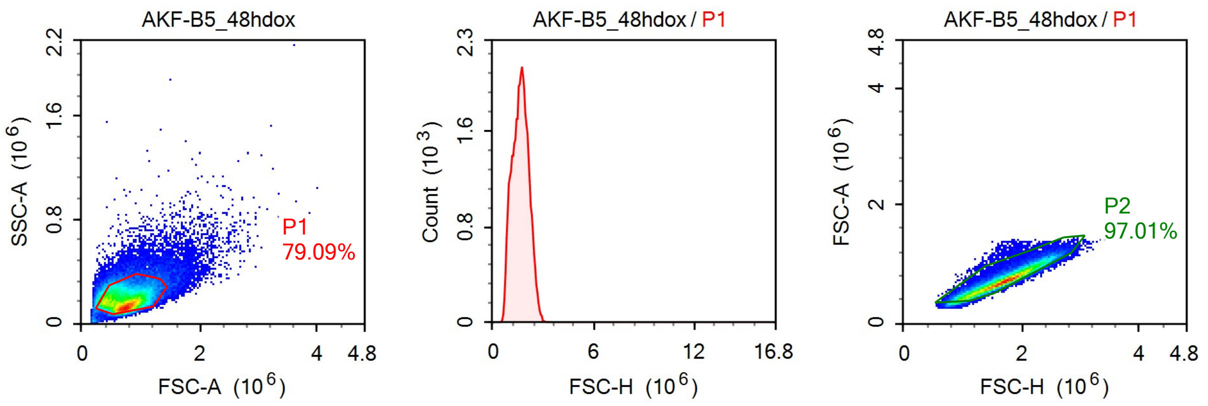

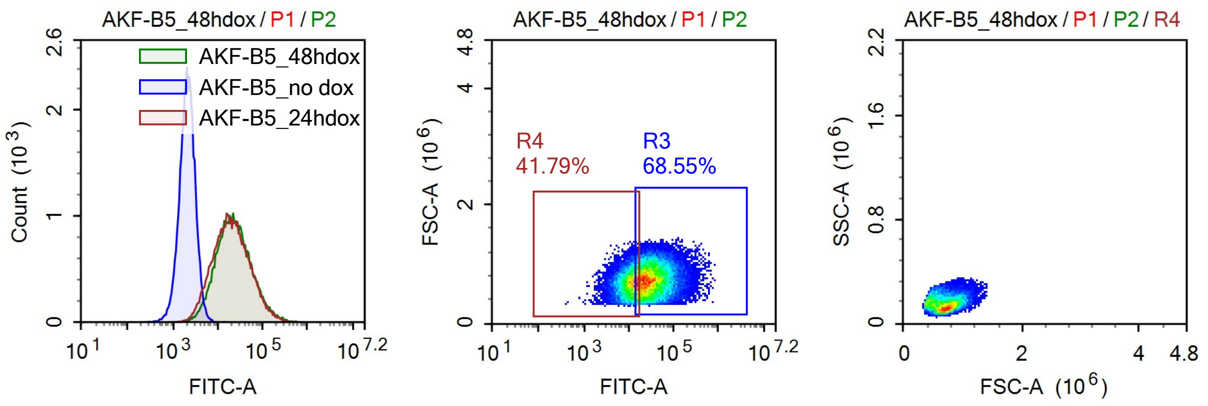

Sample Statistics of AKF-B5_48hdox

Gate	Count	% Parent	X	Y	Median X	Median Y
All	50,000					
P1	39,547	79.09%	FSC-A	SSC-A	711,643	160,731
P2	38,365	97.01%	FSC-H	FSC-A	1,666,592	709,011
R3	26,299	68.55%	FITC-A	FSC-A	32,769	732,312
R4	16,032	41.79%	FITC-A	FSC-A	11,499	671,552

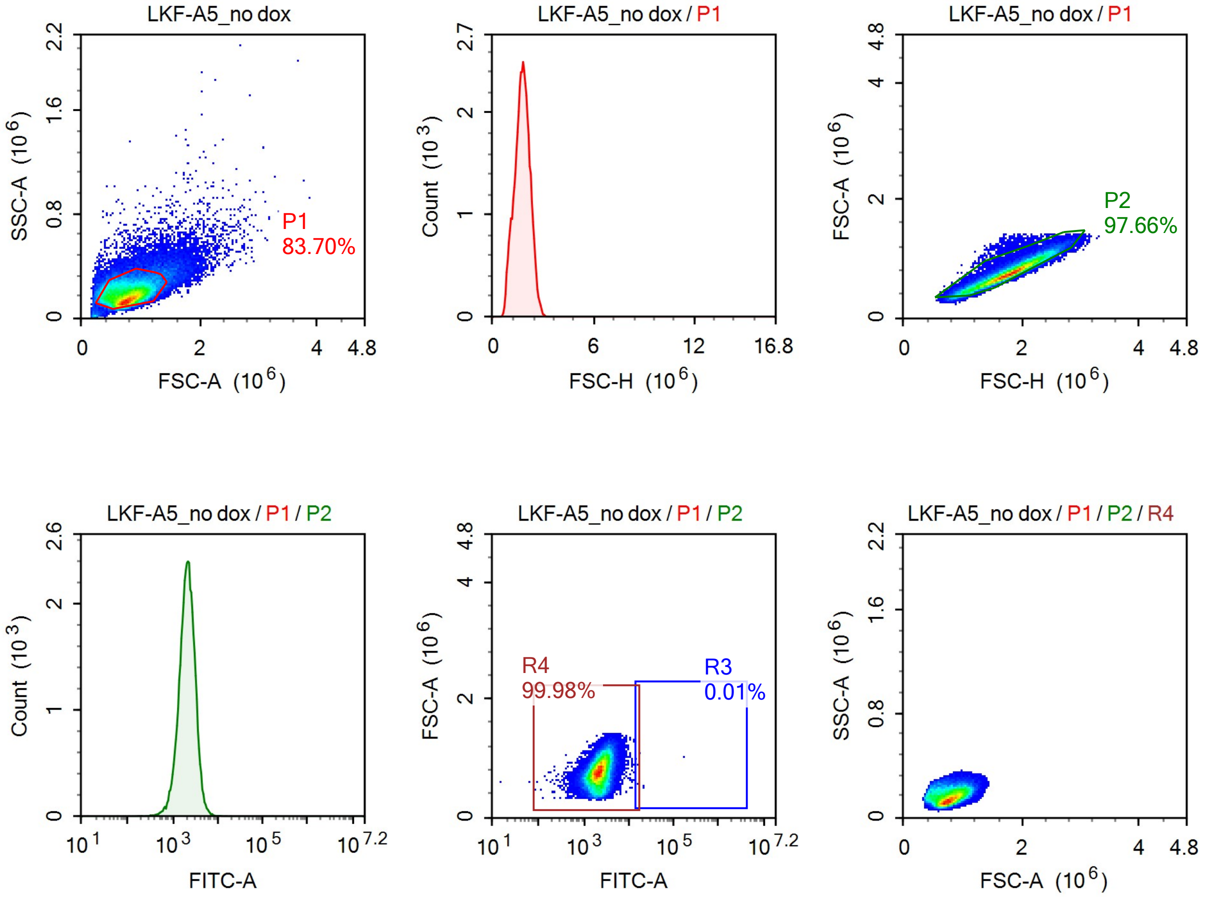

Sample Statistics of LKF-A5_no dox

Gate	Count	% Parent	X	Y	Median X	Median Y
All	50,000					
P1	41,851	83.70%	FSC-A	SSC-A	751,456	162,243
P2	40,870	97.66%	FSC-H	FSC-A	1,770,084	748,240
R3	6	0.01%	FITC-A	FSC-A	18,084	713,074
R4	40,860	99.98%	FITC-A	FSC-A	2,185	748,268

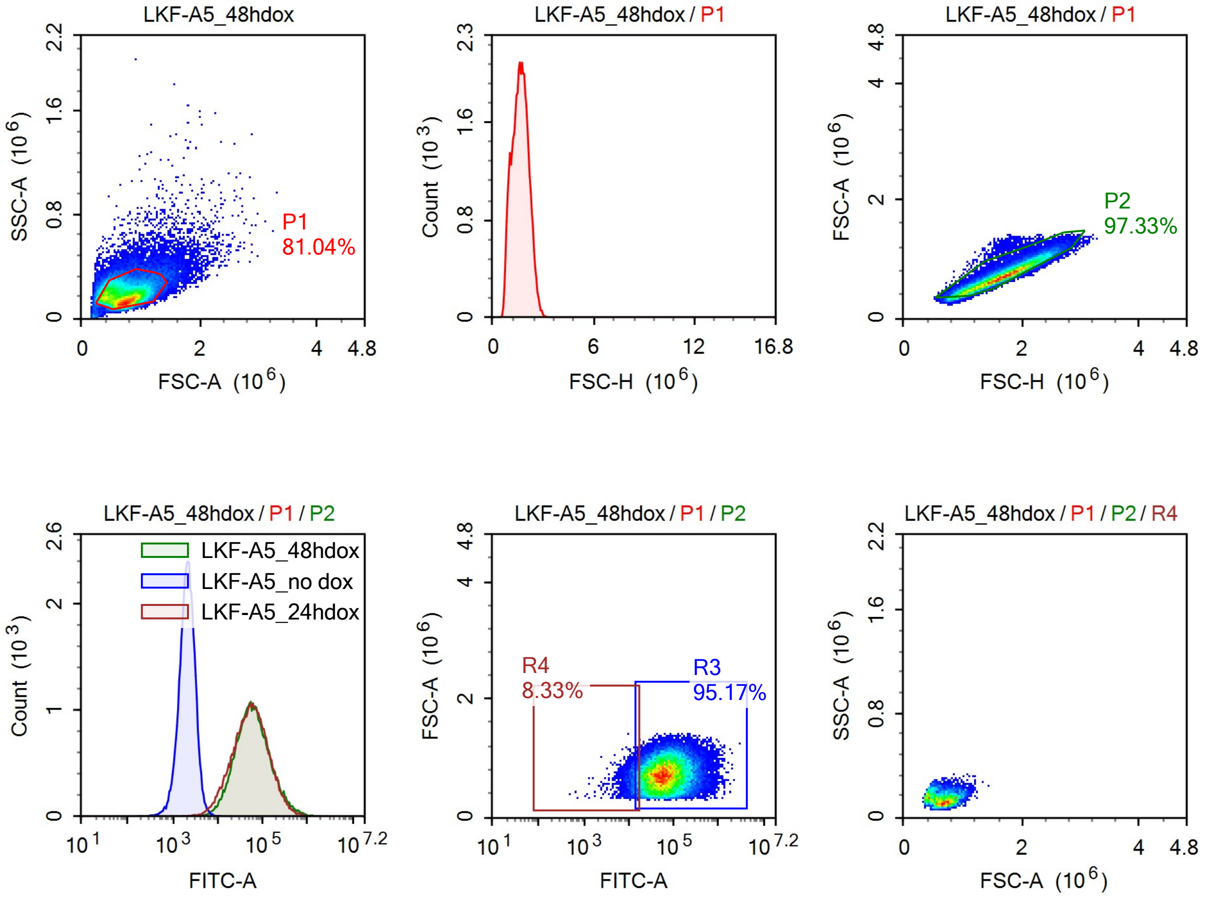

Sample Statistics of LKF-A5_48hdox

Gate	Count	% Parent	X	Y	Median X	Median Y
All	50,000					
P1	40,518	81.04%	FSC-A	SSC-A	706,620	159,370
P2	39,435	97.33%	FSC-H	FSC-A	1,647,083	704,905
R3	37,529	95.17%	FITC-A	FSC-A	63,407	708,807
R4	3,286	8.33%	FITC-A	FSC-A	14,093	636,526

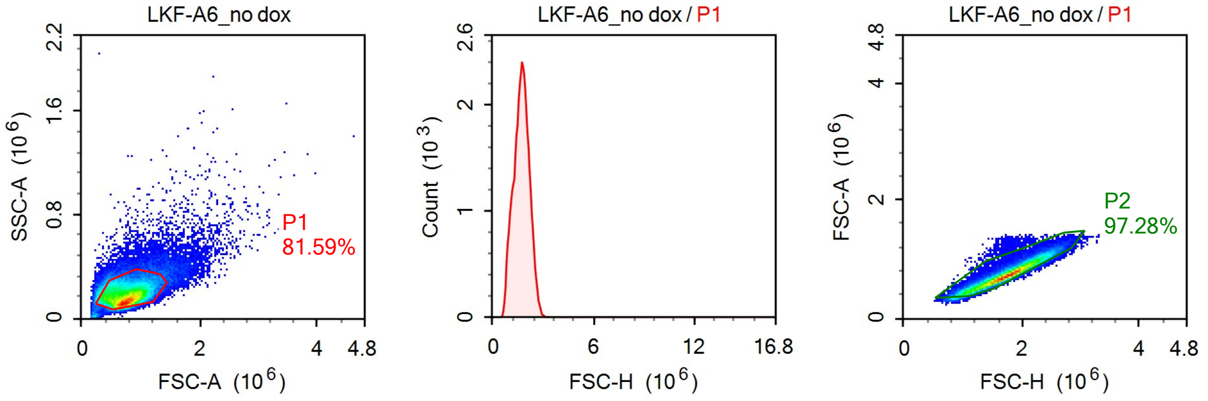

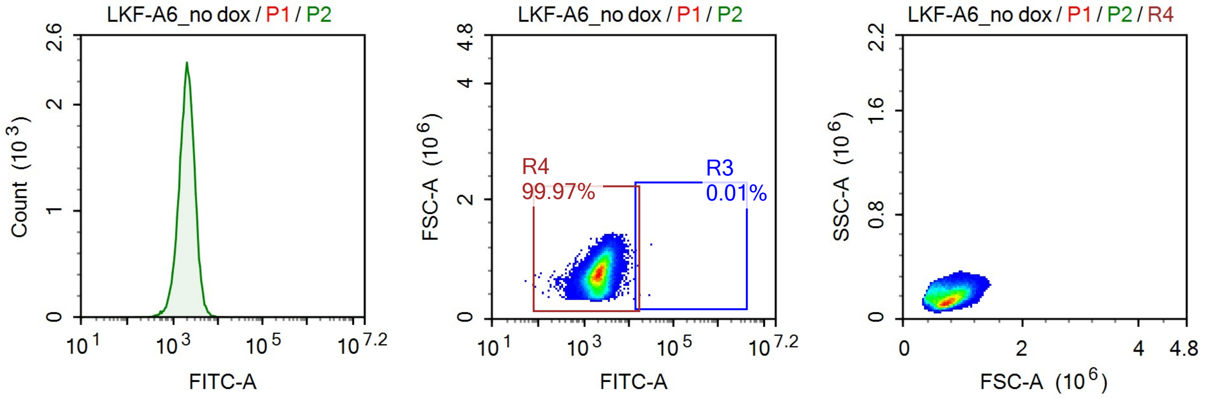

Sample Statistics of LKF-A6_no dox

Gate	Count	% Parent	X	Y	Median X	Median Y
All	50,000					
P1	40,793	81.59%	FSC-A	SSC-A	726,370	159,929
P2	39,684	97.28%	FSC-H	FSC-A	1,725,414	723,493
R3	4	0.01%	FITC-A	FSC-A	20,948	641,201
R4	39,672	99.97%	FITC-A	FSC-A	2,146	723,526

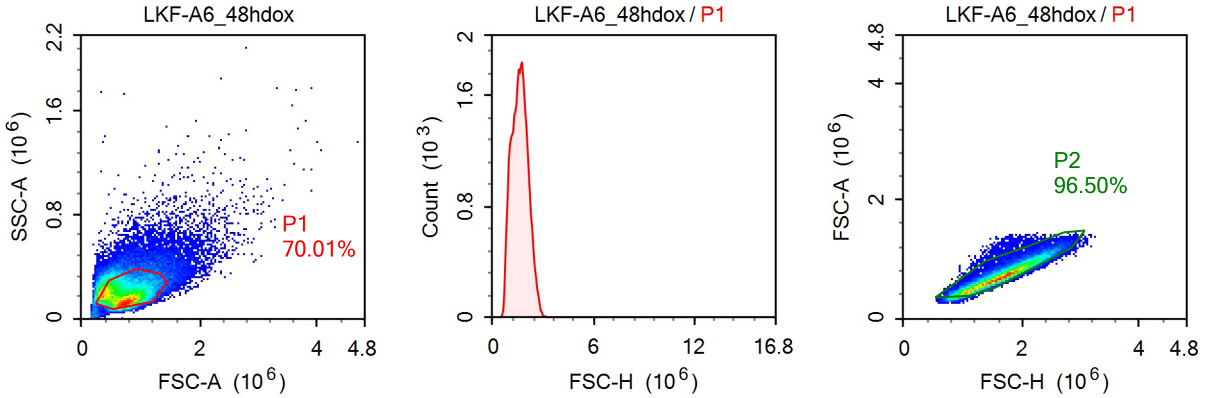

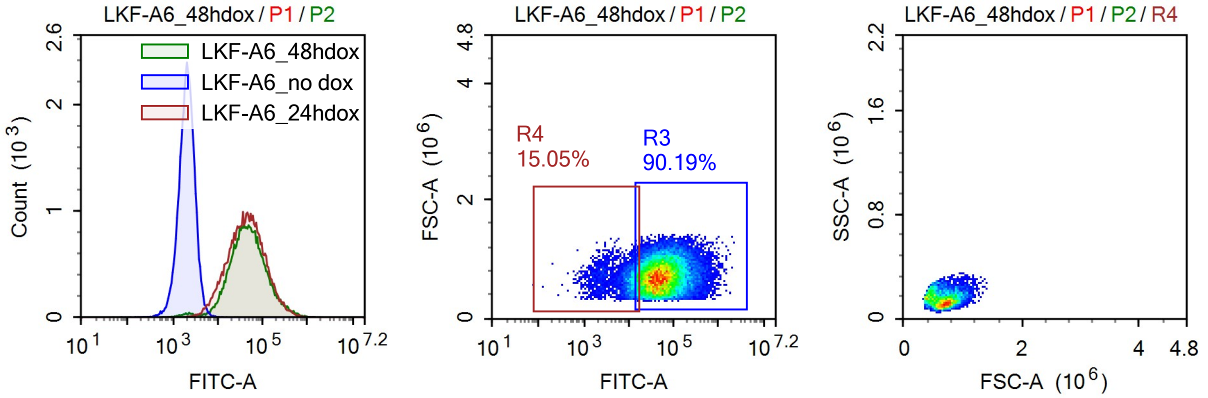

Sample Statistics of LKF-A6_48hdox

Gate	Count	% Parent	X	Y	Median X	Median Y
All	50,000					
P1	35,005	70.01%	FSC-A	SSC-A	698,923	162,322
P2	33,780	96.50%	FSC-H	FSC-A	1,614,502	694,896
R3	30,466	90.19%	FITC-A	FSC-A	52,627	700,996
R4	5,083	15.05%	FITC-A	FSC-A	13,061	648,800

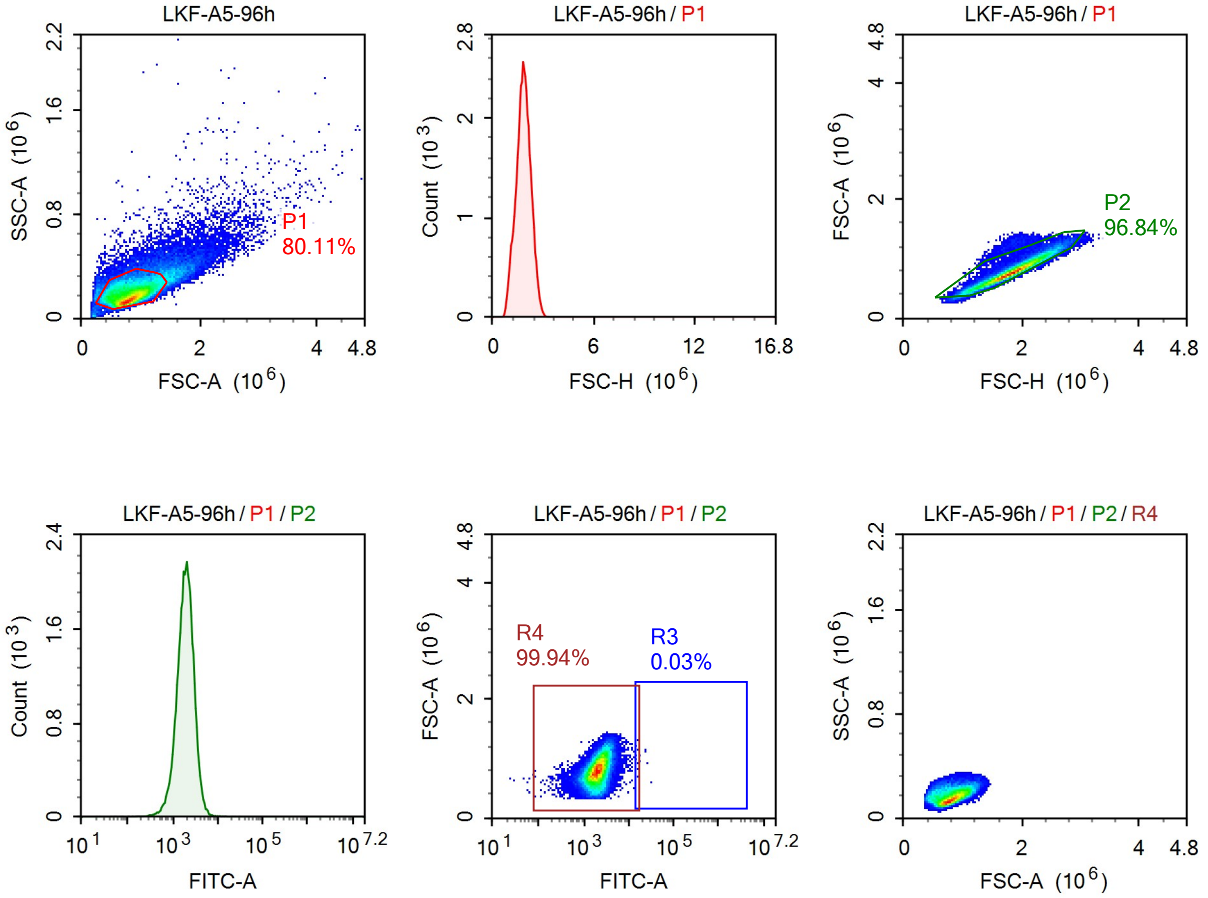

Sample Statistics of LKF-A5-96h

Gate	Count	% Parent	X	Y	Median X	Median Y
All	50,000					
P1	40,053	80.11%	FSC-A	SSC-A	788,259	166,772
P2	38,787	96.84%	FSC-H	FSC-A	1,838,282	781,008
R3	10	0.03%	FITC-A	FSC-A	16,144	1,007,159
R4	38,762	99.94%	FITC-A	FSC-A	2,010	781,121

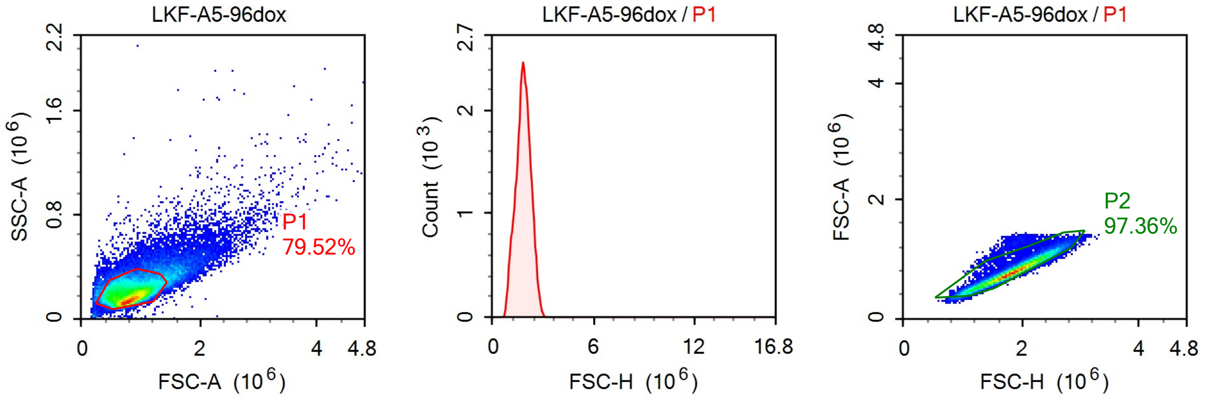

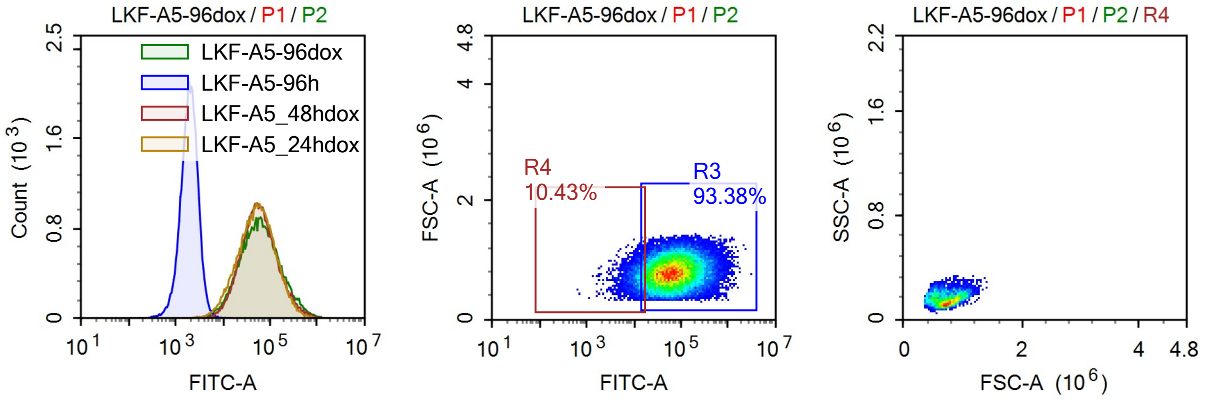

Sample Statistics of LKF-A5-96dox

Gate	Count	% Parent	X	Y	Median X	Median Y
All	50,000					
P1	39,761	79.52%	FSC-A	SSC-A	784,093	168,698
P2	38,713	97.36%	FSC-H	FSC-A	1,833,609	777,768
R3	36,152	93.38%	FITC-A	FSC-A	69,052	784,054
R4	4,039	10.43%	FITC-A	FSC-A	13,409	706,410

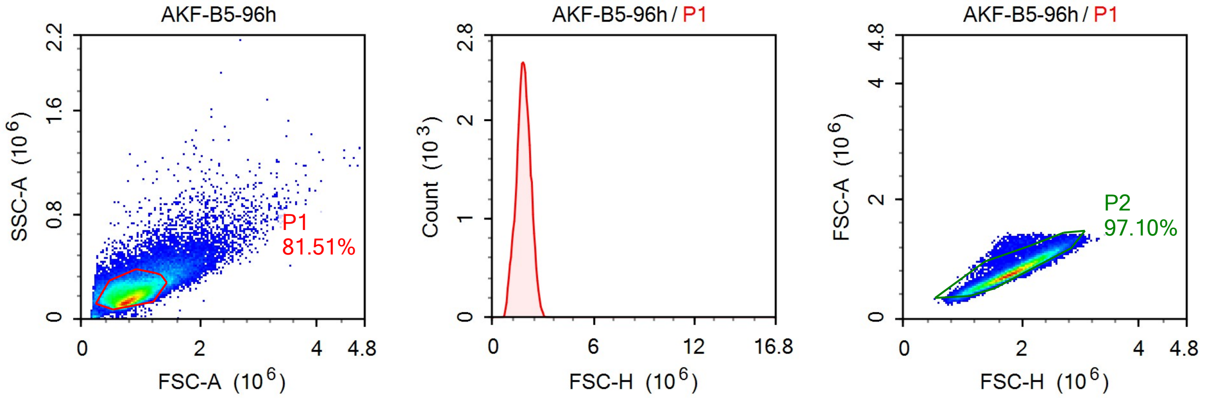

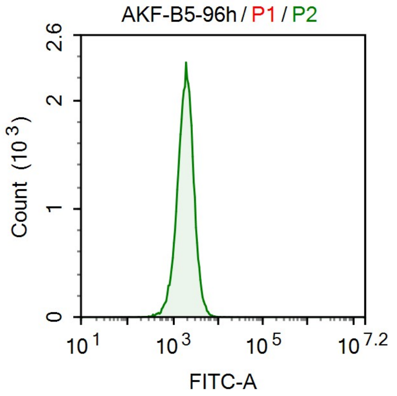

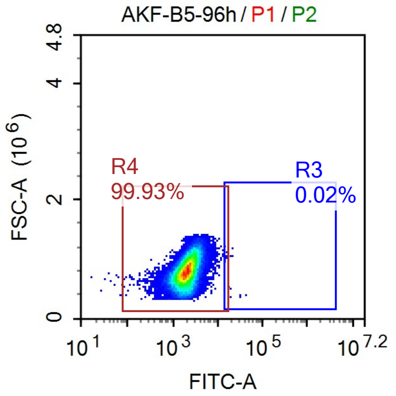

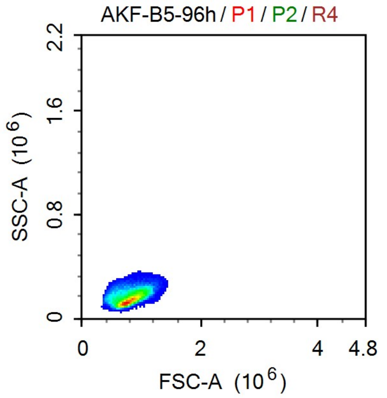

Sample Statistics of AKF-B5-96h

Gate	Count	% Parent	X	Y	Median X	Median Y
All	50,000					
P1	40,753	81.51%	FSC-A	SSC-A	778,087	160,533
P2	39,573	97.10%	FSC-H	FSC-A	1,833,623	772,296
R3	9	0.02%	FITC-A	FSC-A	19,930	480,621
R4	39,547	99.93%	FITC-A	FSC-A	1,992	772,431

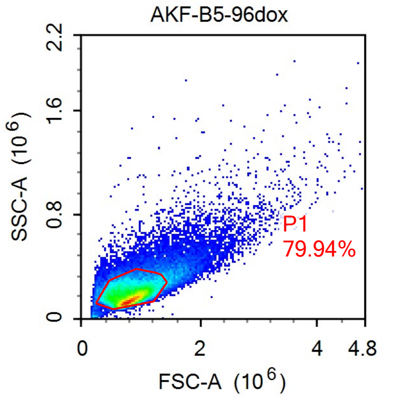

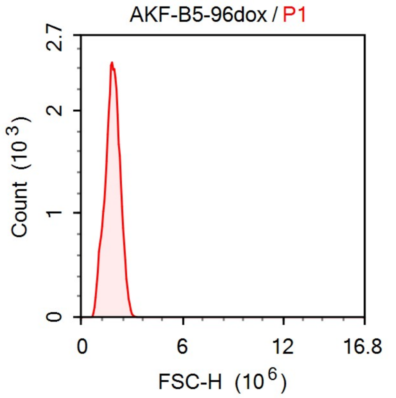

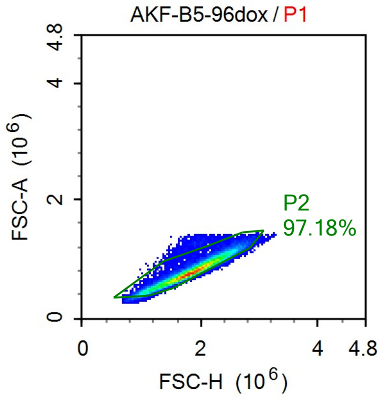

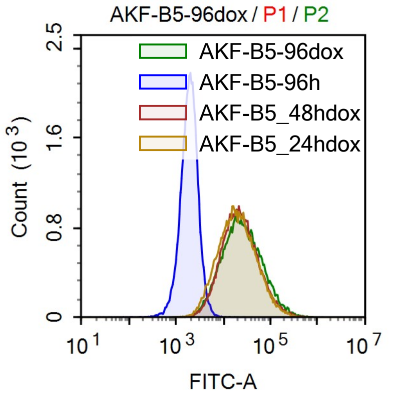

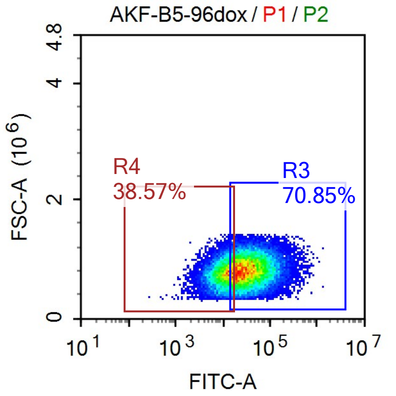

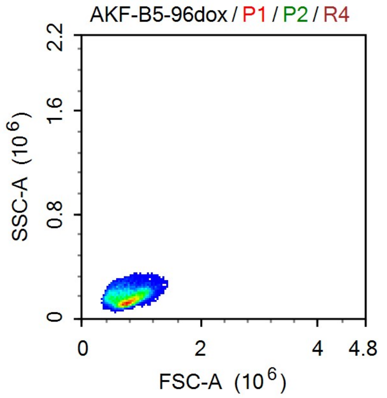

Sample Statistics of AKF-B5-96dox

Gate	Count	% Parent	X	Y	Median X	Median Y
All	50,000					
P1	39,971	79.94%	FSC-A	SSC-A	790,630	164,626
P2	38,843	97.18%	FSC-H	FSC-A	1,844,634	784,364
R3	27,522	70.85%	FITC-A	FSC-A	36,889	807,998
R4	14,980	38.57%	FITC-A	FSC-A	11,126	742,794

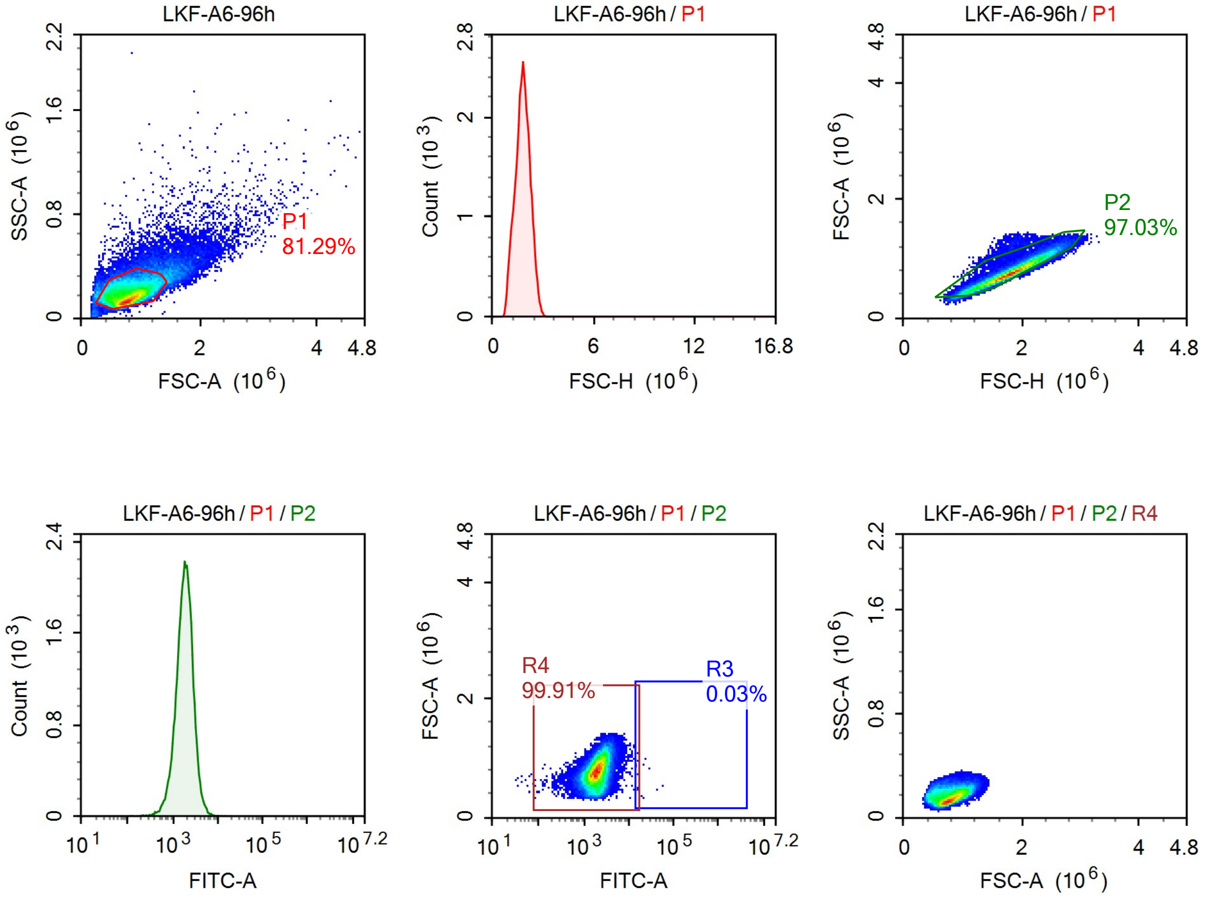

Sample Statistics of LKF-A6-96h

Gate	Count	% Parent	X	Y	Median X	Median Y
All	50,000					
P1	40,643	81.29%	FSC-A	SSC-A	760,672	164,764
P2	39,434	97.03%	FSC-H	FSC-A	1,794,809	754,844
R3	10	0.03%	FITC-A	FSC-A	20,378	671,959
R4	39,398	99.91%	FITC-A	FSC-A	1,933	755,032

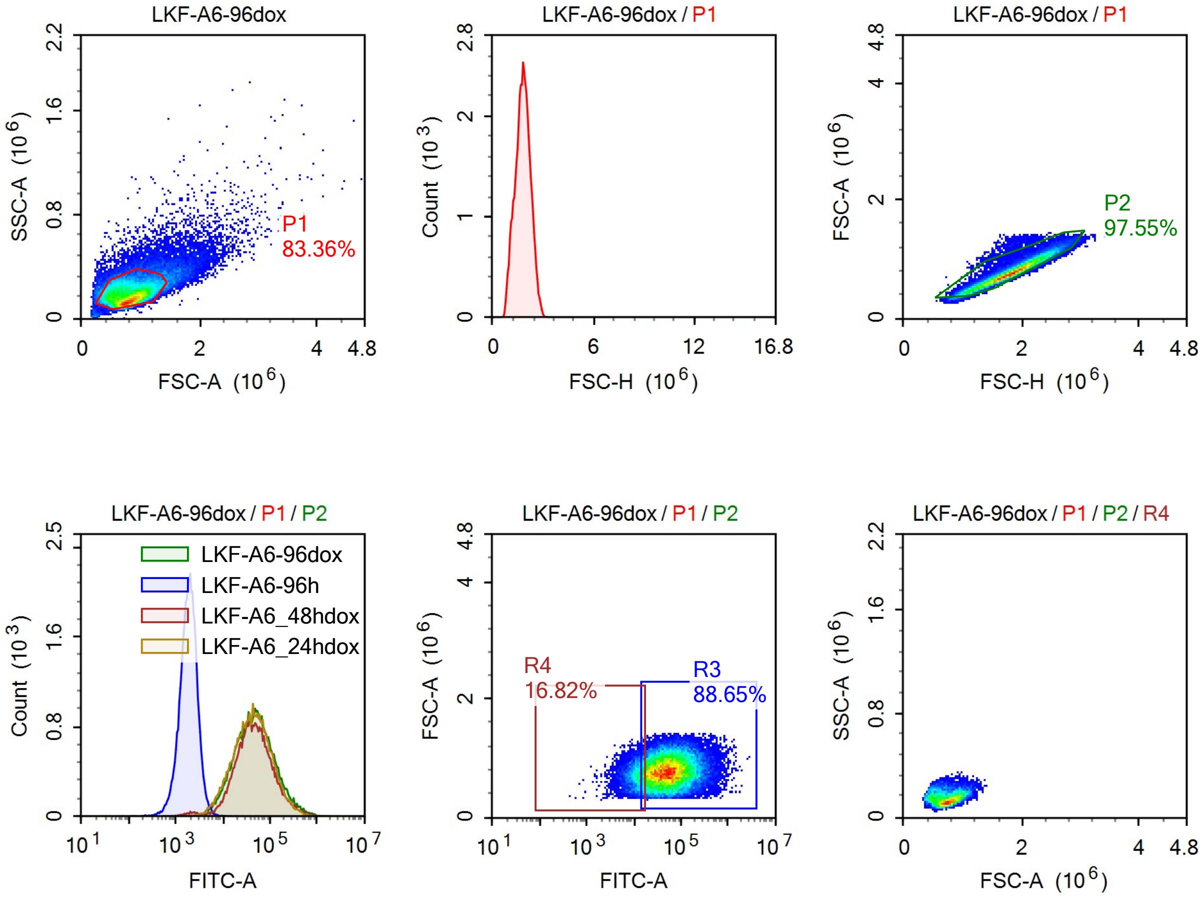

Sample Statistics of LKF-A6-96dox

Gate	Count	% Parent	X	Y	Median X	Median Y
All	50,000					
P1	41,680	83.36%	FSC-A	SSC-A	767,640	161,946
P2	40,658	97.55%	FSC-H	FSC-A	1,800,687	762,484
R3	36,044	88.65%	FITC-A	FSC-A	55,526	772,464
R4	6,837	16.82%	FITC-A	FSC-A	12,821	706,429
